# How well do various QM-derived net atomic charges reproduce the electrostatic potential surrounding a material across multiple geometric conformations?†

**DOI:** 10.1039/d4ra07900k

**Published:** 2025-07-07

**Authors:** Alma Carolina Escobosa, Thomas A. Manz

**Affiliations:** a Department of Chemical & Materials Engineering, New Mexico State University Las Cruces New Mexico 88003-8001 USA tmanz@nmsu.edu

## Abstract

Atom-centered point-charge models are computationally efficient and commonly used electrostatic models to build forcefields used in classical simulations of materials. To assess their performance, we evaluated various atomic charge assignment methods across the following material types: (a) organic molecules, (b) inorganic molecules, (c) heterodiatomic molecules, (d) transition metal complexes, and (e) nanoporous crystals. We compared 12 atomic charge assignment methods for molecular systems and 6 for nanoporous crystals. In this article, we introduce a computationally efficient quadrupole-dipole-resorption (QDR) method that improves the accuracy of stockholder-partitioning models (*e.g.*, DDEC6) for approximately reproducing the electrostatic potential and molecular dipole and quadrupole moments. For each electrostatic model, we computed the electrostatic potential's root-mean-squared error (RMSE) and relative RMSE (RRMSE) using the material's QM-computed electrostatic potential as a reference. The electrostatic RMSE and RRMSE were computed for a training dataset containing 21 geometric conformations per material and a validation dataset containing 20 new geometric conformations per material. Raincloud plots were prepared to visualize the resulting data distributions. For each charge assignment method in the nonperiodic materials, we also computed and compared the root-mean-squared charge transfer magnitude, correlations to other charge assignment methods, conformational sensitivity, *etc.* Among point-charge models, multiframe ESP methods provided the best overall accuracy for reproducing the electrostatic potential across different system conformations, but they require a training set containing many geometric conformations. The QDR-DDEC6 and CM5 methods provided good conformational transferability and electrostatic potential accuracy across various conformations even when trained only on a single optimized ground-state geometry. A Pareto plot was prepared illustrating the tradeoff between conformational sensitivity and accuracy for reproducing the electrostatic potential of individual conformations. Including atomic dipoles (*e.g.*, QDR-DDEC6_ad, DDEC6_ad, and MBIS_ad) greatly improved the electrostatic model for individual conformations, and QDR-DDEC6_ad outperformed all atom-centered point-charge models. We recommend that more computationally efficient methods be developed to use electrostatic models containing atom-centered point charges plus atomic dipoles in flexible forcefields. Finally, some electron-density partitioning approaches have the key advantage of providing accurate results even when applied to dense solids under high pressures, and we demonstrated this using 11 sodium chloride crystals having various stoichiometries.

## Abstract 2

This article compares the accuracy of various atomic charge schemes for reproducing the electrostatic potential surrounding organic, inorganic, and heterodiatomic molecules, transition metal complexes, and nanoporous solids.

## Graphical abstract

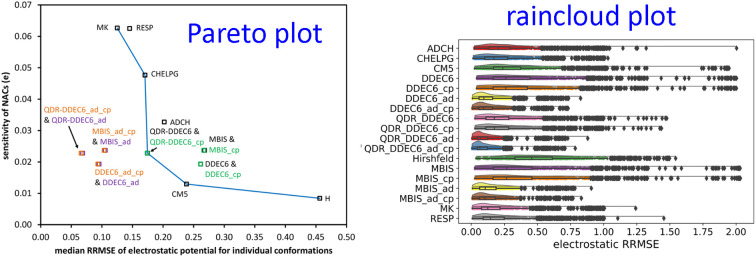

## 1. Introduction

Nonreactive flexible forcefields allow changes in bond lengths, bond angles, and dihedral values without forming any new chemical bonds or breaking any existing chemical bonds; however, a bond's length may be allowed to stretch to infinity.^1–5^ In general, such nonreactive flexible forcefields contain both bonded interactions and nonbonded interactions.^6,7^ Bonded interactions typically include bond stretches, angle bends, dihedral torsions, *etc.*^6–11^ Nonbonded interactions typically include electrostatic interactions,^12–16^ short-range repulsion,^17–19^ and fluctuating multipole dispersion^20–23^ interactions. These classical forcefields may be either polarizable or nonpolarizable.^24–28^

A key challenge is to build electrostatic models that are computationally efficient, easy to parameterize, and accurate across multiple geometric conformations of the material. The following questions should be considered when developing electrostatic models for flexible forcefields:

(1) How accurately does the classical electrostatic model reproduce the quantum-mechanically (QM) computed electrostatic potential surrounding the material across multiple conformations?^14,19,29,30^

(2) Do the assigned atom-in-material electrostatic descriptors (*e.g.*, atomic charges, atomic dipoles, *etc.*) accurately quantify the chemical states of atoms in the material?^30–36^

(3) How easy is it to construct and parameterize the classical electrostatic model? How much intellectual manual human labor and computer resources (*i.e.*, computational time and memory) are required to construct and parameterize the classical electrostatic model?

(4) Is the electrostatic model computationally cheap to use? Low computational cost is desirable to allow classical molecular dynamics (MD) and Monte Carlo simulations to sample more system configurations and larger (*i.e.*, containing more atoms in the simulation box) systems.

(5) Does the method work reliably across diverse material types?^31–33,37,38^ The following are worth considering: (i) Does the method work well for both surface atoms and buried atoms? (ii) Does the method work well for both non-periodic (*e.g.*, molecular) and periodic (*e.g.*, crystalline) materials? (iii) Does the method work well for all of the naturally occurring chemical elements? (iv) Does the method work well for ionic, covalent, and polar-covalent materials and for electrically conducting, semiconducting, and insulating materials?

(6) Does the classical forcefield include polarization effects explicitly, implicitly, or not at all? To achieve reasonable accuracy, the electrostatic model (including atomic charge values) must be appropriately chosen to avoid over-counting or under-counting the polarization effects.^13,39^

Electron density partitioning approaches have key advantages compared to other approaches to computing atom-in-material charges. By partitioning the material's total electron density distribution, 
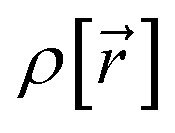
, among atoms in the material:1
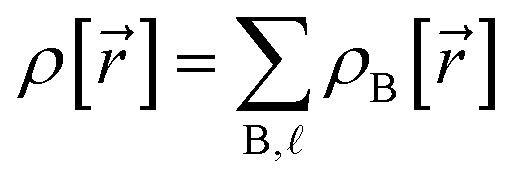
the electrostatic potential surrounding the material can be expressed exactly as the sum of a polyatomic multipole expansion and a polyatomic charge penetration expansion.^32,40^ In eqn (1), the sum over 
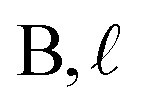
 means summing over all atoms {B} in the reference unit cell and their periodic images (if any). The multipole expansion of each atom in the material is determined by its net charge and its multipole moments: net atomic charge (*i.e.*, the monopole moment), atomic dipole, atomic quadrupole, atomic octupole, atomic hexadecapole, *etc.*^41–43^ The charge penetration expansion for each atom in the material comprises its spherically-averaged (aka ‘spherical’) cloud penetration plus non-spherical contributions of various orders.^17,44–48^

The quantum theory of atoms in molecules (QTAIM) partitions the material's electron density into non-overlapping compartments.^49,50^ Because its assigned atoms in materials are not approximately spherically symmetric, the QTAIM approach requires including atomic multipoles up to at least quadrupole order to approximately reproduce the electrostatic potential surrounding a material.^42,51–53^ For this reason, QTAIM net atomic charges are generally not considered to be viable candidates for atom-centered point-charge-only electrostatic models.^14,30^

Stockholder approaches partition a material's electron density into overlapping atoms.^31,43,54–57^ To be useful for constructing atom-centered point-charge-only electrostatic models for classical forcefields, a stockholder partitioning scheme should be defined to optimize the assigned atom-in-material electron density distributions to be approximately spherically symmetric.^34,43,57,58^

Besides enabling a formally exact expansion of the electrostatic potential surrounding a material, some electron density partitioning approaches allow a host of other key properties to be computed.^14,17–19,43,49,50,55,56,59–64^ Density-derived electrostatic and chemical (DDEC) approaches can be used to compute atom-in-material polarizabilities, dispersion coefficients, quantum Drude oscillator parameters, electron cloud parameters controlling short-range repulsion and charge penetration interactions, exponents for the Morse and Manz stretch potentials, and key chemical properties including bond orders and atomic spin moments.^7,20,21,31,32,37,38,65^

Is it possible to reproduce the polyatomic multipole expansion correctly up to quadrupole order using only atom-centered point charges (*i.e.*, monopoles) with all atomic dipoles and atomic quadrupoles set to zero in the electrostatic model? It can easily be shown this is not always possible. As a simple example, homodiatomic molecules such as H_2_, O_2_, N_2_, *etc.* have a non-zero quadrupole moment.^66^ Because the net charge is zero and the two atoms are equivalent, the assigned net atomic charge must be zero in these homodiatomics; consequently, the resulting atom-centered point charge yields both a zero molecular dipole moment and a zero molecular quadrupole moment. The homodiatomic's non-negligible quadrupole moment can be modeled using either an added off-site charge (*i.e.*, an added point charge placed at a non-nuclear position) or by atom-centered dipoles or by a bond-centered quadrupole. For example, a charge of −*q* could be placed at the bond's midpoint and a charge of *q*/2 could be placed at each atom's nuclear position to reproduce the homodiatomic's molecular quadrupole moment and net charge.^66^

This implies tradeoffs between the accuracy and the complexity of electrostatic models for constructing flexible forcefields. On the one hand, simple electrostatic models comprised of atom-centered point charges are desirable, because they are computationally fast. On the other hand, electrostatic models that correctly reproduce the local values of the electrostatic potential surrounding the material up to quadrupolar order are desirable based on accuracy considerations. Unfortunately, these two targets cannot generally be satisfied simultaneously. Specifically, if one restricts the electrostatic model to atom-centered point charges, then there are systems (*e.g.*, homodiatomic molecules) for which this restriction precludes reproducing the electrostatic potential accurately at quadrupolar order. Thus, one is generally forced to choose between atom-centered point-charge models that do not achieve quadrupolar accuracy and more complicated electrostatic models that achieve quadrupolar accuracy by including off-site charges^13,39,66,67^ or atomic multipoles^3,40,42,43^ or bond multipoles.

This manuscript compares and evaluates the suitability of several different methods to assign QM-derived atomic charges to construct electrostatic models using atom-centered point charges across multiple conformations of materials. This work is one of the most extensive comparisons of its type to date. The following particular aspects of this manuscript constitute a significant scientific advance over previous literature:

• We include an extremely diverse collection of materials in our test sets: organic molecules, inorganic molecules, heterodiatomic molecules, transition metal complexes, and nanoporous crystals. Moreover we selected diverse materials within each of these five test sets. In the nanoporous solids test set we included metal–organic frameworks, a zeolite, a nanotube, a metal–inorganic framework, a covalent organic framework, and aluminum phosphate crystal structures. In the organic molecules test set, we included representatives from more than 30 different classes of organic compounds. Elements were included from across the periodic table. We included covalent, polar-covalent, and ionic materials.

• For the larger materials, we included some functional groups that are rotatable leading to multiple conformers. Our training and validation datasets for these materials included various conformers. In addition to the ground-state (optimized) geometry, the training datasets included non-equilibrium structures generated using *ab initio* molecular dynamics (AIMD) calculations or conformation sampling. The validation datasets included geometries from new AIMD runs or conformation sampling that were separate from those used to generate the training datasets. This ensured that various configurations of each material were effectively sampled and tested.

• We tested a variety of different QM-derived charge assignment methods. For the non-periodic materials (*i.e.*, molecules), we even included several multiframe approaches (*e.g.*, multiframe MK, multiframe CHELPG, and multiframe RESP) for which the charges were optimized to simultaneously minimize the root-mean-squared error (RMSE) of the electrostatic potential over the valid grid points across multiple geometries (aka ‘frames’) of the material.

• For each material, we evaluated three different sets of atomic charges for the non-multiframe charge assignment methods: charges specific to each geometric configuration, charges averaged over all geometric configurations, and charges from the optimized ground-state geometry.

• For the DDEC6 and MBIS methods, we also quantified the effects of including atomic dipoles and/or spherical electron cloud penetration potentials.

• For stockholder electron-density partitioning approaches such as DDEC6, we introduce a new method to partly resorb the atom-in-material quadrupole and dipole moments into the atom-centered point charges to improve the accuracy of atom-centered point-charge models derived using stockholder partitioning. The new QDR-DDEC6 charges achieve a near-optimum combination of electrostatic accuracy and conformational transferability.

• Our data analysis goes beyond averages, medians, and standard deviations by including full raincloud plots of the electrostatic RMSE and RRMSE data. This clarifies the behavior of outliers, which are discussed in detail.

• In a manner analogous to ref. 14, we use Pareto plots to analyze trade-offs between accuracy for reproducing the electrostatic potential of individual molecular conformations and changes in the assigned atomic charge values across molecular conformations. In a manner analogous to ref. 35, 36 and 68, we statistically analyze correlations between various charge assignment schemes to identify the most confluent^36^ approach.

## 2. New quadrupole-dipole-resorbed density-derived electrostatic and chemical (QDR-DDEC) charges

### 2.1 Motivation

We begin with the parable of the robot golfers as a useful analogy to better understand the history of methods that assign QM-derived atomic charges in chemical systems to parameterize atom-centered point-charge models for constructing classical forcefields. In this parable, the task is to design a robot that excels at playing golf. In this hypothetical game of golf, the robots compete not to get the golf ball into the hole in the fewest number of strokes, but rather to see which robot can get the golf ball closest to the hole regardless of the number of strokes taken. As described in Section 3.3 below, the accuracy of a parameterized atom-centered point-charge model is quantified by how closely it reproduces the QM-computed electrostatic potential surrounding the material. This is analogous to how far the golf ball ends up away from the target hole. This kind of scoring is necessary, because it is usually not possible to make the atom-centered point-charge model's electrostatic potential exactly match the QM-computed electrostatic potential. In this analogy, the different holes within the same golf course represent different geometric configurations of the same bonded cluster (*i.e.*, molecule, nanostructure, or chemical specie), while different golf courses represent different chemical species. Since we want to use the same forcefield parameters to describe all of the molecule's configurations, we seek a solution that also maximizes conformational transferability of the assigned atomic charge values.

The earliest robot prototypes that are built may not be sophisticated or refined. At first, progress could be achieved by building a robot prototype that can hit the golf ball perhaps with little control over the specific direction or distance of the golf ball's trajectory. This is analogous to the earliest atomic population analysis methods which lacked a complete basis set limit, such as Mulliken^69^ population analysis introduced in 1955 and Lowdin^70–72^ population analysis. The explicit basis set dependence of these population analyses methods could be likened to the robot hitting the golf ball too hard (causing the golf ball to travel too far of a distance) when the robot's battery is freshly charged and hitting the golf ball too softly (causing the golf ball to travel too short of a distance) when the robot's battery is wearing down. Although these results are reproducible, they are far from desirable.

In the 1970's, Bader^73^ and Hirshfeld^54^ developed atomic population methods that had complete basis set limits. By analogy, this could be likened to adding a voltage regulator to the robot so that its battery's output is nearly constant thereby facilitating more consistent results. Bader population analysis is also called the quantum theory of atoms in molecules (QTAIM^49,50,61^) and forms a subpart of the quantum chemical topology (QCT^42,51,64,74^) theoretical framework. Compared to earlier approaches, QTAIM and Hirshfeld population analysis more consistently predicted the charge transfer direction between atoms in a material. This is analogous to the robot more consistently hitting the golf ball in the general direction of the hole. However, for purposes of constructing atom-centered point-charge electrostatic models used in classical forcefields for molecules, Hirshfeld partitioning typically severely underestimates the charge transfer magnitude (analogous to the robot hitting the golf ball too softly) while QTAIM typically severely overestimates the charge transfer magnitude (analogous to the robot hitting the golf ball too hard).^36,75–77^ In fairness, it should be pointed out that Hirshfeld partitioning and QTAIM were not designed for that purpose. Specifically, one needs to include the atomic multipoles to achieve reasonable electrostatic potential models using QTAIM.^42,51,52^

A subsequent major development on this topic came in the form of electrostatic potential fitting (ESP) charges. In the decades since the late 1970's, a series of protocols were introduced to compute ESP charges.^78,79^ In this article, for nonperiodic materials (*i.e.*, molecules) we used charges from electrostatic potentials using a grid (CHELPG),^80^ Merz–Singh–Kollman electrostatic potential fitting (MK),^81,82^ and restrained electrostatic potential fitting (RESP).^83^ For periodic materials, we used repeating electrostatic potential extracted atomic (REPEAT)^84^ and RESP charges. These top-down approaches empirically optimize the atomic charge values to closely reproduce the QM-computed electrostatic potential surrounding a material. In other words, the atomic charge values are optimized based on the material's geometry and the target QM-computed electrostatic potential (a top-down approach) rather than partitioning the material's QM-computed electron-density distribution 
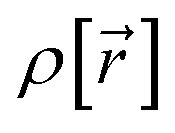
 (a bottom-up approach). In the robot golfer analogy, this optimization process is akin to having the robot make empirical adjustments based on trial-and-error ‘practice’ shots without any underlying programming of Newton's laws of motion. For example, if the ‘practice’ shot falls short of the hole, the robot has programming that redoes the shot from the starting tee using a larger force or a different club to hit the golf ball. In this way, the robot eventually optimizes the shot so that the ball lands close to the target hole. An advantage of this top-down optimization approach is that it gets the ball close to the hole regardless of the wind conditions and Coriolis force.

Single-frame ESP methods optimize the ESP charges for an individual geometric configuration of the bonded cluster. In this analogy, the robot golfer uses the top-down trial-and-error ‘practice’ shots to refine the golf swing for an individual hole on the golf course. When the robot goes to the next hole on the golf course (*i.e.*, the next geometric configuration of the molecule), it retains no information from the previous holes (*i.e.*, other geometric configurations of the molecule). Because the robot golfer starts from scratch every time, this trial-and-error process has poor transferability. Even if two holes on the golf course are similar to each other, the robot golfer may eventually reach dramatically different solutions; for example, using dramatically different clubs to hit the golf ball in each case. This is akin to the ESP methods assigning dramatically different atomic charge values for two different geometric configurations of the same molecule. In other words, single-frame ESP methods have poor conformational transferability.^83,85^

Multiframe ESP methods try to resolve this problem by using a top-down approach to simultaneously optimize the atomic charge values across multiple geometric configurations of the bonded cluster.^29^ A multiframe ESP method uses the same atomic charge values across all geometric configurations of the bonded cluster.^29^ This is analogous to the robot golfer using the same golf club for all 18 holes in the golf course and taking practice swings on all 18 holes before updating the swings to their final values on any of the holes.

Except for the two-stage (‘two-stroke’) RESP method, all of the strategies described above hit the golf ball from the starting tee to its final position in one stroke. Several strategies can be devised in which the robot golfer uses two or more strokes to try to successively move the ball closer to the hole. The iterative Hirshfeld^76^ (IH, introduced in 2007), iterated stockholder atoms^57^ (ISA, introduced in 2008), early generations of the density-derived electrostatic and chemical^30,34^ (*e.g.*, DDEC/c1, DDEC/c2, DDEC3), and minimal basis iterative stockholder^14^ (MBIS, introduced in 2016) methods used successive charge cycles to iteratively update the electron density partitioning until some internal self-consistency condition is met. These approaches were ‘bottom-up’ in the sense that they started with the material's QM-computed electron density distribution and then partitioned it into overlapping atoms according to some defined criterion. This is analogous to the robot golfer using Newton's laws of motion to predict the golf ball's trajectory and successively hitting the golf ball in multiple strokes until it lands where the robot predicts the hole is. In this case, the robot's programming is not sophisticated enough to account for wind speed, wind direction, or the Coriolis force due to Earth's rotation. So, the golf ball does not end up exactly where the hole is.

For some materials, ISA partitioning involved an extremely large number of iterations (*e.g.*, >1000) to reach convergence.^34^ Since each iteration (aka ‘charge cycle’) can introduce a small error, these errors can build up to substantial values if the number of iterations is large. By analogy, if the robot golfer hits the ball in more than 1000 strokes to reach the predicted location of the target hole, even a slight miscalibration might successively build up to a large error over such a large number of strokes. The IH and early DDEC (*e.g.*, DDEC/c1, DDEC/c2, DDEC3) methods do not converge to a unique solution in some materials (*i.e.*, their optimization landscape is not convex in some materials).^30,32^

Charge model 5 (CM5) introduced in 2012 is a ‘two-stroke’ method. In CM5, first a standard Hirshfeld partitioning is performed, then an empirical correction is added.^75^ This empirical correction intends to reproduce reference molecular dipole moments and was parameterized to a training dataset.^75^ For a pair of atoms A and B in a material, this CM5 correction depends on which chemical elements they are and the distance between them.^75^ In the robot golfer analogy, the CM5 scheme is analogous to the robot first hitting the ball in the general direction of the hole (using Hirshfeld partitioning) and then hitting a second stroke that attempts to correct for the average shortfall (of the Hirshfeld method) and the average wind speed (*i.e.*, average dipole polarization of the atoms). As shown in Section 5 below, our computed results showed the CM5 method is highly effective under normal conditions but fails for some materials at extremely high pressures.

The DDEC6 approach avoids the convergence problems of early DDEC (*e.g.*, DDEC/c1, DDEC/c2, DDEC3) methods by optimizing a series of 14 Lagrangians defining seven charge partitioning steps.^37^ By limiting the number of charge partitioning steps to seven, this avoids the problem of small errors building up to large errors over hundreds of charge cycles.^32^ Amongst methods developed to date, DDEC6 is one of the most chemically accurate, widely applicable, and confluent charge partitioning methods.^31–33,35–38^ In the robot golfer analogy, this is a ‘seven stroke’ method in which the robot uses Newton's laws of motion to compute trajectories and successively hits the ball seven times towards the predicted location of the hole.

As mentioned in the Introduction above, each electron-density partitioning approach generates a polyatomic multipole expansion that exactly reproduces the QM electrostatic potential outside of the material's electron density distribution. In this polyatomic multipole expansion, the first term is due to atom-in-material charges, the second term is due to atom-in-material dipole moments, the third term is due to atom-in-material quadrupole moments, and so forth.

By Gauss's law of electrostatics, if each atom in the material had an exactly spherically-symmetric electron density distribution, then the atom-centered point-charge model would be exact and all of the atom-in-material dipole moments and higher-order multipole moments would be zero. Due to the build-up of electrons in covalent bonds and other angular distortions of atoms, it is typically not possible to assign atom-in-material electron distributions that are exactly spherically symmetric. Usually, the atom-in-material dipole moments and higher-order multipole moments can be optimized to be small in magnitude but not necessarily zero. Even if one were hypothetically able to construct an electron-density partitioning scheme that delivered the ‘closest-to-perfect’ or ‘best’ atom-in-material charge values, each of the assigned atom-in-material dipole moments would not be zero for such a scheme in some materials.

Accordingly, if we construct an atom-centered point-charge electrostatic model by truncating the polyatomic multipole expansion at monopole order and discarding all of the terms due to atom-in-material dipoles and higher-order multipoles, this would introduce a truncation error even for the hypothetically ‘closest-to-perfect’ or ‘best’ electron-density partitioning method. Returning to the analogy of the robot golfer, the atom-in-material charges are analogous to computing the ball's trajectory in the absence of wind and in the absence of the Coriolis force due to the Earth's rotation, while the atom-in-material dipole moments and higher-order multipole moments account for these effects. If the robot aims the ball ‘perfectly’ or ‘directly’ at the hole, then it will actually not land in the hole, because the wind and Coriolis force will blow it off course. So, to make the ball land as close to the hole as feasible, it must actually be hit towards a direction beside the hole so the wind and Coriolis force will blow it sideways towards the hole during flight.

This implies a strategy for constructing atom-centered point-charge models in which one starts with atom-in-material charge values that are derived from a chemically-accurate electron-density partitioning approach (such as DDEC6) and then adds an adjustment for the discarded atom-in-material multipole moments. Most importantly, this does not imply that the starting atom-in-material charge values are chemically inaccurate, because such an adjustment would still be required for the hypothetically ‘closest-to-perfect’ or ‘best’ electron-density partitioning method that delivers chemically-accurate net atomic charge values. For this reason, we believe it is not appropriate to claim such an adjustment ‘corrects’ the net atomic charge values, but rather to state that part of the discarded atom-in-material dipole and quadrupole moments have been ‘resorbed’ into the atom-centered point-charge values. This is not intended to make the atom-in-material charges ‘more chemically accurate’ but rather to recover some of the electrostatic potential accuracy lost by not explicitly including the atom-in-material multipole moments—a subtle but critically important distinction.

The atomic dipole corrected Hirshfeld (ADCH) method starts with the Hirshfeld atom-in-material charges and then adds atom-centered point-charge corrections to account for discarding the Hirshfeld-assigned atom-in-material dipoles.^86^ Two aspects of the ADCH method are notable. First, since the Hirshfeld method systematically underestimates charge transfer magnitudes, these atomic dipole corrections are often large. Second, the linear equation system defining the ADCH charges can have an ill-conditioned coefficients matrix,^86,87^ which makes the ADCH charge values extremely sensitive to changes in the molecule's conformation in some cases.

As shown in the following sections, we solved these two problems as follows. To solve the first problem, we start with a more chemically accurate stockholder partitioning method (*e.g.*, DDEC6) instead of Hirshfeld partitioning. To solve the second problem, we introduce a new optimization scheme that always yields a linear equation system having a well-conditioned coefficients matrix. In addition to solving those two problems, our new approach also has the advantage of partly resorbing both the atom-in-material quadrupole moments and atom-in-material dipole moments into the atom-in-material charge values in a way that optimizes conformational transferability.

### 2.2 Definition *via* optimization function

#### 2.2.1 System notation and input information

Here, we use a capital letter (*e.g.*, A) to denote an atom in the reference unit cell and a lowercase letter (*e.g.*, b) to denote a translated image of an atom (due to periodic boundary conditions, if any). Let b be an atom image in the material whose nuclear position is2
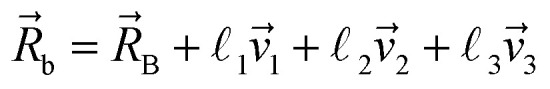
where 
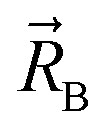
 is the nuclear position of atom B in the reference unit cell. The system can have 0, 1, 2, or 3 periodic boundary conditions, with 
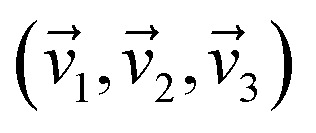
 being the corresponding periodic lattice vectors (if any). The translation indices of image b are the integers 
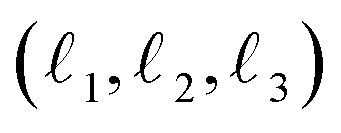
. For a direction without periodic boundary conditions, 
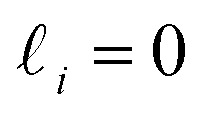
. For a direction with periodic boundary conditions, 
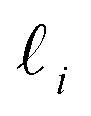
 is an integer between minus infinity and plus infinity. The translation images 
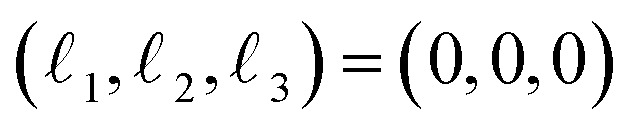
 correspond to atom images in the reference unit cell. The vector, distance, and direction from atom A to image b are3
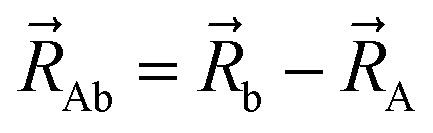
4
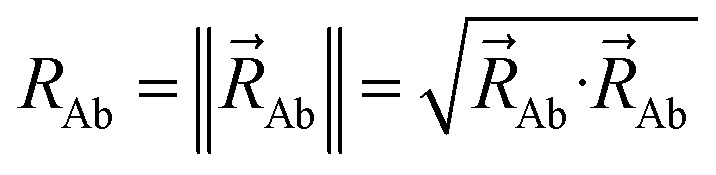

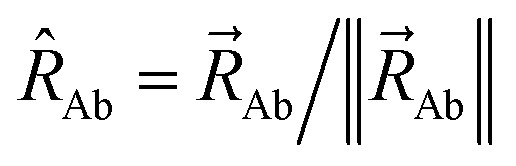

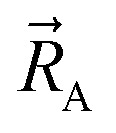
 is the nuclear position of atom A, and5
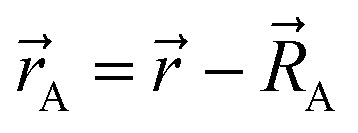
is the relative position of grid point 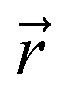. The vector from position 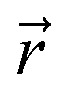 to image b is:6
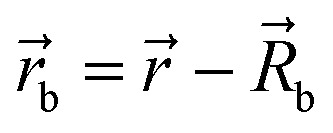
and *r*_b_ is the corresponding distance.

Our quadrupole-dipole-resorption (QDR) charge scheme starts with the set of net atomic charges ({*q*_A_}), atom-in-material dipole moments 
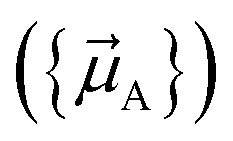
, traceless atom-in-material quadrupole moments, and overlap populations ({OP_Ab_}) computed *via* a stockholder^54^ electron-density partitioning approach:7
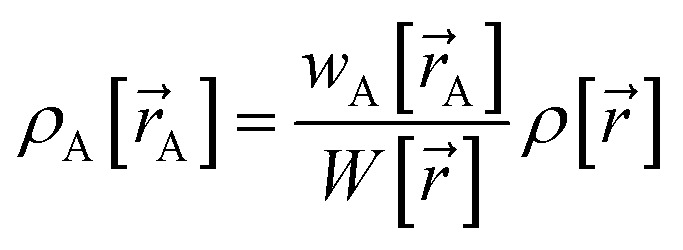
8
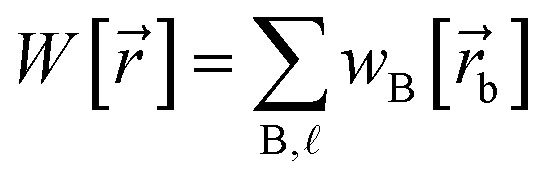
9
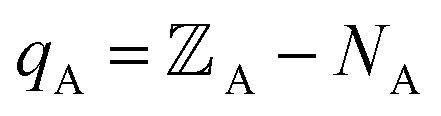
10
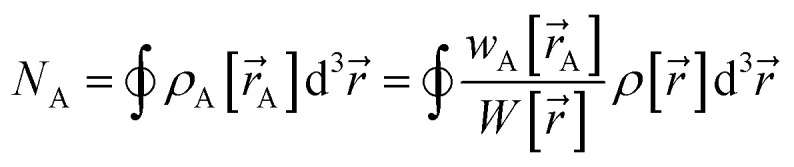
11
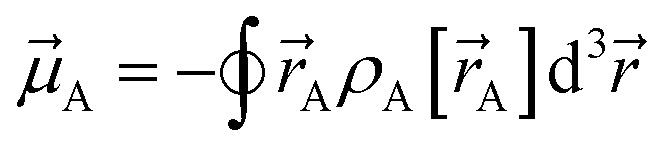
12
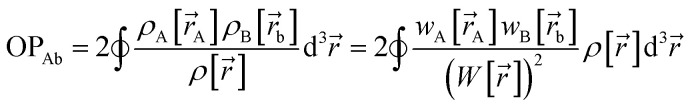

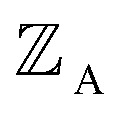
 is the atomic number of atom A. The summed overlap population (SOP) for atom A13
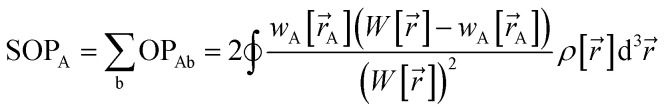


Since14
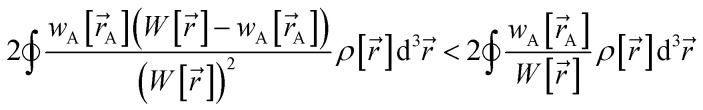
SOP_A_ is clearly bounded by

15 0 ≤ SOP_A_ < 2*N*_A_

The atom-in-material traceless quadrupole moment tensors were computed as16
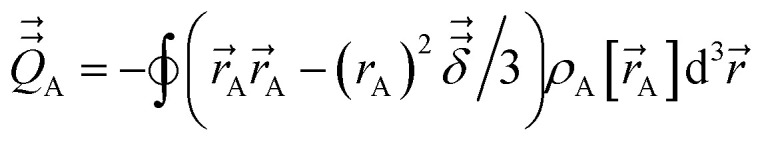
where 
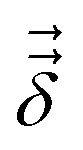
 is the identity tensor. In the literature, several different conventions are in use for defining the traceless quadrupole moments. For example, the Buckingham^88^ convention uses17
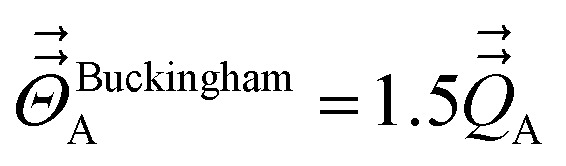
We used the 
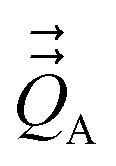
 convention of eqn (16), because it is also the definition used by the Gaussian16 (ref. 89) program. Multiwfn^90,91^ used the Buckingham convention.

While any stockholder partitioning scheme could be used as inputs, for best results a chemically accurate and broadly applicable stockholder partitioning scheme (such as DDEC6) should be chosen.

#### 2.2.2 Resorption scheme that preserves net charge, dipole moment, and quadrupole moment

As stated in the Introduction, electron-density partitioning methods yield polyatomic multipole expansions that exactly reproduce the electrostatic potential outside the material's electron density distribution. In a system with multiple expansion sites, the polyatomic multipole expansion of the electrostatic potential can be re-written in different ways that are equivalent up to some finite order. In this work, we introduce polyatomic multipole re-expansions that shift electric charge and dipole moments between adjacent expansion sites (*e.g.*, atom A and an adjacent image b) in a way that leaves all of the following unchanged: (i) the system's net charge, (ii) the system's total dipole moment vector, and (iii) the system's overall traceless quadrupole moment tensor.

This will be true regardless of which particular position is chosen as the coordinate system's origin for the purpose of computing the system's overall multipole moments. However, values of the system's overall non-leading multipole moments can depend on the origin's choice. For example, if a molecule has a non-zero molecular dipole moment, the value of the molecule's overall traceless quadrupole moment tensor depends on the choice of origin. We seek re-expansions such that when keeping the coordinate system's origin fixed (*i.e.*, unchanged), the original polyatomic multipole expansion and its re-expansion yield the same values for the system's overall charge, dipole moment vector, and traceless quadrupole moment tensor.

We specifically seek polyatomic multipole re-expansions that satisfy the following design criteria:

(a) The re-expansions will be designed so that the sum of residual atom-in-material dipole moment vectors and sum of residual atom-in-material quadrupole moment tensors are approximately zero to the extent feasible within limitations imposed by the system's geometry. This design criteria intends to maximize the accuracy of the associated atom-centered point-charge model for approximately reproducing the system's total dipole moment vector and overall traceless quadrupole moment tensor.

(b) The re-expansions will achieve excellent conformational transferability by confining the re-expansion changes to nearby (*e.g.*, first-neighbor and second-neighbor) atoms. These local changes will be designed to preserve the local net charge, local dipole moment vector, and local traceless quadrupole moment tensor.

(c) The re-expansions will minimize some well-chosen loss functions that are convex (*i.e.*, have a unique minimum) and that are computationally easy to solve.

(d) The re-expansions will optimize the assigned QDR atomic charge values to approximately equal chemically-relevant stockholder-partitioned (*e.g.*, DDEC6) net atomic charge values.

(e) More often than not, the re-expansions will reduce the average magnitudes of atom-in-material quadrupole and dipole moments. This design criteria intends to maximize the accuracy of the associated atom-centered point-charge model for approximately reproducing the electrostatic potential surrounding the material.

**Fig. 1:**
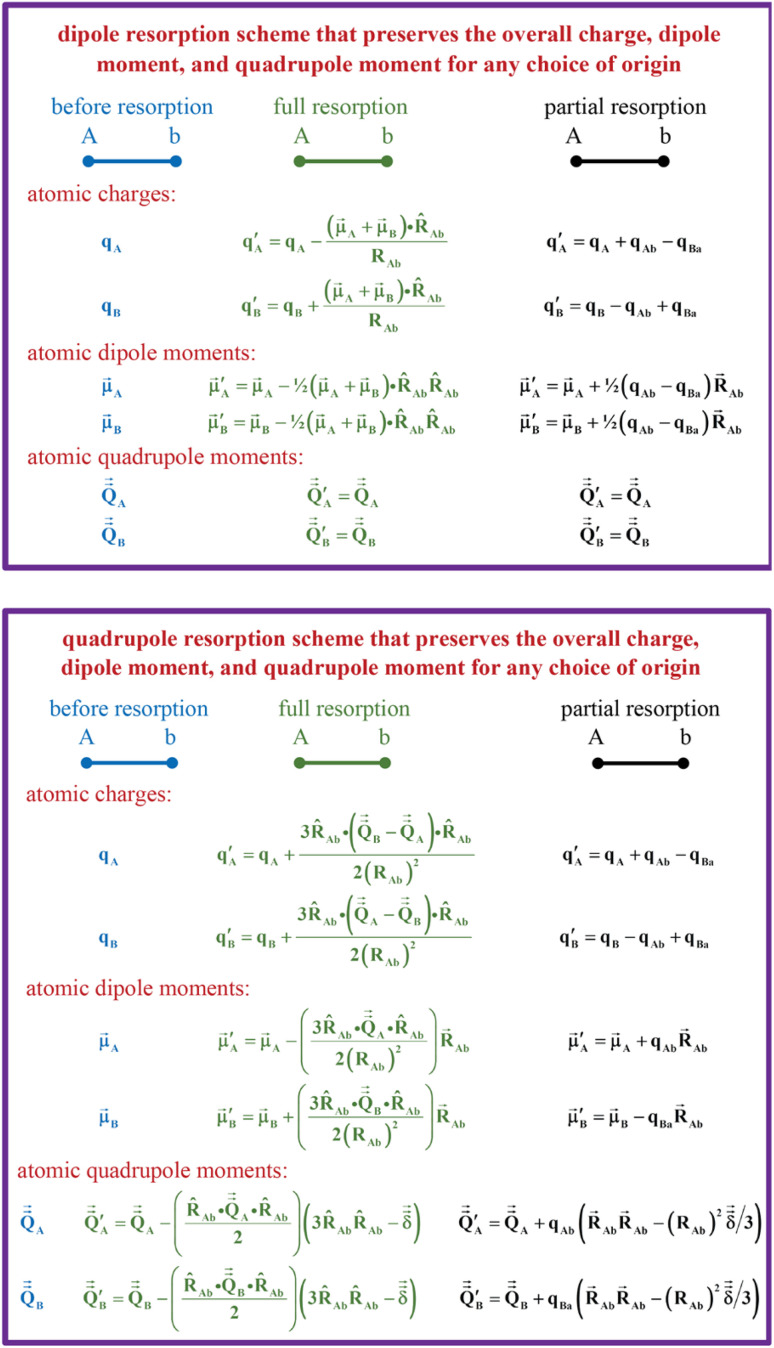
Top panel: A pairwise dipole resorption scheme that preserves the net charge, the total dipole moment, and the total traceless quadrupole moment expanded about any specific origin. In this panel, ‘full resorption’ means the projection of 
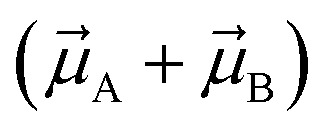
 onto the Ab bond direction, *R̂*_Ab_, is completely resorbed onto the atom-centered charges 
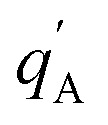
 and 
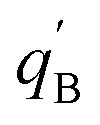
 of the two atoms. General formulas applying to any *q*_Ab_ and *q*_Ba_ values are listed in the ‘partial resorption’ column. Bottom panel: A pairwise quadrupole resorption scheme that preserves the net charge, the total dipole moment, and the total traceless quadrupole moment expanded about any specific origin. In this panel, ‘full resorption’ means the projections of 
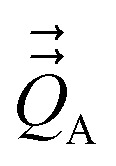
 and 
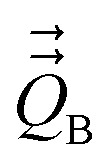
 onto the Ab bond direction, *R̂*_Ab_, are completely resorbed onto the atom-centered charges and atom-centered dipole moments of the two atoms: 
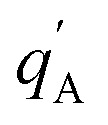
, 
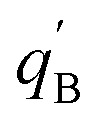
, 
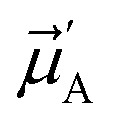
, and 
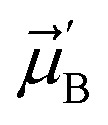
. General formulas applying to any *q*_Ab_ and *q*_Ba_ values are listed in the ‘partial resorption’ column.

Fig. 1 illustrates pairwise atom-in-material dipole and quadrupole moment resorption between an atom A and a nearby atom image b. The illustrated scheme always preserves the net charge, the total dipole moment, and the overall traceless quadrupole moment expanded about any choice of origin. The overall traceless quadrupole moment expanded about site 1 (*e.g.*, atom A), the overall traceless quadrupole moment expanded about site 2 (*e.g.*, image b), and the overall traceless quadrupole moment expanded about the Ab midpoint have the same values after resorption as before resorption. As described in the following subsections, this pairwise resorption scheme can be readily extended to systems containing many atoms. Since changes are performed within pairwise adjacent atoms, such an approach is easy to implement and localizes the resorption changes.

#### 2.2.3 Loss function and linear equation system for resorbing atom-in-material dipole moments

In the dipole resorption (DR) charge scheme, electric charge is moved between two atoms such that the charge *q*_Ab_ is moved from image b to atom A. This adjusts the atomic charge of atom A by *q*_Ab_ and that of atom image b by −*q*_Ab_. This process does not change the system's net charge. Following DR, the ‘new’ atomic charge for atom A is18
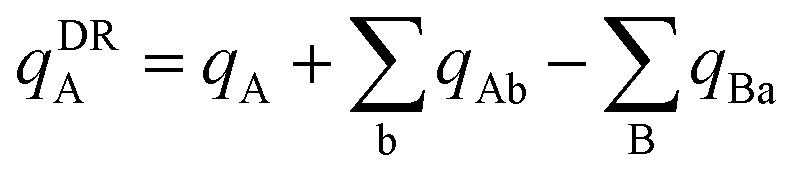
The charge shift *q*_Ab_ generates a dipole moment of19
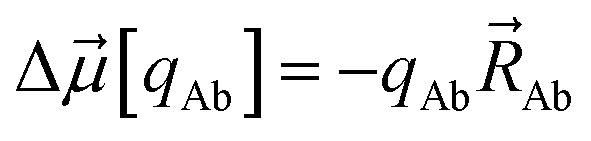
To preserve the quadrupole moment value, half of 
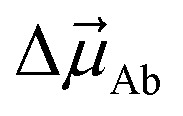
 is assigned atom A while the other half is assigned to atom image b (see Fig. 1). Accordingly, the ‘new’ atomic dipole vector 
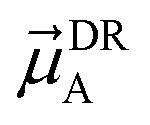
 for atom A satisfies the relationship20
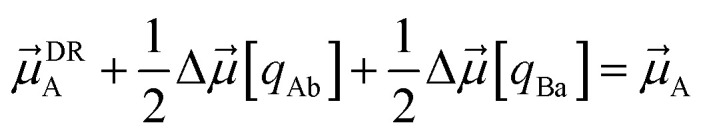
Substituting eqn (19) into (20) and rearranging gives:21
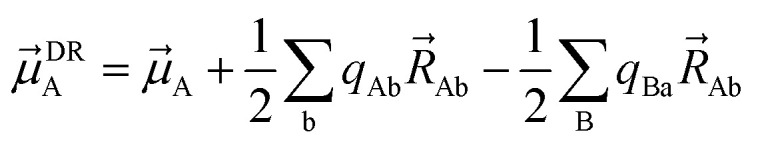


During DR, the traceless atomic quadrupole moment tensor 
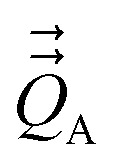
 is left unchanged.

The optimal solution is defined by minimizing the following loss function:22
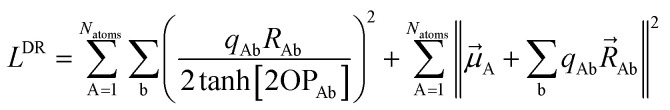
where *N*_atoms_ is the number of atoms in the reference unit cell. The first term in eqn (22) ensures that *q*_Ab_ → 0 as OP_Ab_ → 0; this turns off *q*_Ab_ for atoms that are far away from each other. The second term in eqn (22) minimizes the squared magnitude of the unresorbed part of 
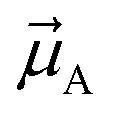
:23
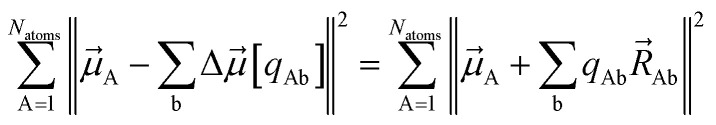


The first term in eqn (22) was derived as follows. To minimize the sensitivity of the DR charges to conformational changes of a material and also to minimize the deviations of DR charges from the parent (stockholder) charges, the loss function should contain a first term of the form24
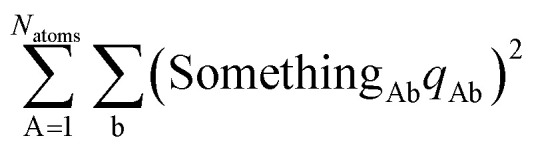
To make the unit dimensions of the first and second terms of the loss function compatible, Something_AB_ must have units of length. For a diatomic molecule AB, using eqn (24) and (23) as the first and second terms in a loss function gives the optimal solution25
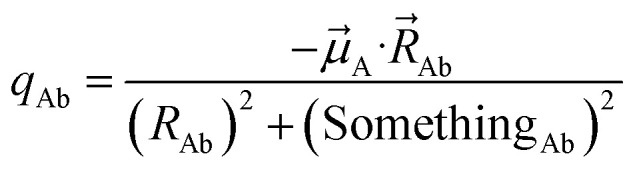
Since Something_Ab_ must have units of length, it is most convenient to define it as proportional to *R*_Ab_, so that *R*_Ab_ can be factored out of the sum in the denominator of eqn (25). Something_Ab_ should be defined to effectively localize the DR correction to 1st-neighbor atoms that directly share a chemical bond. This can be accomplished by setting26
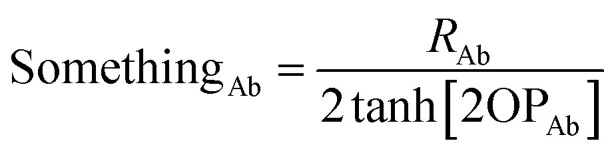
When OP_Ab_ = ½ the bond order (BO_Ab_) computed *via* Manz's comprehensive bond order equation^38^ is significantly larger than ½ and significantly smaller than 1. Examining eqn (25) and (26), the fraction of 
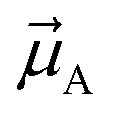
 recovered for a heterodiatomic molecule in the absence of externally-applied fields 0.699 = 69.9% for OP_Ab_ = 0.5, 0.788 = 78.8% for OP_Ab_ = 1, 0.135 = 13.5% for OP_Ab_ = 0.1, 0.0016 = 0.16% for OP_Ab_ = 0.01, 1.6 × 10^−5^ = 1.6 × 10^−3^% for OP_Ab_ = 0.001, *etc.*

The partial derivative is27
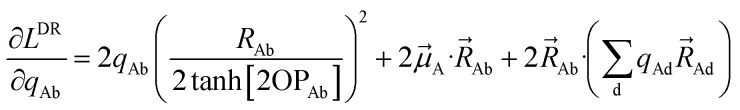


The minimum of the loss function occurs when28
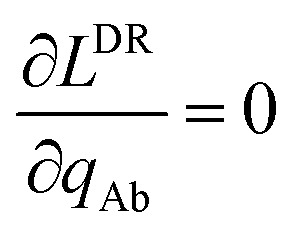


Combining eqn (27) and (28) gives the linear equation system:

29 *MY* = *T*

*T* is a column vector containing the elements30
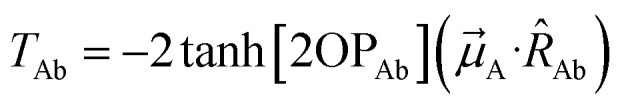
*Y* is a column vector containing the elements31
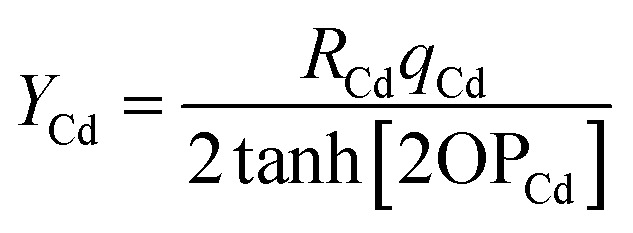
*M* is a symmetric matrix containing the diagonal elements

32 *M*_Ab,Ab_ = 1 + (2 tanh[2OP_Ab_])^2^

and the off-diagonal elements

33 *M*_Ab,Cd≠Ab_ = *δ*_A,C_(2 tanh[2OP_Ab_])(2 tanh[2OP_Cd_])(*R̂*_Ab_·*R̂*_Cd_)

*δ*_A,C_ is the Kronecker delta:34
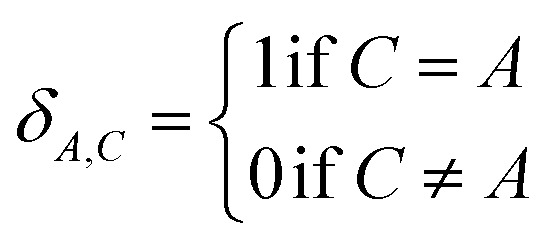


In a periodic material, the atom images b and b′ refer to two different translations of the parent atom B, and this gives rise to the distinct pairs Ab and Ab′, respectively, which occupy different elements (locations) in *T*, *Y*, and *M*. Hence, *M*_Ab,Ab′_ is an off-diagonal element governed by eqn (33).

In eqn (22), the summation occurs over all images b that are within the sum of density cutoff radii for the two atoms (*i.e.*, 10 Å = *r*^cutoff^_A_ + *r*^cutoff^_B_, which was used in this work) or that have OP_Ab_ greater than a threshold (*e.g.*, 10^−4^) (atom image pairs having *R*_Ab_ > *r*^cutoff^_A_ + *r*^cutoff^_B_ have OP_Ab_ = 0 (ref. 37 and 38)). The computed results are not sensitive to the precise value of this threshold. For extremely small OP_Ab_, *q*_Ab_ becomes proportional to (2 tanh[2OP_Ab_])^2^, and this makes (*q*_Ab_*R*_Ab_)^2^/(2 tanh[2OP_Ab_])^2^ also proportional to (2 tanh[2OP_Ab_])^2^. Thus, we are simply omitting from the sum terms that are extremely close to zero when OP_Ab_ < threshold. If a nontrivially translated image a′ of atom A has an overlap population with its parent atom in the reference unit cell greater than or equal to the cutoff threshold (*i.e.*, OP_Aa′_ ≥ threshold) or is within the cutoff distance (*i.e.*, 10 Å), then this pair appears within the sum of eqn (22). This same set of atom image pairs is used to construct the vectors *T* and *Y* and the matrix *M* in eqn (30)–(32).

Because of the *δ*_A,C_ term in eqn (33), the matrix *M* has a block diagonal structure. Let *M*^(A)^, *T*^(A)^, and *Y*^(A)^ represent the blocks of matrix *M* and sections of vectors *T* and *Y* corresponding to atom A:

35 *M*^(A)^_b,d_ = *M*_Ab,Ad_

36 *Y*^(A)^_d_ = *Y*_Ad_

37 *T*^(A)^_b_ = *T*_Ab_

Using this block structure, the loss function defined by eqn (22) can be rewritten in matrix form as38
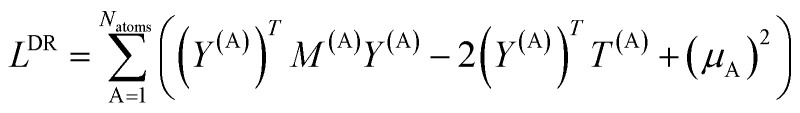
where superscript *T* denotes the vector's transpose, and39
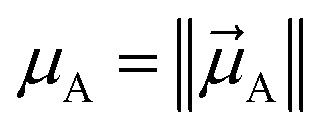


The loss function's variational first derivative expands as40
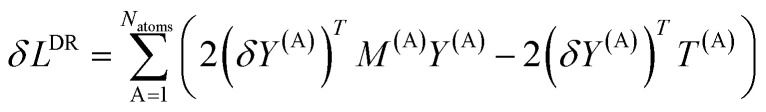
Collecting terms, clearly the solution *δL*^DR^ = 0 is reached when the following linear equation system41
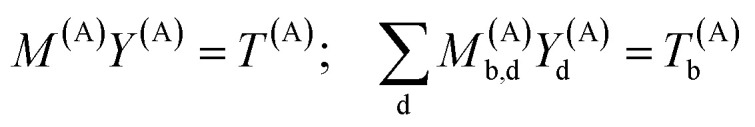
is satisfied for all atoms A in the reference unit cell, where the summation runs over all atom images that have OP_Ad_ > threshold or are within the cutoff radius sum as described above.

The loss function's variational second derivative expands as42
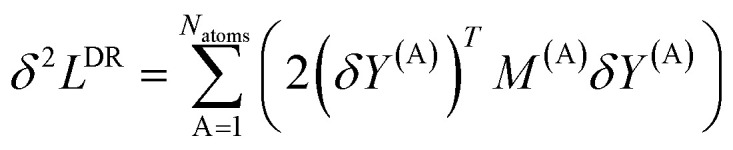
Using the definition of matrix *M* in eqn (32), (33), and (35), this can be rewritten as43
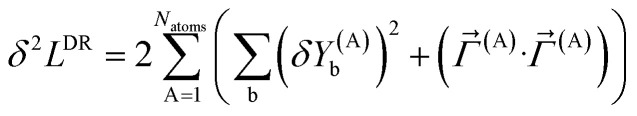
where the vector 
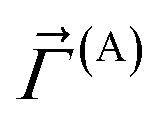
 is defined as44
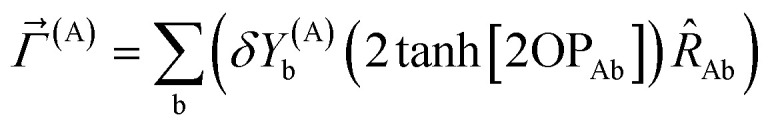


The dot product of a vector with itself is non-negative:45
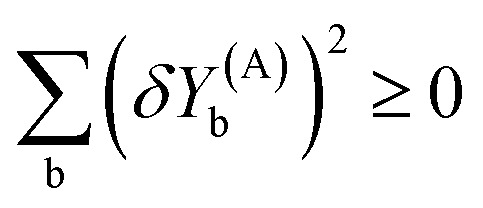
46
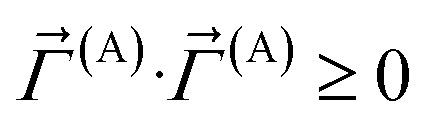
Therefore, it directly follows from eqn (43) that for any nonzero variation of the independent variables, *δY*^(A)^_b_ ≠ 0, *δ*^2^*L*^DR^ is positive definite:

47 *δ*^2^*L*^DR^ > 0

Accordingly, the loss function *L*^DR^ is convex, has positive curvature, and has a unique minimum.

ESI Section S2† proves that *M*^(A)^ has at most four distinct eigenvalues {*λ*_*i*_}. All of these eigenvalues are within the range

48 1 ≤ *λ*_min_ ≤ *λ*_*i*_ ≤ *λ*_max_ ≤ (1 + 16(SOP_A_)^2^)

As shown in eqn (15), SOP_A_ can never be a huge number. This proves matrix *M*^(A)^ can never have a huge condition number (*i.e.*, it is always well-conditioned). Since the matrix *M*^(A)^ is always well-conditioned, this means the optimization landscape is never close to flat. This means that small changes in the inputs, such as small changes in the geometric configuration's internal coordinate values (*i.e.*, bond lengths, bond angle values, and dihedral values) do not cause huge changes in the optimized {*q*_Ab_} values.

#### 2.2.4 Loss function and linear equation system for resorbing atom-in-material quadrupole moments

Quadrupole resorption (QR) proceeds in a manner partly analogous to the dipole resorption. In the QR charge scheme, electric charge is moved between two atoms such that the charge *q*_Ab_ is moved from image b to atom A. This adjusts the atomic charge of atom A by *q*_Ab_ and that of atom image b by −*q*_Ab_. This process does not change the system's net charge. Following QR, the ‘new’ atomic charge for atom A is49
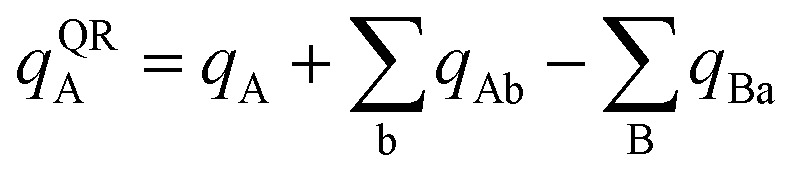


This charge shift *q*_Ab_ generates a dipole moment following eqn (19). As shown in Fig. 1, to preserve the net dipole moment a compensating atomic dipole is placed on atom A50
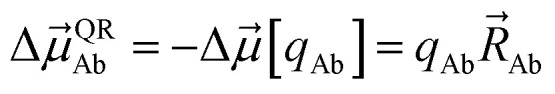
Following QR, the ‘new’ atomic dipole moment for atom A is51
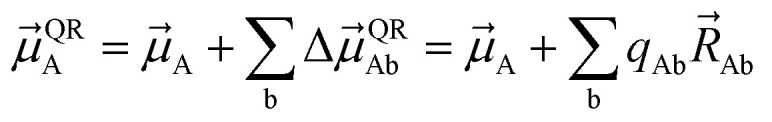


To preserve the overall quadrupole moment value computed about atom A's center, the ‘new’ atomic quadrupole moment for atom A equals its original atomic quadrupole moment minus the quadrupole moment generated by the charge shift:52
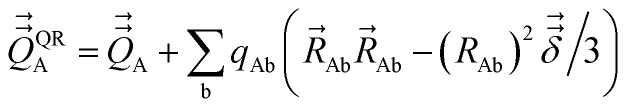
Since the dipole moment 
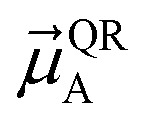
 was placed on atom A's center leaving the atomic dipole on atom image b unchanged, the changed atomic dipoles do not affect the 
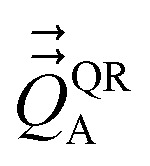
 value.

The optimal solution is defined by minimizing the following loss function:53
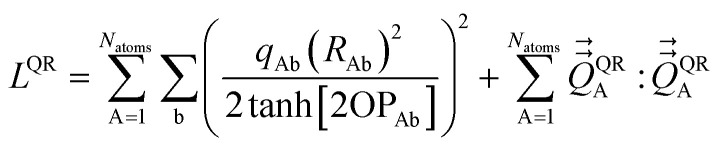
where *N*_atoms_ is the number of atoms in the reference unit cell. The first term in eqn (53) ensures that *q*_Ab_ → 0 as OP_Ab_ → 0; this turns off *q*_Ab_ for atoms that are far away from each other. The first term of *L*^QR^ is constructed analogously to the first term of *L*^DR^ and for similar reasons, except that *L*^QR^ has an additional factor of (*R*_Ab_)^2^ compared to *L*^DR^ to make the units of the first and second terms match in each loss function. The second term in eqn (53) uses the double-dot product to minimize the squared magnitude of the unresorbed part of 
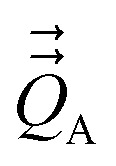
:54
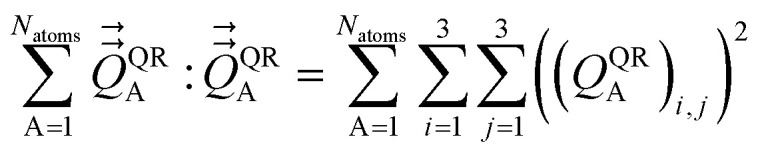


Define55
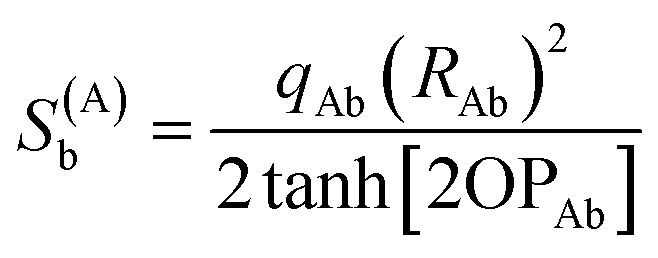
This loss function is manifestly convex with positive definite curvature, because56
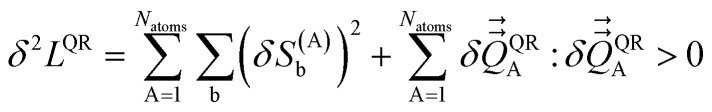
for any nonzero variation in the independent variables, *δS*^(A)^_b_ ≠ 0:57
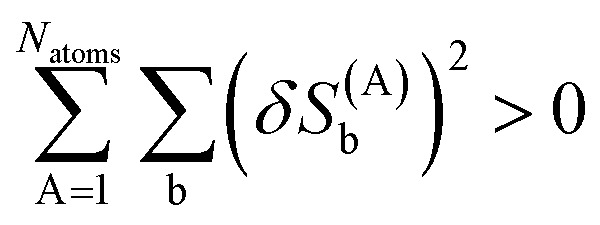
58
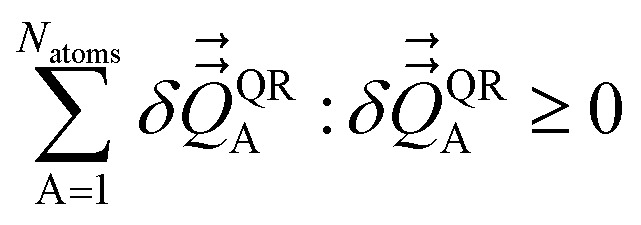
Hence, *L*^QR^ has a unique minimum.

The partial derivative is59
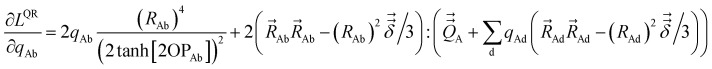


The minimum of the loss function occurs when60
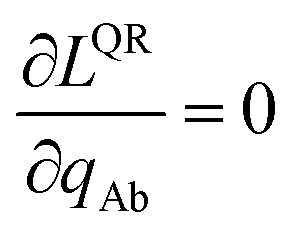
Substituting eqn (59) into (60) and rearranging gives61
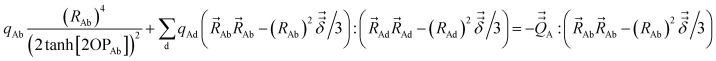
Eqn (61) can be re-written as62
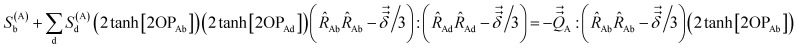


Define63
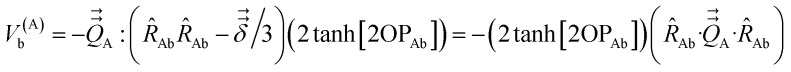
where use has been made of the fact that 
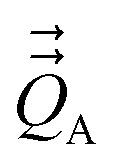
 is traceless. Then eqn (62) is the linear equation system

64 *C*^(A)^*S*^(A)^ = *V*^(A)^

having a symmetric positive definite coefficients matrix *C*^(A)^. The diagonal elements of *C*^(A)^ are

65 *C*^(A)^_b,b_ = 1 + (2/3)(2 tanh[2OP_Ab_])^2^

The off-diagonal elements are66
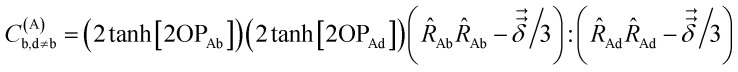
which simplifies to

67 *C*^(A)^_b,d≠b_ = (2 tanh[2OP_Ab_])(2 tanh[2OP_Ad_])((*R̂*_Ab_·*R̂*_Ad_)^2^ − 1/3)

As derived in ESI Section S3,† the matrix *C*^(A)^ has at most eight distinct eigenvalues. All of these eigenvalues are within the range68
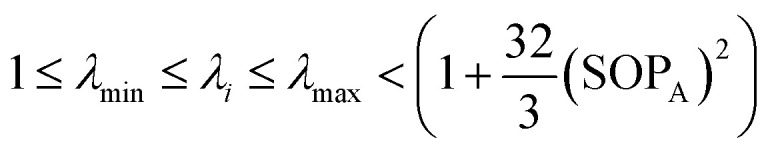
As shown in eqn (15), SOP_A_ can never be a huge number. This proves matrix *C*^(A)^ can never have a huge condition number (*i.e.*, it is always well-conditioned). Since the matrix *C*^(A)^ is always well-conditioned, this means the optimization landscape is never close to flat. This means that small changes in the inputs, such as small changes in the geometric configuration's internal coordinate values (*i.e.*, bond lengths, bond angle values, and dihedral values) do not cause huge changes in the optimized {*q*_Ab_} values.

#### 2.2.5 Two-pass scheme that maximizes the electrostatic potential accuracy and conformational transferability

Table 1 summarizes the approximate spatial extent of changes in atom-in-material charges, dipole, and quadrupole moments. This approximated spatial extent is based on the observation that the OP_Ab_ values are substantial between first-neighbors (*i.e.*, atoms directly sharing a chemical bond) but small between second-neighbors. Some notable features include the following.

**Table 1:** The approximate spatial extent of changes in atom-in-material charges, dipole moments, and quadrupole moments

	QR	DR	QDR	2-Pass QDR	3-Pass QDR
Atomic quadrupoles	Self	No changes	Self	Self	Self
Atomic dipoles	Self	Self, 1st-neighbors	Self, 1st-neighbors	Self, 1st- & 2nd-neighbors	Self, 1st-, 2nd-, and 3rd-neighbors
Atomic charges	Self, 1st-neighbors	Self, 1st-neighbors	Self, 1st-neighbors	Self, 1st- & 2nd-neighbors	Self, 1st-, 2nd-, and 3rd-neighbors

During QR, the atomic quadrupole moments are adjusted only on the self atom; changes in atomic quadrupoles do not propagate to any of the neighboring atoms. No changes in atomic quadrupole moments occur during DR. Accordingly, any possible combinations of QR and DR repeated any number of times will only adjust the atomic quadrupole moments on the self atoms.

QR affects the atomic dipole moment only on the self atom, while DR affects the atomic dipole moment on the self atom and atoms having significant OP_Ab_, which mostly corresponds to the ‘1st-neighbors’. QR and DR each affect the atomic charge values on the self atom and atoms having significant OP_Ab_, which mostly corresponds to the ‘1st-neighbors’.

Since QR changes the atomic dipole values but DR does not affect the atomic quadrupole values, it is strongly preferable to perform DR after QR. This QDR ordering allows the atomic dipole changes generated by QR to be partly resorbed during DR. Consider the hypothetical situation in which atom A and image b have zero-valued atomic dipoles but non-zero-valued atomic quadrupoles. If DR is performed first followed by QR, then DR does nothing in this case, but we still end up with significant atomic dipoles from the QR. If QR is performed first followed by DR, then QR generates some atomic dipole changes, and these atomic dipole changes can be partly resorbed into the atomic charge values during DR.

Since the resorbed portion of the atomic quadrupole is wholly assigned to the self atom (see Fig. 1 and eqn (52)) and not shared with any other atoms, a single pass of QR normally dramatically reduces (and almost eliminates) the magnitudes of the atomic quadrupole moments. In stark contrast, the resorbed portion of the atomic dipole is evenly split between the self atom and the adjacent atom image (see Fig. 1 and eqn (21)). More often than not, this results in the average magnitude of the atomic dipoles decreasing but still leaves substantial atomic dipole moments on some atoms in the material. Another round of DR is usually beneficial to further reduce the average atomic dipole magnitudes. Since QDR does not propagate changes any further than DR does, it is generally desirable to perform a second QDR pass instead of just a second DR pass. As shown in Table 1, a portion of the atomic dipole moment changes that propagated to the 1st-neighbors during the first QDR pass can now propagate to the 2nd-neighbors during the second QDR pass. If a third QDR pass is performed, a portion of these dipole moment changes could propagate to the 3rd-neighbor atoms.

2-Pass QDR means specifically that we begin with the stockholder (*e.g.*, DDEC6) atom-in-material charges, dipole moments, quadrupole moments, and overlap populations. First, we perform QR using these stockholder-computed inputs. Then, we use the atomic charges, dipole moments, and quadrupole moments resulting from this first QR as the inputs to perform DR. Then, we use the atomic charges, dipole moments, and quadrupole moments resulting from this DR as the inputs to perform QR (for a second time). Then, we use the atomic charges, dipole moments, and quadrupole moments resulting from this second QR as the inputs to perform DR (for a second time). Results of this second DR are the final results.

When resorbing atomic dipoles and quadrupoles, changes in the atomic charge values primarily (almost exclusively) propagate to self atoms and 1st-neighbor atoms during each QDR pass. Consequently, n subsequent passes of QDR allows the atomic charge changes to significantly propagate up to *n*th-neighbors.

Imagine that various configurations of a polymer molecule are sampled using molecular dynamics or Monte Carlo simulations in the *NVT* ensemble (aka canonical ensemble). In flexible materials, the dihedral values of rotatable dihedrals are typically less stiff than the bond angles, which are typically less stiff than the bond lengths. During the *NVT* ensemble simulation, changes in bond lengths and changes in bond angle values are typically less dramatic than changes in dihedral values. Also, the bond lengths and bond angles are typically similar amongst various different conformers of a typical biomolecule (*e.g.*, a protein molecule), while some of the dihedral values are dramatically different. Now, it can be inferred that exactly two QDR passes are preferred to maximize the accuracy and conformational transferability of the associated atom-centered point-charge model for constructing a flexible forcefield. Since a bond angle involves only 1st- and 2nd-neighbor atoms, keeping the bond lengths and bond angles approximately (but not necessarily strictly) constant across conformations means 2-pass QDR has excellent conformational transferability (in this work, the term ‘dihedral’ means a proper dihedral not an improper dihedral). Dihedral values for rotatable dihedrals typically change dramatically across different conformations of a flexible material (such as a polymer chain), and dihedrals involve 1st-, 2nd-, and 3rd-neighbor atoms. Since results of 3-pass QDR depend significantly on the 3rd-neighbor atoms, it is more sensitive to the particular dihedral values than 2-pass QDR. In other words, 3-pass QDR (which affects up to 3rd-neighbor atomic charge and atomic dipole values) has significantly higher conformational sensitivity of the resulting atomic charge and atomic dipole moment values than 2-pass QDR. To parameterize a flexible forcefield, the atom-in-material charge values used should be representative across various energetically accessible conformations. Hence, it is preferred to use the 2-pass QDR charge values that are not too sensitive to the particular dihedral values.

In this work, the QDR-DDEC6 results correspond to 2-pass QDR that used the DDEC6 stockholder-partitioned inputs.

### 2.3 Choice of stockholder-partitioning method to use with the QDR procedure

Which stockholder partitioning method is best suited for use with QDR? Within this article, the three stockholder electron density partitioning methods we studied are the DDEC6, Hirshfeld, and MBIS methods.

What defines the close-to-optimal root-mean-squared (rms) charge transfer magnitude? Since net atomic charge is directly experimentally measurable only when the electron density overlap with adjacent atoms is small (*e.g.*, isolated atoms and the nitrogen atom in the N@C_60_ system^36^), for most systems net atomic charge is not directly experimentally measurable. In spite of this, the close-to-optimal rms charge transfer magnitude is objectively quantifiable. Within the present context, the close-to-optimal values of the atomic charges (for constructing flexible forcefields) would correspond to something close to those obtained using a multi-frame ESP fitting procedure (such as MF-CHELPG, MF-MK, or MF-RESP) (here, the term ‘close-to-optimal’ means specifically that the optimized atomic charge values should approximately reproduce the electrostatic potential surrounding the material across multiple conformations). As shown in Table 11, the MF-MK gave the smallest median RRMSE for the validation set geometries and also yielded a relatively high summed correlations (to the various charge assignment schemes) of 11.21. This suggests that those atomic charge values are ‘close-to-optimal’.

As shown in Table 11, the three multi-frame ESP fitting methods had rms charge transfer magnitudes of 0.337 (MF-MK), 0.335 (MF-RESP), and 0.356 (MF-CHELPG). The rms charge transfer magnitudes followed the trend: 0.163 (Hirshfeld) < 0.335–0.356 (multi-frame ESP) < 0.388 (DDEC6) < 0.448 (MBIS). There is a small amount of ambiguity in the optimal rms charge transfer magnitude, as demonstrated by the small range of rms charge transfer magnitudes across various different multi-frame ESP fitting methods. However, the important point is that the Hirshfeld method's rms charge transfer magnitude is objectively much smaller than the optimal range, as has been repeatedly pointed in out in prior literature for decades.^30,34,75–77^ Herein, we see that the DDEC6 method's rms charge transfer magnitude is slightly too high (*i.e.*, 9–15% higher than the multi-frame ESP fitting). The MBIS method's rms charge magnitude is substantially higher.

In order to maximize chemical transferability and conformational transferability of the assigned atomic charge values, the QDR method is purposefully designed to make small (not large) adjustments to the stockholder-partitioned atomic charge values. Because QDR makes small adjustments to the atomic charge values, the rms charge transfer magnitude is only modestly affected by the QDR adjustment to the atomic charge values. In other words, a stockholder partitioning method that has substantially too small (*cf.* too large) of a rms charge transfer magnitude before the QDR procedure is expected to still have substantially too small (*cf.* too large) of a rms charge transfer magnitude for the post-QDR atomic charge values. For example, the QDR-DDEC6 method had a rms charge transfer magnitude of 0.379 compared to 0.388 for DDEC6.

The NaCl crystal demonstrates unambiguously why the QDR procedure cannot fully correct the rms charge transfer magnitude if an inaccurate stockholder-partitioning method is used. For the NaCl crystal at ambient pressure (see Table 12), the Na atom charges are 0.21 (Hirshfeld) and 0.85 (DDEC6). Due to symmetry, all atomic dipole and quadrupole moments are zero in the optimized geometry of this crystal. Accordingly, the QDR procedure yields atomic charges that are identical to the starting stockholder-partitioned atomic charges for the optimized geometry of this crystal structure. If a stockholder-partitioning method yields atomic charges that are too small in magnitude (*e.g.*, Hirshfeld) for this crystal structure, the QDR procedure cannot fix that problem. None of the previously published dipole-adjustment^86,92^ or dipole-and-quadrupole-adjustment^93^ schemes could fix that problem, unless the stockholder-partitioning method itself is changed to something that yields the approximately correct charge-transfer magnitude (*e.g.*, DDEC6, ADCHα-I,^16^ or constrained MBIS^94^).

We studied the water molecule as an example to compare the QDR-DDEC6, QDR-Hirshfeld, and QDR-MBIS performance. The atomic charge magnitudes in this molecule followed the trend Hirshfeld < ESP fit < DDEC6 < MBIS, which is the same trend as the rms charge transfer magnitudes for the entire dataset of nonperiodic materials (see Table 11). Tables 2–4 track changes during each step of the QDR procedure. The largest (*cf.* smallest) overall change in atomic charge magnitude occurred for the QDR-HD (*cf.* QDR-DDEC6) procedure. This reflects the fact that the starting Hirshfeld atomic charges were farthest from optimal, while the starting DDEC6 atomic charges were closer to optimal. After the QDR procedure, results from the QDR-HD, QDR-DDEC6, and QDR-MBIS were somewhat mixed, because different methods performed better on different metrics.

**Table 2:** Tracking the progress of various quantities during the 2-pass QDR-DDEC6 procedure applied to a water molecule. The quadrupole moments are with respect to the center of nuclear charge. For the molecular 
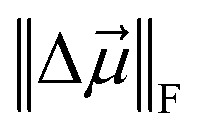
, molecular 
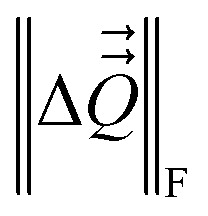
, RMSE, and RRMSE, the quantities outside parentheses are for the point-charge model, while the quantities inside parentheses are for the model including atomic dipoles

	H atom *q*	H atom 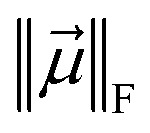	H atom 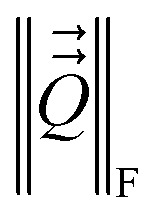	O atom *q*	O atom 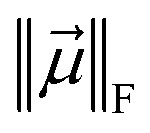	O atom 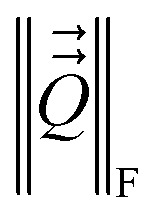	Molecular 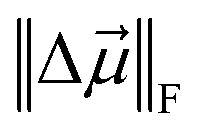	Molecular 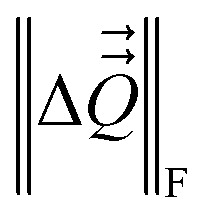	Electrostatic RMSE (kcal mol^−1^ e^−1^)	Electrostatic RRMSE
Starting	0.388	0.042	0.053	−0.776	0.136	0.384	0.13 (0.00)	0.66 (0.49)	1.12 (0.48)	0.22 (0.09)
After QR #1	0.446	0.056	0.041	−0.892	0.283	0.186	0.26 (0.00)	0.48 (0.26)	1.85 (0.29)	0.36 (0.06)
After DR #1	0.381	0.112	0.041	−0.762	0.212	0.186	0.12 (0.00)	0.68 (0.26)	1.05 (0.31)	0.20 (0.06)
After QR #2	0.411	0.117	0.039	−0.822	0.283	0.091	0.18 (0.00)	0.59 (0.16)	1.38 (0.26)	0.27 (0.05)
After DR #2	0.372	0.152	0.039	−0.744	0.240	0.091	0.09 (0.00)	0.70 (0.16)	0.96 (0.28)	0.19 (0.05)

**Table 3:** Tracking the progress of various quantities during the 2-pass QDR-HD procedure applied to a water molecule. The quadrupole moments are with respect to the center of nuclear charge. For the molecular 
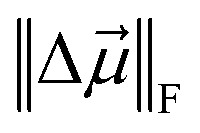
, molecular 
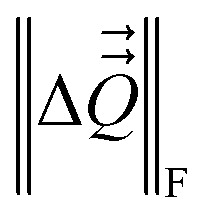
, RMSE, and RRMSE, the quantities outside parentheses are for the point-charge model, while the quantities inside parentheses are for the model including atomic dipoles

	H atom *q*	H atom 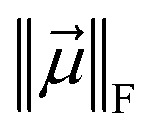	H atom 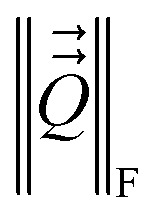	O atom *q*	O atom 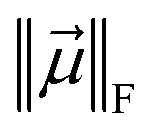	O atom 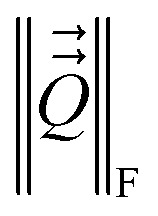	Molecular 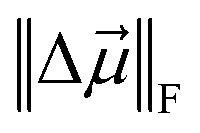	Molecular 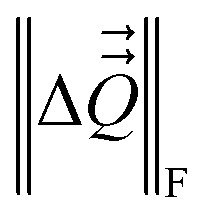	Electrostatic RMSE (kcal mol^−1^ e^−1^)	Electrostatic RRMSE
Starting	0.147	0.225	0.112	−0.294	0.145	0.417	0.40 (0.00)	1.37 (0.62)	2.98 (0.63)	0.58 (0.12)
After QR #1	0.198	0.230	0.111	−0.396	0.025	0.258	0.29 (0.00)	1.22 (0.45)	2.23 (0.49)	0.43 (0.10)
After DR #1	0.282	0.154	0.111	−0.564	0.068	0.258	0.10 (0.00)	0.97 (0.45)	1.14 (0.47)	0.22 (0.09)
After QR #2	0.314	0.157	0.110	−0.628	0.141	0.164	0.03 (0.00)	0.88 (0.34)	0.87 (0.39)	0.17 (0.08)
After DR #2	0.332	0.140	0.110	−0.664	0.161	0.164	0.01 (0.00)	0.82 (0.34)	0.80 (0.39)	0.15 (0.08)

**Table 4:** Tracking the progress of various quantities during the 2-pass QDR-MBIS procedure applied to a water molecule. The quadrupole moments are with respect to the center of nuclear charge. For the molecular 
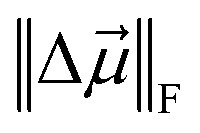
, molecular 
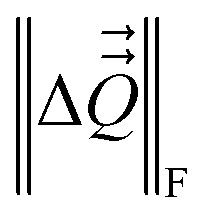
, RMSE, and RRMSE, the quantities outside parentheses are for the point-charge model, while the quantities inside parentheses are for the model including atomic dipoles

	H atom *q*	H atom 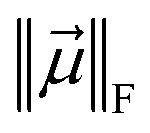	H atom 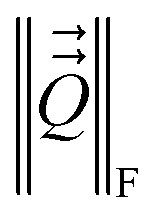	O atom *q*	O atom 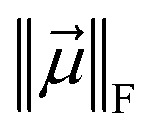	O atom 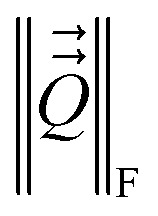	Molecular 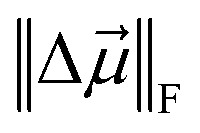	Molecular 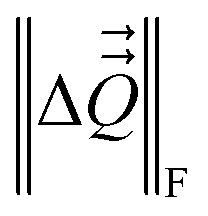	Electrostatic RMSE (kcal mol^−1^ e^−1^)	Electrostatic RRMSE
Starting	0.433	0.029	0.019	−0.866	0.224	0.371	0.23 (0.00)	0.52 (0.41)	1.67 (0.41)	0.32 (0.08)
After QR #1	0.468	0.030	0.019	−0.936	0.303	0.265	0.31 (0.00)	0.42 (0.30)	2.15 (0.31)	0.42 (0.06)
After DR #1	0.416	0.073	0.019	−0.832	0.245	0.265	0.19 (0.00)	0.58 (0.30)	1.45 (0.33)	0.28 (0.06)
After QR #2	0.441	0.073	0.018	−0.882	0.302	0.190	0.25 (0.00)	0.50 (0.22)	1.78 (0.27)	0.35 (0.05)
After DR #2	0.403	0.108	0.018	−0.806	0.259	0.190	0.16 (0.00)	0.62 (0.22)	1.45 (0.33)	0.28 (0.06)

All norms in Tables 2–4 are the Frobenius norm, 
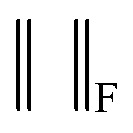
 (aka Euclidean norm). The molecular 
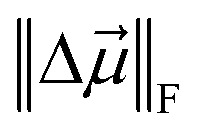
 and molecular 
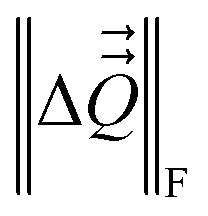
 are defined as the Frobenius norm of the difference between the point-charges (or point-charges plus atomic dipoles) model and the reference QM-computed molecular multipole moment. For individual steps of the QDR procedure, the most consistent trends were: (i) reduction in the Frobenius norm of the atomic quadrupole moments during quadrupole resorption steps, (ii) reduction in molecular 
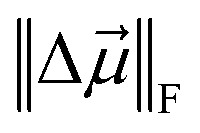
 during dipole resorption steps, (iii) reduction in molecular 
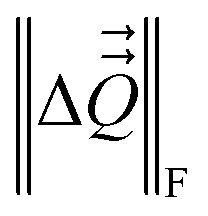
 during quadrupole resorption steps, (iv) no changes in the molecular 
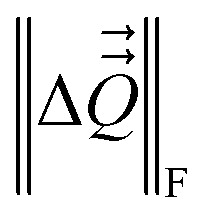
 predicted by the point-charges plus atomic dipoles model during dipole resorption steps, and (v) no changes in the atomic quadrupoles during dipole resorption steps. There is a general, albeit not necessarily monotonic, improvement in the electrostatic RMSE and RRMSE across the QDR steps.

Following the above arguments, it is important to select a stockholder-partitioning method that has an approximately close-to-optimal rms charge transfer magnitude. Amongst the three stockholder partitioning methods studied in this work, the DDEC6 method currently comes closest to this ideal, although its rms charge transfer magnitude is slightly higher than optimal. Another important consideration is to select a stockholder-partitioning method that yields relatively small rms atomic dipole and quadrupole magnitudes. This helps keep the QDR adjustments small, which should in turn help preserve the chemical and conformational transferability of the assigned QDR charges. As shown in Table 8, the DDEC6 method yields slightly smaller rms atomic dipole and quadrupole magnitudes than the Hirshfeld and MBIS methods. For these reasons, we used the QDR procedure with DDEC6 stockholder partitioning (*i.e.*, QDR-DDEC6) throughout the remainder of this work.

### 2.4 Linear-scaling computational costs with good parallelization efficiency

The symmetric matrices *M*^(A)^ and *C*^(A)^ are positive definite. As shown in eqn (48) and (68), none of their eigenvalues are close to zero. This ensures that their inverses, (*M*^(A)^)^−1^ and (*C*^(A)^)^−1^, always exists and are not close to singular. The exact solution could be found by first computing the inverse matrices and then computing *Y*^(A)^ = (*M*^(A)^)^−1^*T*^(A)^ and *S*^(A)^ = (*C*^(A)^)^−1^*V*^(A)^.

However, the exact solution is computed more efficiently using our method described in ESI Section S4.† For dipole resorption, our method requires a maximum of four matrix-vector multiplies (*i.e.*, up to four multiplications of *M*^(A)^ times a column vector) and does not require explicit computation of (*M*^(A)^)^−1^. For quadrupole resorption, our method requires a maximum of eight matrix-vector multiplies (*i.e.*, up to eight multiplications of *C*^(A)^ times a column vector) and does not require explicit computation of (*C*^(A)^)^−1^. Our computational method is based on the conjugate gradient^95,96^ method but makes the further computational efficiency improvement that the matrices *M*^(A)^ and *C*^(A)^ do not have to be explicitly allocated in memory. Our method is easy to parallelize using shared-memory (*e.g.*, OpenMP^20,37,97,98^) and makes efficient use of cache without requiring excessive memory allocations.

**Fig. 2:**
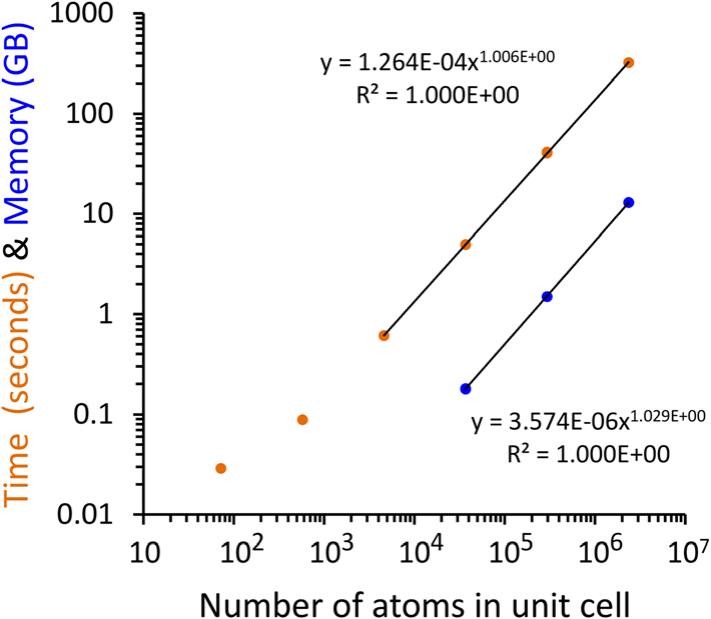
Plot of the computational time and random access memory (RAM) required to compute the quadrupole-dipole-resorbed (QDR) charges for unit cells containing different numbers of atoms. Results are for serial program execution using one computing core. The required computational time and memory include all steps from program start to program finish: input file reading, solving the linear equation systems, and output file writing. The required computational time and memory scale linearly with increasing number of atoms in the material's unit cell.

As a computational test, different sized periodic unit cells were prepared for the ALPO-5 crystal. Specifically, periodic unit cells were prepared containing 72, 576, 4608, 36 864, 294 912, and 2 359 296 atoms. No quantum chemistry calculations were performed on the larger unit cells; rather, the DDEC6 atomic population analysis files (which are the input files for QDR charge computation) were prepared by constructing larger units cells from the smaller unit cells. These input files explicitly stored all required input information for each and every individual atom in the larger unit cell; we did not reduce the amount of information stored using symmetry equivalency. The material (and its associated computational model) does not ‘end’ at the unit cell's boundary, because the ALPO-5 crystal has 3-dimensional periodic boundary conditions. Fig. 2 plots the required computational time and random access memory (RAM) for serial program execution to compute the QDR charges from the input DDEC6 net atomic charges, atom-in-material dipole moments, atom-in-material quadrupole moments, and overlap populations. Each computational time displayed in Fig. 2 and Table 5 is the average of three runs. As clearly shown in Fig. 2, the required computational time and memory scale linearly with increasing number of atoms in the material's unit cell. For the smallest three periodic unit cells (*i.e.*, containing 72, 576, and 4608 atoms), the memory requirements were too small to precisely measure using our computational setup; however, by extrapolating the linear trendline the predicted RAM requirements for these three small periodic unit cells are predicted to be approximately 0.29 (for 72 atoms), 2.5 (for 576 atoms), and 21 MB (for 4608 atoms).

**Table 5:** Parallelization efficiency for solving the linear equation system computing the quadrupole-dipole-resorbed (QDR) charges. The listed computational times start after input file reading and end before output file writing. The listed computational times correspond specifically to the tasks labeled ‘QDR pass #1 and QDR pass #2’ (aka ‘the 2-pass QDR procedure’) in Fig. S1 of the ESI. The system studied contained 2 359 296 atoms in the material's periodic unit cell and had 97 845 248 nonzero overlaps, which gave a total of 4 (QR or DR steps) × 97 845 248 unknowns per step = 391 380 992 unknowns to solve for

Number of cores	Require RAM (GB)	Average time (seconds)	Parallelization efficiency
Serial	13	65.1	100.0%
1	13	67.0	97.2%
2	13	37.4	87.0%
4	13	22.8	71.5%
8	13	14.1	57.8%

For the periodic unit cell containing 2.36 million atoms, Table 5 summarizes the computational times and parallelization efficiencies for the OpenMP parallelized code ran on 1, 2, 4, and 8 computing cores compared to the not-parallelized serial code. For these calculations, the RAM requirements were measured and found to be 13 GB independent of the number of parallel computing cores. As shown in Table 5, the parallelization efficiencies were excellent. By using parallel computation, one can easily solve the linear equation system computing the QDR charges in less than one minute for a material containing a couple million atoms in its unit cell. For the periodic unit cell containing 2.36 million atoms and 97.8 million nonzero overlap population values, the input file reading was not parallelized and took approximately 232 seconds, and the buffered output file writing was not parallelized and took approximately 25 seconds (the input file reading took longer than output file writing, because the data is sorted during input file reading as shown in Fig. S2 of the ESI†). This calculation used real-valued numbers providing at least 15 digits of accuracy (aka ‘double-precision’ reals) and 64-bit integers.

The key purpose of these computational tests was to quantify how the time and memory required to compute the QDR charges scale with increasing number of atoms in the material's unit cell. Using enlarged periodic unit cells of the same material (*e.g.*, ALPO-5 crystal) provides a direct test of this scaling. All of these periodic unit cells converged to functionally equivalent sets of QDR charges, so in practical applications there is no other motivation for creating enlarged unit cells (aka ‘supercells’) that are multiples of the material's smallest unit cell. This was only done here to see how the required computational time and memory scaled with increasing number of atoms in the material's unit cell. These scaling tests showed the QDR charges can be computed from the stockholder net atomic charges, overlap populations, and atom-in-material dipole and quadrupole moments in less than one minute (including the time required for input file reading, all calculation steps, and output file writing) on a single computing core when the material's unit cell contains up to 400 000 atoms. For the unit cell containing 2.36 million atoms, the QDR charges were computed from the stockholder net atomic charges, overlap populations, and atom-in-material dipole and quadrupole moments in 5.4 minutes (including the time required for input file reading, all calculation steps, and output file writing) on a single computing core. The practical significance of this is that the QDR charges for large biomolecules (*e.g.*, proteins, DNA, RNA, *etc.*) containing several hundred thousands or even millions of atoms can be computed in a few minutes or quicker.

## 3. Computational methods

### 3.1 Quantum chemistry methods

To explore the effects of various molecular conformations, we compared the electrostatic models across many different conformations of each material. For both periodic and non-periodic structures, the training set included 21 geometries: the optimized ground state geometry plus 20 other conformations. The validation set included 20 different conformations. No conformation was used in both the training and validation sets.

In all cases, the ‘optimized ground state geometry’ (also called the ‘low energy conformation’) is at least a local ground state (*i.e.*, a local energy minimum) on the material's potential energy surface. For the simpler molecules, this ‘low energy conformation’ is the material's global ground state geometry (*i.e.*, having the globally lowest energy). For molecules having a large number of different conformers, we did not exhaustively determine which conformer had the globally lowest energy, because that is not our study's purpose. Rather, our study's purpose was to investigate how well the atomic charges computed for one local ground state geometry (*i.e.*, one conformer) perform for reproducing the electrostatic potential surrounding the molecule across various geometric conformations.

For both the training and the validation datasets, the same QM level of theory was always used to compute the system's electron density distribution and its QM electrostatic potential. This ensured the atomic charges were computed from the same QM level of theory as the target electrostatic potential.

#### 3.1.1 Nonperiodic systems

For the nonperiodic systems, we performed quantum chemistry calculations using the Gaussian16 program.^89^ All the calculations were performed at the B3LYP^99,100^/def2tzvpd^101^ level of theory with GD3BJ^102–106^ empirical dispersion. The convergence criteria for geometry optimization were as follows: (1) the maximum force was less than 0.00045 hartrees bohr^−1^; (2) the root-mean-squared (RMS) force was less than 0.0003 hartrees bohr^−1^; (3) the maximum displacement was less than 0.0018 bohr; and (4) the RMS displacement was less than 0.0012 bohr.

Conformations were generated using one of several kinds of conformational sampling. First, we divided the materials into two groups depending on whether each material contained any chemical groups with easily rotatable dihedrals. To determine whether a material contained any rotatable dihedrals, we used the specific definition of ‘rotatable dihedral’ introduced in ref. 8. Examples of easily rotatable chemical functional groups include methyl, alkyl chains, hydroxyl, thiol, nitro, phosphate, amino, –CF_3_, *etc.* For each structure without any rotatable dihedrals, we performed AIMD calculations starting from the optimized ground state geometry.

When the structure contained rotatable functional groups, the 20 non-optimized conformations were generated as follows. For the organic molecules with rotatable functional groups, we generated conformers using either: (a) the ETKDG method^107^ in the Research Database Toolkit (RDKit)^108^ or (b) conformers published on the pubchem website. Case # 1: when this generated 40 or more distinct conformations, then we selected 20 at random for the training set and a different 20 for the validation set. Case # 2: when this generated fewer than eight different conformations, then we performed AIMD calculations using the generated conformations plus the optimized structure as the starting geometries for various runs. Case # 3: when this generated between 8 and 39 conformers, then we performed AIMD calculations using a randomly generated subset containing eight different conformers. Case # 4: for the non-organic structures, we generated different conformations by hand as the starting point for each AIMD calculation. These hand-generated structures contained various rotations of the rotatable functional groups.

Because the N@C_60_ and ATP molecules contained relatively large numbers of atoms, AIMD calculations for these two molecules were performed in VASP by placing the corresponding molecule in a large cubic unit cell containing several angstroms of vacuum all around (this lowered the computational cost of the AIMD computations for these two molecules compared to what the computational cost would be for performing BOMD simulations using Gaussian basis set code). The AIMD calculations for these two molecules were performed using the same VASP settings as described in Section 3.1.2 below. The subsequent single-point energy calculations to generate the QM-computed electrostatic potentials and electron densities (used to compute the atomic charges) of the 20 AIMD geometries for the training dataset and 20 AIMD geometries for the validation dataset were performed in Gaussian16 at the B3LYP/def2tzvpd level of theory with GD3BJ empirical dispersion. The ground-state geometry optimization for these two molecules was also performed in Gaussian16 using this same level of theory.

For other non-periodic systems, the AIMD calculations were performed as follows. Four Born–Oppenheimer molecular dynamics (BOMD) simulations were performed in Gaussian16 to generate the configurations for the training dataset, and another four for the validation dataset. Each BOMD simulation was run for 100 trajectory points (steps). The Hessian was calculated analytically every 30 steps. The simulation temperature was 300 K. The dynamic step size was set to 0.0812 amu^1/2^ bohr. Every simulation was run with different random seeds. After this AIMD calculation completed, we extracted every 20th geometry. Then we performed single-point DFT calculations on each of these 20 geometries to generate their electron density distributions and electrostatic potentials.

#### 3.1.2 Periodic systems

For the periodic systems, quantum chemistry calculations were computed using the Vienna *ab initio* simulation package (VASP).^109–112^ All calculations were performed using the PBE^113^ functional with DFT-D3 Becke–Johnson damping function^102–106^ and the projected augmented wave (PAW) method.^114,115^ The energy convergence criterion for the self-consistent field (SCF) cycles was set to 10^−6^ eV. The *k*-point grid was set to ensure that the product of the length of each lattice vector and the number of *k*-points exceeded 16 Å. The planewave energy cut-off was 400 eV. A Prec = Accurate grid with Addgrid = False were used to avoid wrap-around (aka aliasing) errors.

A geometry optimization was performed, allowing relaxation of the atomic positions, cell shape, and volume. The convergence criterion was set such that the absolute magnitude of each force component (*i.e.*, *F*_*x*_, *F*_*y*_, and *F*_*z*_) for every atom was below 0.01 eV Å^−1^ (however, for the BN nanotube array two of the lattice vectors were held fixed at 16.0 Å to ensure the nanotubes remained separated).

Four AIMD runs were performed to generate different conformations for the training dataset. A separate four AIMD runs were performed to generate conformations for the validation dataset. The AIMD simulations encompassed 100 geometry steps per run, originating from the optimized geometry. The forces were computed in response to atomic positional changes, while maintaining constant cell shape and volume. A time step of 1 femtosecond was employed, accompanied by an initial temperature of 300 K, utilizing a microcanonical (NVE) ensemble. This setup used the specific VASP settings: IBRION = 0, NSW = 100, ISIF = 0, MDALGO = 0, POTIM = 1, TEBEG = 300, SMASS = −3. Every 20th geometry was extracted for further analysis. Then we performed single-point DFT calculations on each of these 20 geometries (using VASP keywords LCHARG = True, LAECHG = True, and LVHAR = True) to generate their electron density distributions and electrostatic potentials.

### 3.2 Various charge assignment methods

For nonperiodic materials, we tested the following methods for assigning net atomic charges: ADCH,^86^ CHELPG,^80^ charge model 5 (CM5),^75^ sixth generation density-derived electrostatic and chemical (DDEC6),^32^ QDR-DDEC6, Hirshfeld,^54^ MBIS,^14^ MK,^81,82^ and RESP.^83^ The ADCH, CHELPG, CM5, Hirshfeld, MBIS, MK, and RESP charges were computed with Multiwfn^90,91^ version 3.8. The DDEC6 and QDR-DDEC6 charges were computed using the Chargemol^31,37^ program. For CHELPG, MK, and RESP, the van der Waals radii was the universal forcefield (UFF) radii scaled by 1/1.2 as defined by Multiwfn.^116^ For RESP, a hyperbolic penalty function with two-stage fitting was used as recommended in the article introducing the RESP method: constants were: *a* = 0.0005 (stage 1), *a* = 0.001 (stage 2 on selected atoms), and *b* = 0.1 (both stages).^83^

The Multiwfn program performs MBIS analysis as follows. For electron densities that were generated from QM computations that replaced some core electrons with a relativistic effective core potential (RECP), Multiwfn uses a stored core-electron density library^117^ to add these missing core electrons back in at the start of MBIS partitioning so that an effective all-electron MBIS partitioning is performed.^90^ According to the paper defining the MBIS method, the initial guess for the population (*i.e.*, *N*^initial_guess^_A,*i*_) of each Slater function defining the MBIS pro-atom is “set to the number of electrons in each shell of the corresponding neutral isolated atom”.^14^ Initially, it was unclear to us whether this means the 3d electrons should be initialized into the 3rd or 4th MBIS Slater function of the pro-atom. Because the converged MBIS results may be sensitive to the choice of *N*^initial_guess^_A,*i*_, it is necessary to have some agreed upon initialization protocol.^14^ Multiwfn initializes the MBIS Slater functions such that the (*n* − 1)*d* (if any) and (*n* − 2)*f* (if any) electrons are initialized into the same Slater function as the ns (if any) and np (if any) electrons. For example, Multiwfn initializes the Slater functions for a gold atom with the following populations: 2, 8, 8, 18, 18, 25. We used Multiwfn's default convergence criteria for computing MBIS charges: abs[Δ*q*_A_] < 0.0001 between subsequent charge cycles with a maximum of 500 charge cycles.

For the nonperiodic materials, the multiframe CHELPG (MF-CHELPG), multiframe Merz–Kollman (MF-MK), and multiframe RESP (MF-RESP) were computed in Multiwfn version 3.8 using the same settings as described above. These optimized the charges to simultaneously minimize the electrostatic potential RMSE across all 21 conformations in the training set. A key point is that the grids used to minimize this RMSE are those defining each of these charge methods. For example, CHELPG uses a Cartesian grid of points, while Merz–Kollman uses grid points on Connolly surfaces.^80–82^

For each of the periodic systems, the electron density distributions and local electrostatic potential were generated and saved to files using VASP. These were analyzed in post-processing to compute the following net atomic charges: CM5, DDEC6, QDR-DDEC6, Hirshfeld, REPEAT,^84^ and RESP. The CM5, DDEC6, QDR-DDEC6, and Hirshfeld charges were calculated using the Chargemol program. The RESP and REPEAT charges were calculated using the REPEAT 2.0 program.^84^ The valid grid points for computing the RESP and REPEAT charges were defined as those outside a surface defined by 1.0 times the UFF^84,118^ van der Waals radii. The RESP charges for periodic systems were computed using quadratic constraints of the form *a*(*q*_*i*_)^2^ with the constant *a* = 0.01 for all elements except hydrogen and *a* = 0 (no restraints) for hydrogen atoms as described in the original RESP paper.^83^

Importantly, all of the charge assignment methods used in this work have no explicit basis-set dependence. The ADCH, CM5, DDEC6, QDR-DDEC6, Hirshfeld, and MBIS atomic charges are functionals of the material's QM-computed electron density distribution. The CHELPG, MK, REPEAT, and RESP atomic charges are functionals of the material's QM-computed electrostatic potential. As long as the QM level of theory is adequate to provide a reasonably accurate computation of the material's electron density distribution and its electrostatic potential, these computed atomic charge values have low sensitivity to the specific choice of exchange–correlation functional and basis set. Please see the prior literature for a more detailed discussion and associated computational tests showing this.^30,32,34,119–122^

### 3.3 Method for computing the electrostatic root-mean-squared errors (RMSEs)

To quantify the accuracy of the different charge assignment methods, we conducted tests that measured how accurately each method reproduced the electrostatic potential surrounding the material. This was done using a program^123^ that calculated the root mean squared error (RMSE) and relative RMSE (RRMSE) of the electrostatic potential produced by each tested charge method compared to the benchmark QM-computed electrostatic potential.

The RMSE of each of the charge methods for each system was calculated as follows:69
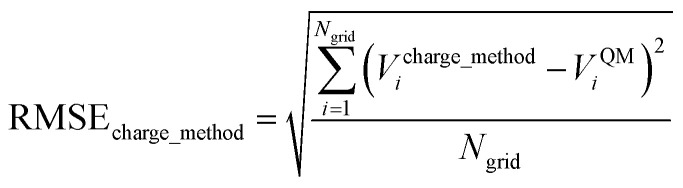
where *V*^charge_method^ is the electrostatic potential produced by a charge assignment method for a particular system, while *V*^QM^ is the quantum-mechanically-computed electrostatic potential for the same system, and *N*_grid_ is the number of valid grid points. The RRMSE was calculated as follows:70
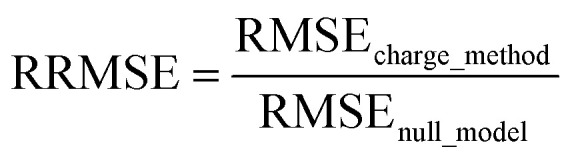
where RMSE_null_model_ represents a model for which the charge of each and every atom in a system is set to zero (*i.e.*, no model).

In eqn (69), the sum over *i* corresponds to the sum over valid grid points within the region of interest. We defined a set of valid grid points in the region immediately outside the material and within its pores. The selection of these grid points was based on three criteria. First, to make sure that the selected points were outside the core-electron density of individual atoms, valid grid points were required to have an electron density less than 10^−4^ e bohr^−3^. Second, to prevent the grid points extending infinitely into space, we implemented an outer radius cutoff of 5 Å, meaning each valid grid point must be within 5 Å of at least one atom in the system. Third, to ensure the potential is not measured inside the electronic density, we set an inner radius cutoff of 2 Å. Thus, a valid grid point in our analysis satisfies all of the following conditions: it has an electron density less than 10^−4^ e bohr^−3^, is farther than 2 Å away from every atom in the system and is within 5 Å of at least one atom in the system.

For nanoporous materials, one could choose the outer cutoff radius for valid grid point selection to be the same as for the nonperiodic (*i.e.*, molecular) materials, or one could choose there to be no outer cutoff radius so that the valid grid points always extend to the center of the nanoporous cavity. Either choice is justifiable. The choice of using no outer cutoff radius can be justified on the basis that it ensures the entire nanoporous cavity is included in the valid grid points for RMSE computation, while the choice of using a 5 Å outer cutoff radius can be justified on the basis that it ensures the valid grid points for RMSE computation are not too far away from atoms (where important adsorption sites occur). For 2-dimensional periodic materials such as graphene, one needs to use a finite outer cutoff radius such as 5 Å for the RMSE computation. As well, one could also use a periodic planewave QM computation to study a single molecule placed in a large periodic box, and in this case the outer cutoff radius should be set consistently with the nonperiodic RMSE computation. Hence, for maximum consistency and generality, the RMSE code by default uses a 5 Å outer cutoff radius for all materials. We used this default (universal) value, rather than removing the outer cutoff radius for the nanoporous solids.

Incidentally, most of the nanoporous solids we studied had atom-centered cavity diameters ≤10 Å, which means their entire cavity was included in the RMSE computation. However, a few (IRMOF-1, Mg-MOF-74, *etc.*) had atom-centered cavity diameters between 10 and ∼15 Å, which means that a small part (<25%) of their cavity volume was excluded from the RMSE computation because it was far away from atoms.

Conceptually, smaller RMSE and RRMSE values indicate a better agreement between the charge method tested and the quantum mechanical benchmark, which is desirable. Conversely, a RRMSE > 1 indicates the method performs worse than the null model; in other words, setting all the atomic charges for a particular system to zero would be better than the charge method tested. An RRMSE of ≤0.3 is desirable with the *R*-squared for the model in this case being ≥0.91. *R*-squared and RRMSE are related as follows:

71 *R*-squared = 1 − RRMSE^2^

## 4. Test sets

To test the reliability of the charge assigning methods across different material types, we analyzed a diverse set of chemical systems. These systems included organic molecules, inorganic molecules, heterodiatomic molecules, transition metal complexes, and nanoporous solids. Each category represents a distinct class of materials with different structural characteristics. This diverse set allowed us to evaluate how the methods performed in different circumstances, such as surface *versus* buried atoms, and to determine their effectiveness for both non-periodic (*e.g.*, molecular systems) and periodic (*e.g.*, crystalline) materials. Additionally, we examined the applicability of the methods across different bonding types by including ionic, covalent, and polar-covalent materials. We purposefully chose systems containing many different chemical elements from across the periodic table.

**Fig. 3:**
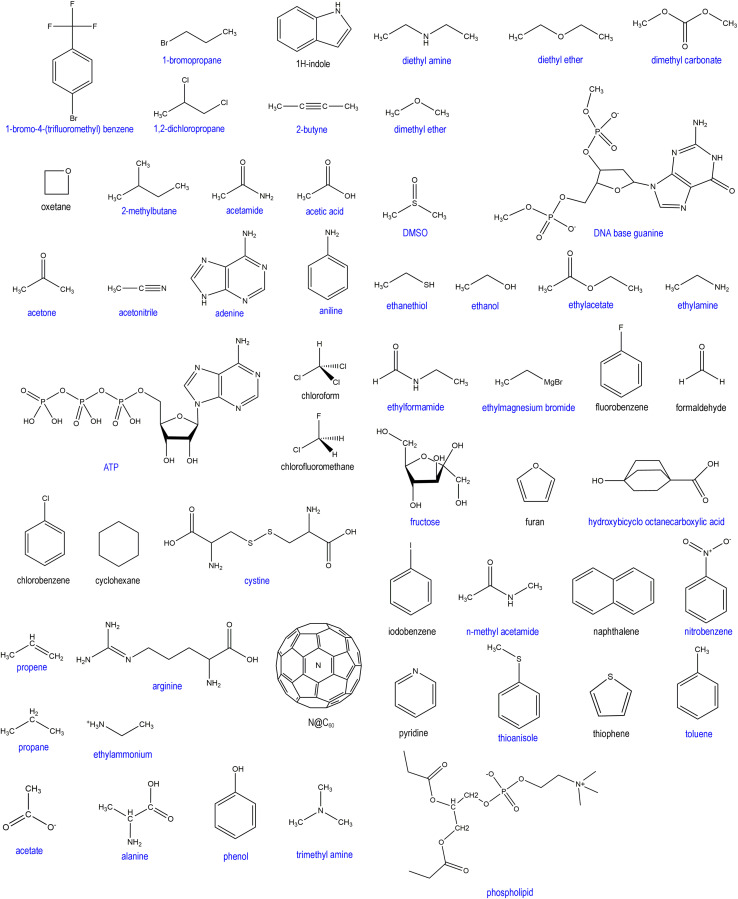
The names and 2D chemical structures of molecules in the organics test set. Molecules with (*cf.* without) any rotatable dihedrals have a blue (*cf.* black) label.

**Fig. 4:**
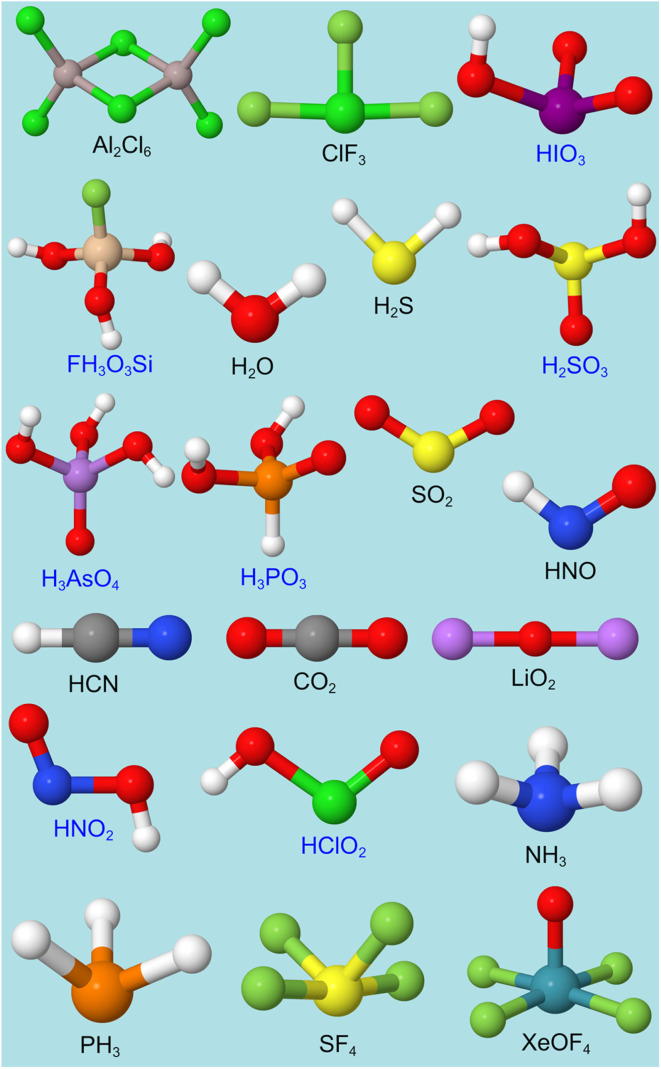
The optimized structures included in the inorganic molecules test set. The atoms are colored by chemical element. Molecules with (*cf.* without) any rotatable dihedrals have a blue (*cf.* black) label.

**Fig. 5:**
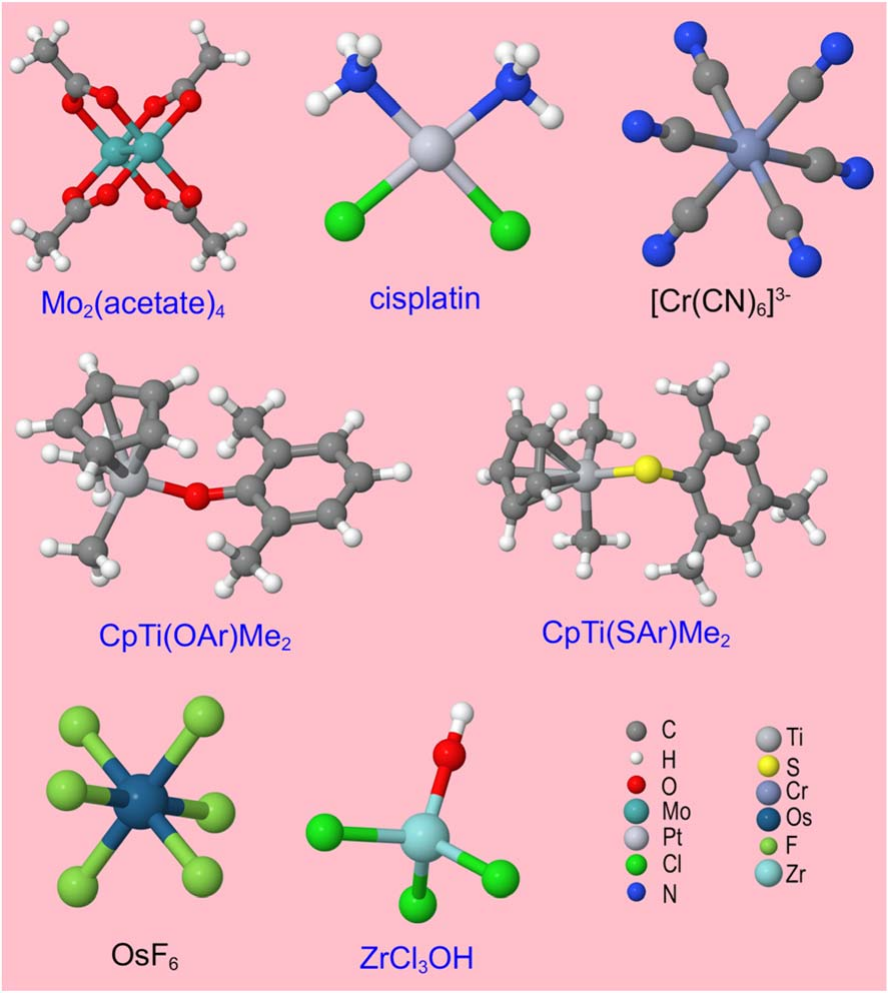
The optimized structures included in the transition metal complexes test set. The atoms are colored by chemical element. Molecules with (*cf.* without) any rotatable dihedrals have a blue (*cf.* black) label.

**Fig. 6:**
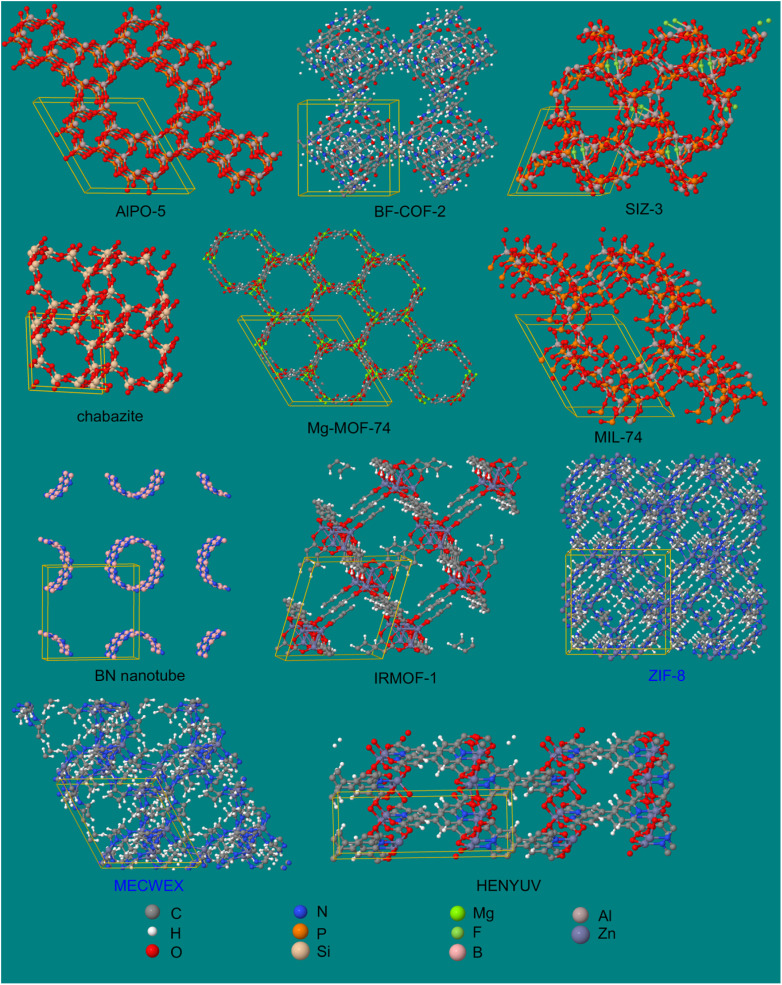
The optimized structures in the nanoporous solids test set. The atoms are colored by chemical element. MECWEX and HENYUV are the Cambridge Structural Database (CSD) refcodes for those two materials. The other nine porous solids are identified by their common names. Materials with (*cf.* without) any rotatable dihedrals have a blue (*cf.* black) label.

Table 6 lists details for the heterodiatomic molecules test set. Fig. 3 shows the 2D chemical structure of each molecule in the organics test set. ESI Table S1† lists the chemical formula and compound class for each of these organic molecules. Fig. 4 shows the optimized 3D chemical structure of each molecule in the inorganic molecules test set. ESI Table S2† lists their chemical formulas. Fig. 5 shows the optimized 3D chemical structure of each molecule in the transition metal complexes test set. ESI Table S3† lists their chemical formulas. Fig. 6 shows the optimized 3D chemical structures of the nanoporous crystals. ESI Table S4† lists the chemical formula, framework type, and unit cell parameters for each nanoporous crystal. In Fig. 3–6, chemical compounds with any rotatable dihedrals have a blue label, while those without any rotatable dihedrals have a black label. Table 7 lists the test systems having non-zero net charge and/or non-singlet spin state.

**Table 6:** Molecules included in the heterodiatomics test set with their optimized bond lengths and the net atomic charges on the first atom of each molecule for the different charge assigning methods

Chemical formula	Optimized bond length (Å)	Net atomic charge of first atom								
		ADCH	CHELPG	CM5	DDEC6	QDR-DDEC6	Hirshfeld	MBIS	MK	RESP
AgBr	2.43	0.45	0.51	0.37	0.47	0.46	0.33	0.47	0.47	0.47
BaS	2.53	0.87	0.98	0.91	1.14	0.91	0.68	1.16	0.96	0.96
CO	1.13	−0.02	−0.02	0.13	0.13	−0.004	0.08	0.13	0.00	0.00
CsLi	3.75	0.18	0.02	0.18	0.30	0.25	0.18	0.40	0.03	0.03
CuI	2.40	0.38	0.39	0.33	0.41	0.38	0.30	0.55	0.38	0.38
HCl	1.28	0.18	0.19	0.17	0.26	0.20	0.12	0.31	0.21	0.21
KF	2.22	0.83	0.82	0.83	0.90	0.85	0.64	0.85	0.83	0.83
NaCl	2.37	0.77	0.77	0.69	0.88	0.81	0.57	0.94	0.78	0.78
NO	1.15	−0.02	−0.02	−0.03	0.03	−0.02	0.03	0.03	−0.02	−0.02
SrO	1.94	0.95	1.03	1.06	1.18	0.98	0.70	1.15	1.02	1.02
ZnO	1.72	0.70	0.79	0.56	0.72	0.70	0.45	0.84	0.76	0.76

**Table 7:** List of test systems having non-zero net charge and/or non-singlet spin states. All test systems not listed in this table had a net charge of zero and spin multiplicity of 1 (*i.e.*, spin singlet)

System	Chemical formula	Net charge	Spin multiplicity
Acetate ion	[C_2_H_3_O_2_]^−^	−1	1
DNA base guanine	[C_12_H_17_N_5_O_10_P_2_]^2−^	−2	1
Ethylammonium ion	[C_2_H_8_N]^+^	+1	1
N@C_60_	N@C_60_	0	4
NO	NO	0	2
Chromium hexacyanide anion	[Cr(CN)_6_]^3−^	−3	4
Osmium hexafluoride	OsF_6_	0	3

For the DNA base guanine, we studied the charged deprotonated state illustrated in Fig. 3. For phospholipid, we studied the zwitterionic form illustrated in Fig. 3. For the alanine, arginine, and cystine amino acids and adenosine triphosphate (ATP), we studied the protonation states illustrated in Fig. 3, which are preferred for the gas-phase (*i.e.*, isolated) molecules. These differ from the preferred protonation states of these molecules in aqueous solutions. We used the preferred gas-phase (*i.e.*, isolated molecule) protonation states, because our AIMD simulations were performed for the isolated, unsolvated molecules.

## 5. Results

### 5.1 Electron-density partitioning that approximately reproduces the molecular total dipole and traceless quadrupole moments

The QDR-DDEC charges are not a chemical correction to the DDEC net atomic charges, but rather they are an atom-centered polyatomic multipole re-expansion that moves (‘resorbs’) part of the atomic dipole and atomic quadrupole contributions into the adjusted point-charge values. This allows the QDR-DDEC point-charge model to more accurately reproduce molecular dipole and quadrupole moments and the electrostatic potential surrounding a material.

Our QDR method purposefully does not incorporate hard constraints that would force (or attempt to force) the assigned atomic charges to exactly reproduce the molecular dipole and/or traceless quadrupole moment. It would not always be possible to satisfy such constraints using just an atom-centered point-charge model. For example, the molecular quadrupole moment about the bond's midpoint cannot be precisely reproduced for the CO or N_2_ molecules using just an atom-centered point-charge model. Moreover, if one places a CO or N_2_ molecule in an external electric field, then the molecular dipole moment component(s) that are not parallel to the bond cannot be reproduced by just an atom-centered point-charge model. As a consequence of these issues, imposing a hard constraint on the point-charge-model's total dipole and/or traceless quadrupole moment values would be counter-productive, because this would cause the coefficients matrix to be ill-conditioned in some systems. An ill-conditioned coefficients matrix would cause high conformational sensitivity of the assigned atom-centered point-charge values in some materials. Imposing hard constraints on the system's total dipole and traceless quadrupole moments would also violate size consistency. If using a hard constraint, a system comprised of three molecules far apart would impose constraints on the system's overall total dipole and/or traceless quadrupole moment. On the other hand, considering these molecules individually would impose constraints on the total dipole and/or traceless quadrupole moment of each molecule. This could yield different assigned point-charge values for the individual molecules compared to a system in which they are far apart, which means that such hard constraints violate size consistency. For all of these reasons, our QDR method does not incorporate such hard constraints.

Swart *et al.*^93^ introduced a multipole-resorption scheme that uses hard constraints enforced using the method of Lagrange multipliers to assign a point-charge model. Their approach can be applied to resorb multipoles up to any specific order (including dipoles, quadrupoles, and/or octupoles) into the point-charge model. Their approach used an exponential decay function (instead of overlap populations used in QDR) to localize the distributed atomic charges. Their approach incorporated off-site charges for some small molecules such as homodiatomics and carbon monoxide.

Our QDR method has some analogies to how the ADCH method corrects the Hirshfeld charges to reproduce the total molecular dipole moment. In another approach, the dipole preserving and polarization consistent (DPPC) method corrects the Mulliken charges to reproduce the total molecular dipole moment.^92^ However, there are substantial differences in the technical details between our QDR approach and the ADCH and DPPC approaches. Our QDR method resorbs both atomic dipoles and atomic quadrupoles into the adjusted point-charge values to produce a new atom-centered polyatomic multipole expansion (including residual atomic dipoles and residual atomic quadrupoles) that is net-equivalent to the original expansion up to quadrupole order. The ADCH and DPPC methods resorb the atomic dipoles but not the atomic quadrupoles, and they are only net-equivalent to the original expansion up to dipole order. ADCH and DPPC use other distance-dependent functions (instead of overlap populations used in QDR) to localize the distributed atomic charges.^86,92^

The ADCH and DPPC methods attempt to constrain the point-charge model's total dipole moment to exactly reproduce the nonperiodic system's total dipole moment; this hard constraint is incorporated in their loss functions using the method of Lagrange multipliers.^86,92^ However, to fix cases in which the ADCH or DPPC coefficients matrix is singular, those methods use a small shift (*e.g.*, +10^−5^) in the eigenvalues as proposed by Thole and van Duijnen.^86,87,92^ This eigenvalue shift causes the ADCH or DPPC method to approximately rather than exactly reproduce the molecular dipole moment in such cases. For the nonperiodic systems studied here, the CsLi molecule was an example of such a case. For CsLi, the analytic dipole moment from the DFT-computed electron density was 1.695 a.u., while the dipole moment from the ADCH point charges was 1.239 a.u. This CsLi molecule accounted for the majority of the ADCH method's small but non-zero molecular dipole moment error listed in Table 8.

Summary statistics quantifying the accuracy of the ADCH, CM5, DDEC6, QDR-DDEC6, Hirshfeld, and MBIS methods for reproducing the QM-computed dipole and quadrupole moments. The dataset includes the optimized ground-state geometry of all molecules and ions in the combined nonperiodics dataset. The RMSE values outside parentheses (*cf.* inside parentheses) are for the atom-centered point-charge model (*cf.* for the model including both atomic charges and atomic dipole moments). For the DDEC6, QDR-DDEC6, Hirshfeld, and MBIS methods, the RMSE values are zero (not shown) for the molecular traceless quadrupole tensor when the model includes atomic charges, atomic dipole moments, and atomic quadrupole moments

**Table:** 

	Mean error molecular dipole magnitude (a.u.)	RMSE molecular dipole magnitude (a.u.)	RMSE molecular dipole vector (a.u.)	RMSE molecular traceless quadrupole tensor (a.u.)	rms Atomic dipole magnitude (a.u.)	rms Atomic quadrupole magnitude (a.u.)
Null model	−1.056	1.656	1.656	12.17	n.a.	n.a.
ADCH	−0.005	0.045	0.045	1.98	n.a.	n.a.
CM5	−0.049	0.155	0.169	1.99	n.a.	n.a.
DDEC6	+0.096 (0.00)	0.258 (0.00)	0.279 (0.00)	1.90 (0.78)	0.112	0.212
QDR-DDEC6	+0.032 (0.00)	0.069 (0.00)	0.082 (0.00)	1.54 (0.30)	0.099	0.071
Hirshfeld	−0.252 (0.00)	0.355 (0.00)	0.388 (0.00)	3.44 (1.44)	0.154	0.378
MBIS	+0.176 (0.00)	0.320 (0.00)	0.335 (0.00)	2.35 (0.97)	0.132	0.217

**Table:** 

	RME molecular dipole magnitude	RRMSE molecular dipole magnitude	RRMSE molecular dipole vector	RRMSE molecular traceless quadrupole tensor
ADCH	−0.5%	2.7%	2.7%	16.3%
CM5	−4.6%	9.4%	10.2%	16.4%
DDEC6	+9.1% (0.0%)	15.6% (0.0%)	16.8% (0.0%)	15.6% (6.4%)
QDR-DDEC6	+3.0% (0.0%)	4.2% (0.0%)	4.9% (0.0%)	12.7% (2.5%)
Hirshfeld	−23.9% (0.0%)	21.5% (0.0%)	23.5% (0.0%)	28.2% (11.9%)
MBIS	+16.7% (0.0%)	19.3% (0.0%)	20.2% (0.0%)	19.3% (8.0%)

**Fig. 7:**
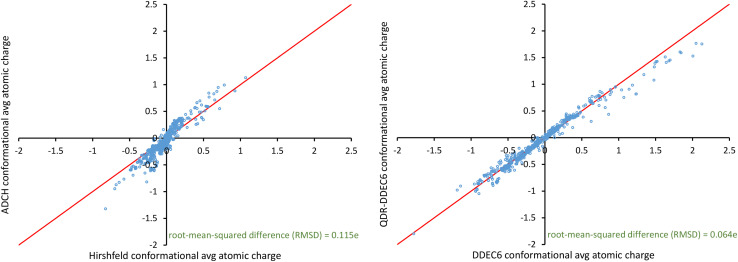
Left panel: Parity plot comparing ADCH to Hirshfeld conformation-averaged atomic charges for the entire nonperiodics dataset. Right panel: Parity plot comparing QDR-DDEC6 to DDEC6 conformation-averaged atomic charges for the combined nonperiodics dataset (see Section 4 for a list of materials in this dataset).

Fig. 7 uses parity plots to compare the ADCH to Hirshfeld atomic charges (left panel) and the QDR-DDEC6 to DDEC6 atomic charges (right panel) for the optimized ground-state geometry of each molecule in the nonperiodics dataset. Both the ADCH and QDR schemes resulted in modest adjustments to the underlying Hirshfeld and DDEC6 charges, respectively.

We computed the QM molecular dipole and traceless quadrupole moments using the molecule's center-of-mass as the reference point. For purposes of computing the molecule's center-of-mass, the mass of each atom was set equal to the standard atomic weights averaged over natural isotope abundances as reported in ref. 124. These molecular dipole and traceless quadrupole moments were computed for the optimized ground-state geometry of each molecule in the combined nonperiodics dataset, which contained 54 organic molecules, 11 heterodiatomic molecules, 20 inorganic molecules, and 7 transition metal complexes (another popular convention in the literature is to use the molecule's center-of-nuclear-charge as the reference point for computing molecular multipole moments. The QDR program provided in the ESI† first computes and prints the molecular dipole and quadrupole moments using the center-of-nuclear-charge as the reference point, and then it recomputes and prints these using the center-of-mass as the reference point).

**Fig. 8:**
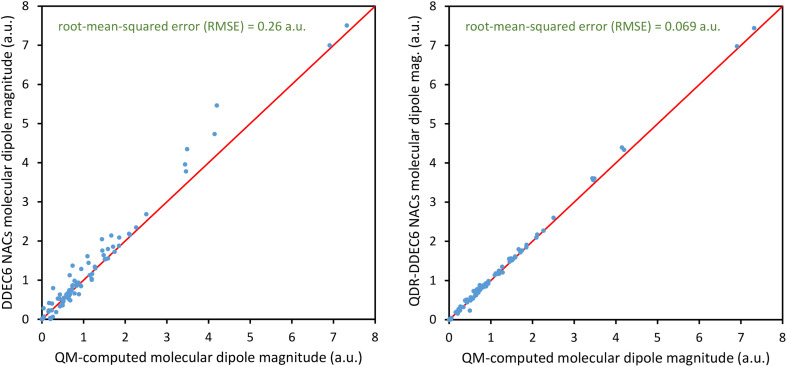
Left panel: Parity plot of DDEC6 *versus* QM-computed molecular dipole magnitude for the optimized geometries of all molecules and ions in the nonperiodics dataset. Right panel: Parity plot of QDR-DDEC6 *versus* QM-computed molecular dipole magnitude for the optimized geometries of all molecules and ions in the combined nonperiodics dataset.

Fig. 8 compares the DDEC6 (left panel) and QDR-DDEC6 (right panel) point-charge model total dipole moment magnitude to the QM-computed value for the optimized ground-state geometry of each molecule in the nonperiodics dataset. The DDEC6 point-charge model already performed well and was substantially further improved by the QDR adjustment.

As shown in Table 8, we computed several quantitative descriptors that quantify how accurately each model reproduces the reference QM-computed molecular dipole and quadrupole moments (all quadrupole moments in Table 8 use the convention shown in eqn (16)). The mean error in the dipole moment magnitude was defined as72
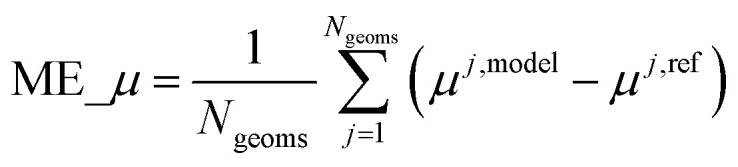
The relative mean error (RME) was defined as the ME divided by the average dipole magnitude:

73 RME_*μ* = ME_*μ*/avg_*μ*

74
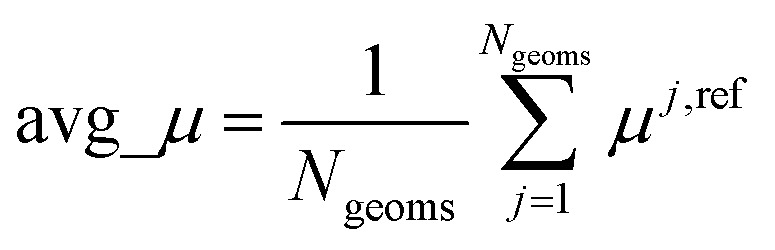
The RME_*μ* can be expressed as a percentage. Examining Table 8, the Hirshfeld (−23.9%) and CM5 (−4.6%) methods were under-polarizing, the MBIS (+16.7%) and DDEC6 (+9.1%) methods were over-polarizing, and the ADCH (−0.5%) and QDR-DDEC6 (+3.0%) methods were nearly on-target for the molecular dipole moment predictions.

The root-mean-squared errors (RMSEs) were defined as75
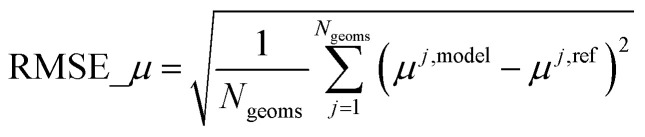
76
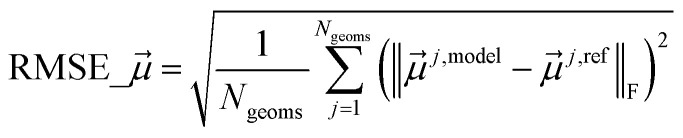
77
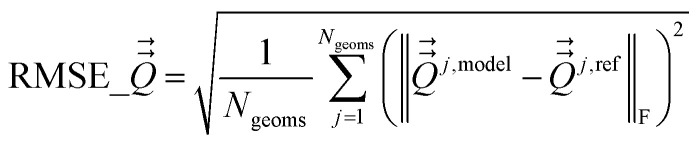


In Table 8, the ‘null model’ is a point-charge-only model with all atomic charges set to zero. For each model, the relative root-mean-squared error (RRMSE) is defined as the RMSE of the model divided by the RMSE of the null model:78
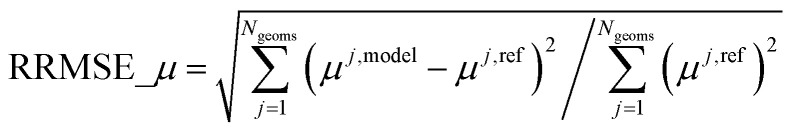
79
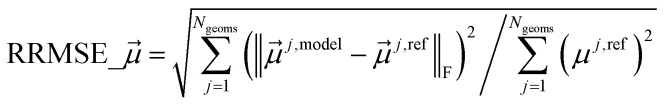
80
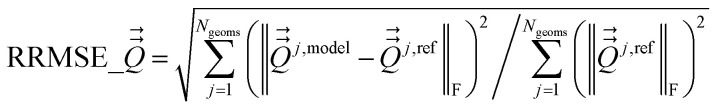
The RRMSE can be expressed as a percentage, and smaller is better.

Among the three stockholder-partitioning methods, DDEC6 showed slightly better accuracy (*i.e.*, smaller RMSE and RRMSE values) than MBIS which performed slightly better than Hirshfeld. This justifies our choice of using DDEC6 as the starting stockholder-partitioning approach to apply our new QDR adjustments. As shown in Table 8, the QDR-DDEC6 method performed better than DDEC6. When atomic dipoles are included in the model, the model predicts the molecular dipole moments exactly: 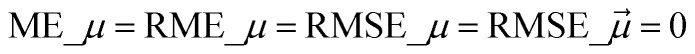. This is a mathematical property of the atom-centered polyatomic multipole expansion.

Our computed results confirmed that the ADCH and CM5 schemes are effective in removing most of the Hirshfeld molecular dipole moment error. The ADCH and CM5 point-charge models predicted the molecular dipole moments more accurately than the DDEC6 point-charge model. However, the DDEC6 point-charge model predicted the molecular quadrupole moments slightly more accurately than the ADCH and CM5 point-charge models (see Table 8).

The root-mean-squared (rms) atomic dipole and traceless quadrupole moment magnitudes were defined as81
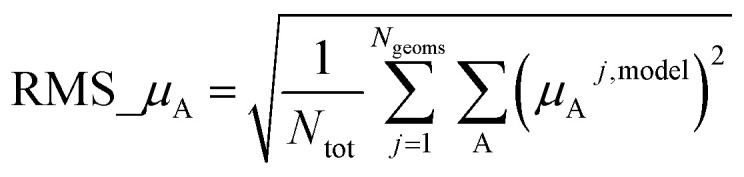
82
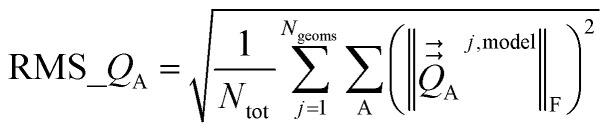
where *N*_tot_ is the total number of atoms in the optimized ground-state geometries of the training set. Smaller RMS_*μ*_A_ and RMS_*Q*_A_ values are desirable, because this means more of the atom-centered polyatomic multipole expansion is captured by the point-charge-only model. Examining Table 8, the RMS_*μ*_A_ and RMS_*Q*_A_ values followed the trend QDR-DDEC6 < DDEC6 < MBIS < Hirshfeld.

### 5.2 Electrostatic potential RMSE and RRMSE values

The following kinds of assessments were performed:

(1) For various charge assignment methods, net atomic charges were calculated for the optimized ground-state geometry. For each material, these ‘optimized conformation’ charges were then used to compute the electrostatic RMSE and RRMSE across the training dataset containing 21 geometries. Please note that the 20 AIMD geometries in this dataset were used to assess the performance of the optimized conformation charges and were not involved in the assignment of these charge values.

(2) For various charge assignment methods, net atomic charges were calculated for each of the 21 geometries of each material in the training dataset. The performance of the ‘individual conformation’ charges is the electrostatic RMSE and RRMSE values computed for each geometry in the training dataset using the charge values that were computed from that same specific chemical geometry. In addition to point-charge-only models, we also tested electrostatic models containing DDEC6, QDR-DDEC6, and MBIS atomic charges plus spherical electron cloud penetration (aka DDEC6_cp, QDR-DDEC6_cp, and MBIS_cp), plus atomic dipoles (aka DDEC6_ad, QDR-DDEC6_ad, and MBIS_ad), and both (aka DDEC6_ad_cp, QDR-DDEC6_ad_cp, and MBIS_ad_cp).^14,32,33^

(3) For each material in the training dataset, the ‘average conformation’ charges are the average of the assigned charge of each atom over the 21 geometries of that same material. That is, the 21 ‘individual conformation’ charges for the training dataset were averaged to compute the ‘average conformation’ charges. The performance of these average conformation charges is the electrostatic RMSE and RRMSE values computed using these ‘average conformation’ charges applied to the training and validation datasets. Their performance for the validation dataset can assess whether over-fitting occurred, because the validation dataset contains entirely new geometries that were not involved in computing the average conformation charges.

(4) For each nonperiodic material, multiframe variants of the CHELPG, MK, and RESP methods were computed in Multiwfn^90,91^ version 3.8 by minimizing the electrostatic RMSE simultaneously across all 21 geometries of the training dataset. These were then tested on the training and validation datasets. Since the grid points used to define the charge values for each of these methods differed from the grid points used to assess their performance, it is possible to obtain RRMSE values >1 if the charge assignment method suffered from an over-fitting problem.

Raincloud plots were prepared for each kind of assessment described above. We plotted these *via* the Python package PtitPrince^125^ using the following parameters. The box denotes the second and third quartiles with the midline indicating the median. The 5th and 95th percentiles are marked by whiskers. Each outlier is plotted as a diamond. Each individual datapoint in the distribution is printed as a jittered point (aka ‘raindrop’) below the kernel density profile (aka ‘cloud’).

**Fig. 9:**
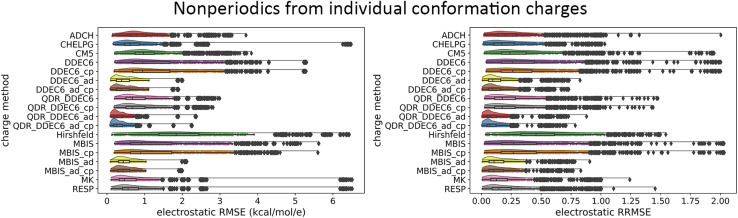
Raincloud plots for the combined nonperiodic materials dataset (*i.e.*, organics, inorganics, heterodiatomics, and transition metal complexes) for different charge assignment methods using individual conformation charges. This contains all materials and geometries in the training dataset.

**Fig. 10:**
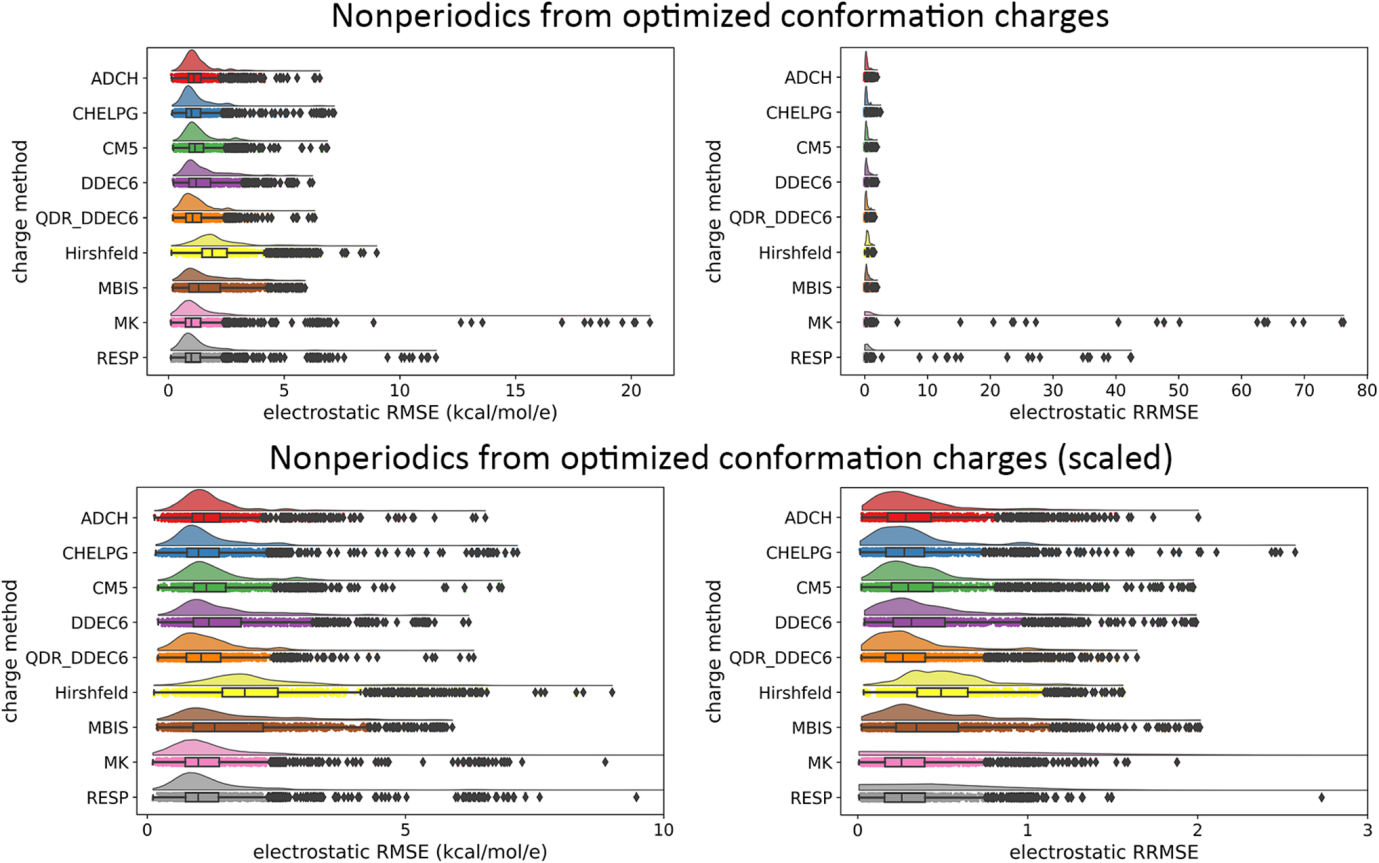
Raincloud plots for the combined nonperiodic materials dataset (*i.e.*, organics, inorganics, heterodiatomics, and transition metal complexes) for different charge assignment methods using the optimized ground-state conformation charges. This contains all materials and geometries in the training dataset. The bottom row uses an enlarged scale to zoom in on the results.

**Fig. 11:**
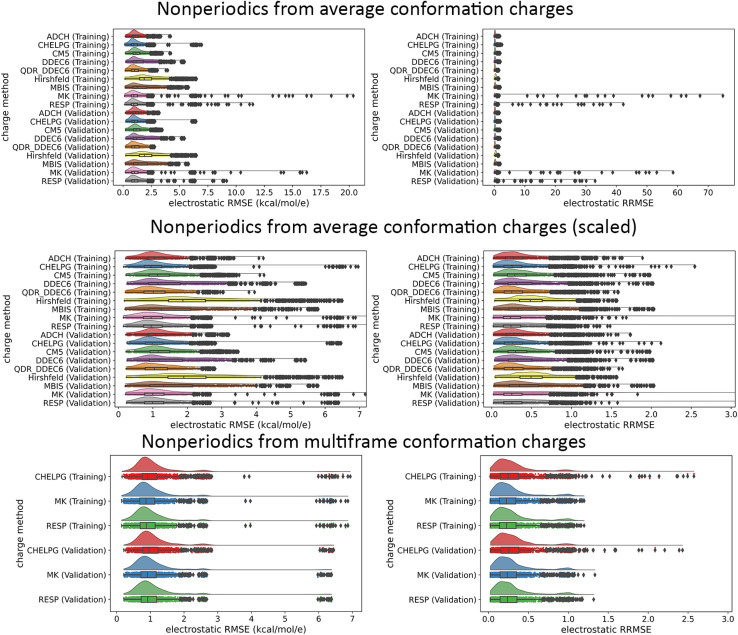
Raincloud plots for the combined nonperiodic materials dataset (*i.e.*, organics, inorganics, heterodiatomics, and transition metal complexes) using average conformation charges (top and middle rows) and using multiframe charges (bottom row) for different charge assignment methods. The middle row uses an enlarged scale to zoom in on the results. These raincloud plots include training and validation data.

Fig. 9 summarizes results of individual conformation charges for all nonperiodic materials in the training dataset. The Hirshfeld charges showed the highest median RMSE and RRMSE values. The electrostatic models including atomic dipoles (*e.g.*, DDEC6_ad, QDR-DDEC6_ad, and MBIS_ad) had better performance than the point-charge-only models. Including cloud penetration had little impact on the results. Among the point-charge-only models, the CHELPG, CM5, QDR-DDEC6, ADCH, DDEC6, and MBIS models performed reasonably well; however, each of these methods produced a few outliers with RRMSE values >1. Although the MK and RESP methods gave low RMSE and RRMSE values for the individual conformations in the training dataset, they did not perform well when transferred across different molecular conformations due to severe over-fitting problems as shown in Fig. 10 and 11.

Fig. 10 contains the raincloud plots showing the performance of the optimized conformation charges across all geometries in the nonperiodic materials training dataset. This quantifies how well the charges optimized on the ground-state structure of each molecule performed across all 21 training set geometries of that molecule. The over-fitting problem of single-frame ESP-fitting methods is apparent.

Fig. 11 contains the raincloud plots showing the performance of the average conformation and multiframe charges across all geometries in the training and validation datasets of the nonperiodic materials. The multiframe ESP-fitting methods were mostly successful in resolving the over-fitting problem of single-frame ESP-fitting methods.

**Fig. 12:**
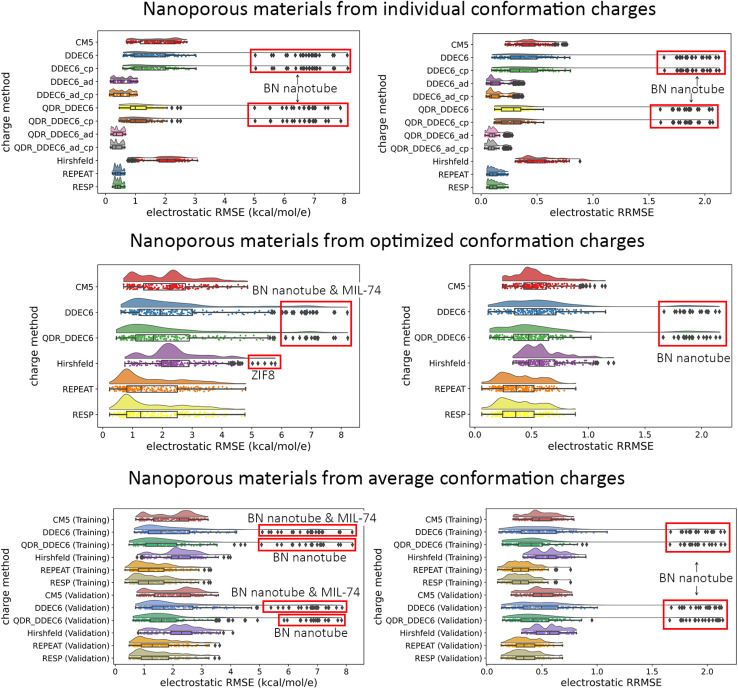
Raincloud plots for the nanoporous materials dataset for different charge assignment methods using individual conformation (top panels), optimized ground-state conformation (middle panels), and average conformation (bottom panels) charges. Data in the top and middle panels is for all materials and geometries in the training dataset, while the bottom panels display results for both the training and validation datasets.

For each point-charge-only model, the distributions of RMSE and RRMSE were broader than desired. Unfortunately, each and every one of the point-charge-only models in Fig. 10–12 had some outliers with RRMSE > 1.0. This strongly shows that better point-charge-only models still need to be developed, or alternatively one should include atomic dipoles in the electrostatic model. This also shows that constructing consistently accurate point-charge-only models is a hard problem.

The ESI† contains separate raincloud plots for: (a) organic molecules, (b) heterodiatomic molecules, (c) inorganic molecules, and (d) transition metal complexes. The following should be considered when interpreting these raincloud plots for different material classes. A feature that consistently appeared across many material classes was more widespread (and thus more likely to be significant) than a feature that appeared in only one or two material classes.

Raincloud plots for the ‘individual conformation’, ‘optimized conformation’, and ‘average conformation’ charges are displayed in Fig. 12 for the nanoporous crystals. Among the point-charge-only models, REPEAT and RESP had the lowest median electrostatic RMSE and RRMSE values for these materials. For this set of periodic solids, the DDEC6 charges produced wider electrostatic RMSE and RRMSE distributions than the CM5 charges. For the DDEC6 and QDR-DDEC6 point charges, the BN nanotube was a notable outlier as discussed in the following section. For individual conformations (top panels), including atomic dipoles (*i.e.*, DDEC6_ad, DDEC6_ad_cp, QDR-DDEC6_ad, and QDR-DDEC6_ad_cp) fixed this problem.

### 5.3 Analysis of the outliers

We define a ‘double outliers’ as any material that has some datapoints with RRMSE > 2.0 and some datapoints with RMSE > 5 kcal mol^−1^ e^−1^ for any charge scheme, including using individual conformation charges, low-energy conformation charges, multiframe charges, or conformationally-averaged charges applied to either the training or validation dataset geometries. To be classified as a ‘double outlier’, the datapoint(s) having RRMSE > 2.0 do not necessarily have to be the same datapoint(s) having RMSE > 5 kcal mol^−1^ e^−1^. For example, if charge method #1 gives RRMSE > 2.0 for training geometries #2, 6, and 8 of a material, while charge method #5 gives RMSE > 5.0 for validation geometries #5 and 20 of this material, then this would be sufficient to classify this material as a ‘double outlier’.

This definition of a ‘double outlier’ identifies materials for which relatively large errors in both RMSE and RRMSE occurred. This definition intentionally excludes materials for which the RMSE is small but the RRMSE is large, and also excludes materials for which the RMSE is large but the RRMSE is small.

Among the set of materials we examined, the only ‘double outliers’ were N@C_60_ and the BN nanotube. There were no ‘double outliers’ amongst the heterodiatomics, inorganics, or transition metal complexes we studied.

The BN nanotube was part of a series of BN structures (*i.e.*, B_4_N_4_ cluster, BN nanotube, BN sheet, and h-BN crystal) that were previously studied as part of the development of the DDEC methods.^30,32–34^ The BN nanotube, BN sheet, and h-BN crystal all have B and N atoms at alternating corners of hexagonal bonded rings. The h-BN crystal is composed of a stack of h-BN sheets with weak dispersion bonds (interlayer B–N bond order = 0.02) between sheets.^38^ Due to their similar chemical bonding structures, one expects the net atomic charges for these three materials should be similar.^38^ Indeed, previous studies reported DDEC6 charges on the B atom equal to +0.83 (BN nanotube), +0.82 (BN sheet), and +0.82 (h-BN crystal).^33,38^ A prior study reported electrostatic potential fitting charges (aka *V*-fit) for the B atom of +0.47 (BN nanotube) and +0.86 (BN sheet) that were optimized to minimize the RMSE and RRMSE.^34^

As shown in Table 9, the REPEAT and RESP methods gave good RRMSE values for this material; however, these two methods suffered an overfitting problem as demonstrated by the relatively large range of assigned N atom charges. For this material, REPEAT and RESP gave essentially identical results with N atom charges ranging from −0.447 to −0.108. Each N atom in the chemical structure has the same covalent bond structure but is not strictly chemically equivalent due to the dispersion interactions between nanotubes caused by the stacking between nanotubes in the crystal lattice. For this reason, one expects small but not large variations in the computed N atom charges. The range of 0.339 for the REPEAT and RESP methods seems excessive indicating an overfitting problem. Each of the other four methods (*i.e.*, CM5, DDEC6, QDR-DDEC6, and Hirshfeld) gave B and N atom charges with ranges <0.13, and this suggests these methods did not suffer an overfitting problem.

**Table 9:** Computed atomic charges for the optimized ground-state BN nanotube structure. The average ± standard deviation are listed as well as the range (*i.e.*, maximum minus minimum). The electrostatic potential RMSE and RRMSE values are also listed

Method	B charge		N charge		RMSE (kcal mol^−1^ e^−1^)	RRMSE
	Avg. ± st. dev.	Range	Avg. ± st. dev.	Range		
CM5	+0.221 ± 0.030	0.090	−0.221 ± 0.018	0.046	2.14	0.64
DDEC6	+0.816 ± 0.035	0.093	−0.816 ± 0.023	0.068	6.81	2.04
QDR-DDEC6	+0.823 ± 0.035	0.093	−0.823 ± 0.041	0.124	6.83	2.04
Hirshfeld	+0.207 ± 0.033	0.096	−0.207 ± 0.021	0.052	2.13	0.64
REPEAT	+0.268 ± 0.051	0.143	−0.268 ± 0.120	0.339	0.39	0.12
RESP	+0.268 ± 0.051	0.143	−0.268 ± 0.120	0.339	0.39	0.12

**Fig. 13:**
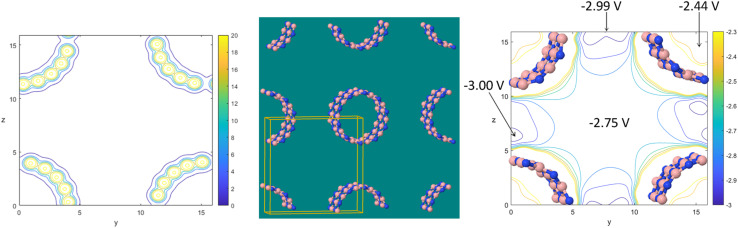
Contour diagrams of the electrostatic potential distribution for the BN nanotube array. The left panel shows the positive voltages inside the atoms. The center panel shows the unit cell (gold box) and positions of the boron (pink) and nitrogen (blue) atoms. The right panel shows the voltages in the empty spaces inside and outside the nanotubes, with the boron (pink) and nitrogen (blue) atom positions overlayed. No atom-centered point-charge-only model can accurately reproduce the potential difference between the empty space inside and outside the nanotube. A model that includes atom-centered point charges plus atomic dipoles can approximately reproduce this electrostatic potential distribution.

A prior study showed the computed bond orders are essentially identical in the BN nanotube and sheet.^38^ Because of symmetry, every atomic dipole is zero in the BN sheet. Atomic dipoles are small but nonzero in the BN nanotube. However, the cumulative effect of these atomic dipoles is additive causing an electrostatic potential difference of ∼0.6 volts between the inside and outside of the BN nanotube, as shown in Fig. 13.

It is manifestly clear that an atom-centered point-charge-only model cannot exhibit chemical transferability and low RRMSE values simultaneously for the BN sheet and BN nanotube. Specifically, if we adopt a B atom charge value of approximately +0.86, this minimizes the RRMSE for the BN sheet but gives a RRMSE of ∼2 for the BN nanotube. On the other hand, if adopt a B atom charge value of approximately +0.27 (see REPEAT method in Table 9), this approximately minimizes the RRMSE for the BN nanotube over the grid points used in this work, but it will give relatively high RRMSE values for the BN sheet. Note: the difference between *V*-fit B atom charges for the BN nanotube of +0.47 in the prior study^34^ and +0.27 in this study is due to selecting different grid points (for computing RMSE) and unit cell lengths in these two studies.

A BN nanotube has a cylindrical geometry with opposite charges on the B and N atoms. Since the net charge is zero and both atoms are located at the same radial value, every atom-centered point-charge-only model gives the same electrostatic potential value in the empty space inside the BN nanotube as in the empty space outside the BN nanotube. As shown in Fig. 13, the QM-computed potential difference is ∼0.6 volts between the empty space inside and outside the nanotube in this array. Since no atom-centered point-charge-only model can produce such a nonzero potential difference, we conclude that one must include atomic dipoles to describe such an effect. Higher-order atomic multipoles (*e.g.*, quadrupoles, octupoles, *etc.*) may be optionally included, but they are not strictly required to describe such an effect.

When atomic dipoles are included, the RMSE improves to 1.02 (DDEC6_ad) and 0.25 (QDR-DDEC6_ad) kcal mol^−1^ e^–1^, and the RRMSE improves to 0.31 (DDEC6_ad) and 0.07 (QDR-DDEC6_ad) for the BN nanotube's optimized geometry. Including both atomic dipoles and cloud penetration gives RMSE of 1.00 (DDEC6_ad_cp) and 0.22 (QDR-DDEC6_ad_cp) kcal mol^−1^ e^–1^, and the RRMSE equals 0.30 (DDEc6_ad_cp) and 0.06 (QDR-DDEC6_ad_cp). Including cloud penetration but not atomic dipoles gives RMSE of 6.83 (DDEC6_cp) and 6.84 (QDR-DDEC6_cp) kcal mol^−1^ e^–1^, and the RRMSE equals 2.04 (DDEC6_cp) and 2.05 (QDR-DDEC6_cp) for the BN nanotube's optimized geometry. Thus, including atomic dipoles rather than charge penetration results in the most improvement.

If using a point-charge-only model, moderate RRMSE values can be obtained by setting the B atomic charge to a comprise value that is between the *V*-fit values for the BN sheet and nanotube. For example, if one sets *q*_B_ = ∼0.5, then this can yield transferable results for the BN sheet and nanotube with RRMSE < 1.0. There is a higher penalty for over-estimating the point-charge magnitude than for under-estimating the point-charge magnitude. Specifically, if one sets *q*_B_ = 0.0, then by definition this yields RRMSE = 1.0. Under-estimating the point-charge magnitudes (as long as one gets the charge-transfer direction correct) typically yields RRMSE < 1.0, because it does not over-shoot. In contrast, over-estimating the point-charge magnitudes can sometimes produce RRMSE > 1.0 due to over-shooting. Thus, it is ‘safer’ to under-estimate rather than to over-estimate the point-charge magnitudes when creating an electrostatic model. This explains why the CM5 model, which has a smaller rms charge transfer magnitude (see Table 11) than the DDEC6 model and ESP-fitting approaches, also produced narrower electrostatic RMSE and RRMSE distributions than the DDEC6 point-charge model. This strongly indicates that it would be useful to develop a seventh-generation DDEC approach that has a smaller rms charge transfer magnitude than the sixth-generation (aka DDEC6) method.

Table 10 summarizes the performance of various point-charge methods for the N@C_60_ endohedral complex. In this work as well as in a prior study, the MK and RESP methods gave extremely large and chemically implausible values for the N atom charge in the N@C_60_ endohedral complex.^36^ This gave rise to the humongous RMSE and RRMSE outliers for these two methods in Fig. 11. For all of the other charge assignment methods, the maximum validation dataset RMSE for the N@C_60_ molecule was less than 1 kcal mol^−1^ e^−1^.

**Table 10:** Performance of different charge assignment methods for the N@C_60_ endohedral complex. The RMSE and RRMSE values for the validation dataset were computed using the conformation averaged charges or the multiframe charges

Method	N charge training set geoms.			RMSE (kcal mol^−1^ e^−1^) validation set		RRMSE validation set	
	Min.	Avg.	Max.	Avg. ± st. dev.	Max	Avg. ± st. dev.	Max
ADCH	0.101	0.110	0.118	0.33 ± 0.06	0.51	1.06 ± 0.07	1.22
CHELPG	0.310	0.321	0.332	0.44 ± 0.11	0.62	1.46 ± 0.36	2.12
MF-CHELPG	0.325	0.325	0.325	0.47 ± 0.14	0.71	1.53 ± 0.48	2.43
CM5	0.084	0.091	0.099	0.32 ± 0.06	0.54	1.04 ± 0.07	1.20
DDEC6	0.131	0.139	0.146	0.35 ± 0.07	0.57	1.12 ± 0.13	1.40
QDR-DDEC6	0.136	0.143	0.150	0.34 ± 0.07	0.56	1.12 ± 0.11	1.39
Hirshfeld	0.107	0.112	0.118	0.33 ± 0.06	0.55	1.07 ± 0.09	1.28
MBIS	0.101	0.106	0.114	0.33 ± 0.07	0.55	1.07 ± 0.09	1.26
MK	8.70	9.47	10.75	9.0 ± 4.7	16.2	30.2 ± 16.1	58.5
MF-MK	0.015	0.015	0.015	0.31 ± 0.06	0.50	1.00 ± 0.01	1.01
RESP	4.74	5.36	6.05	5.1 ± 2.6	9.2	17.1 ± 9.1	33.0
MF-RESP	0.015	0.015	0.015	0.31 ± 0.06	0.50	1.00 ± 0.01	1.01

### 5.4 Comparing various figures of merit for different charge assignment methods

The root-mean-squared (rms) charge transfer magnitude of each charge assignment method was quantified by computing83
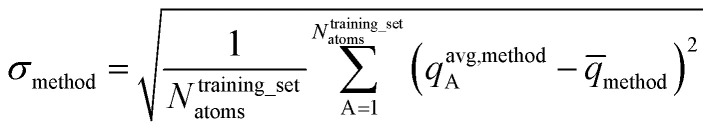
where *q*^avg,method^_A_ is the conformation-average charge84
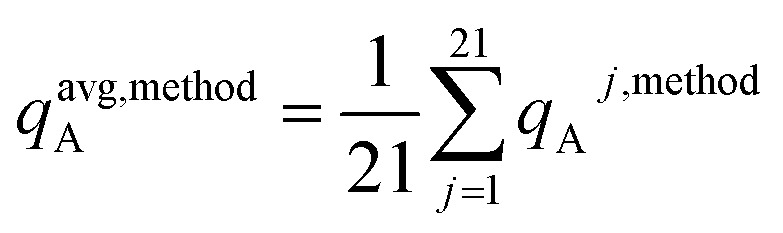
In eqn (84), *j* is the conformation number in the training dataset, which contained 21 different conformations for each material. In eqn (83), *N*^training_set^_atoms_ is the number of atoms in the training set. The combined nonperiodics training set contained 1094 atoms. In eqn (83), *q̄*_method_ is the overall average atomic charge in the training set for that particular charge assignment method:85
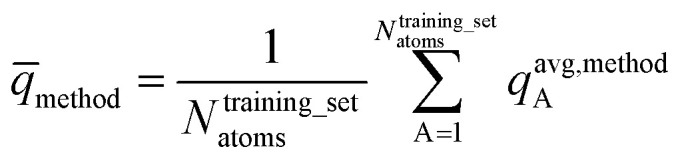
Because the training set contains some charged molecules (see Table 7), *q̄*_method_ is close to but not exactly equal to zero. For the multiframe charges, *σ*_method_ and *q̄*_method_ were computed using analogs of eqn (83) and (85) that replaced *q*^avg,method^_A_ with *q*^multiframe,method^_A_.

As shown in Table 11, the ADCH and CM5 methods gave substantially smaller rms charge transfer magnitudes than the multiframe ESP (*i.e.*, MF-CHELPG, MF-MK, and MF-RESP) methods. The DDEC6 and QDR-DDEC6 methods gave slightly higher rms charge transfer magnitudes than the multiframe ESP methods. The MBIS and MK methods substantially over-estimated the rms charge transfer magnitude, while the Hirshfeld method severely under-estimated the rms charge transfer magnitude.

**Table 11:** Various figures of merit for several methods that assign atomic charges. The rms charge transfer magnitude, sensitivity of NACs, normalized sensitivity of NACs, and summed correlations were computed across the training datasets containing all of the nonperiodic materials. The listed validation dataset median RRMSE is using the conformation-averaged or multiframe (MF) charges. The listed training dataset median RRMSE is for all training dataset geometries using the optimized conformation charges

Method	rms Charge transfer magnitude (*e*)	Median RRMSE validation dataset	Median RRMSE training dataset	Sensitivity of NACs (*e*)	Normalized sensitivity of NACs	Summed correlations (*S*_*α*_)	Number (*Ω*_*αβ*_ > 0.9)	Electron density partition?	Works for dense crystalline solids?
ADCH	0.246	0.278	0.283	0.033	0.133	10.80	8	No	a b
CHELPG	0.371	0.263	0.275	0.048	0.128	11.15	7	No	No
MF-CHELPG	0.356	0.257	—	—	—	11.12	8	No	No
CM5	0.254	0.297	0.297	0.013	0.051	10.86	8	No	Yes^b^^c^
DDEC6	0.388	0.317	0.316	0.019	0.050	11.13	10	Yes	Yes^b^
QDR-DDEC6	0.379	0.265	0.266	0.023	0.060	11.27	10	d	Yes^b^
Hirshfeld	0.163	0.492	0.490	0.008	0.052	10.73	9	Yes	Partly^b^^e^
MBIS	0.448	0.348	0.345	0.024	0.053	11.12	10	Yes	b f
MK	0.470	0.251	0.257	0.063	0.133	9.45	2	No	No
MF-MK	0.337	0.235	—	—	—	11.21	10	No	No
RESP	0.399	0.252	0.260	0.063	0.157	10.61	2	No	No
MF-RESP	0.335	0.238	—	—	—	11.20	10	No	No

a To the best of our knowledge, the ADCH method has not yet been applied to dense crystalline solids.

b Electrides are materials in which an electron not associated with a particular atom acts as an anion. These charge partitioning methods are not expected to give reliable results for electrides.

c The CM5 method works for most dense solids at ambient pressures; however, it overestimates charge transfer magnitudes in some dense solids under high pressures (see Table 12).

d The QDR-DDEC6 charges do not explicitly equal an integrated stockholder-partitioned electron density distribution; however, they are derived from the DDEC6 stockholder-partitioned atom-in-material charges and atomic dipoles.

e The Hirshfeld method typically underestimates the charge transfer magnitudes.

f MBIS was applied to a small number of dense solids in ref. 14; however, the MBIS method has not yet been widely tested on a large number of dense solids.

**Fig. 14:**
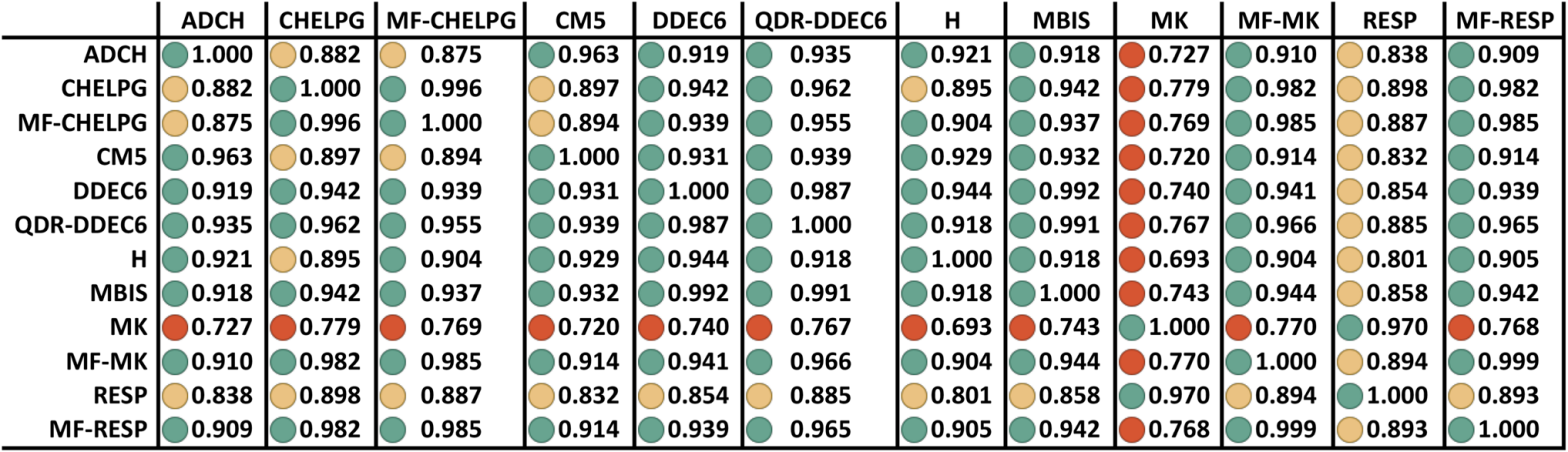
Correlation matrix between 12 methods for assigning net atomic charges in molecules. For methods assigning charges to individual conformations, correlations between the conformation-averaged charge values were used. MF denotes the multiframe variants (*i.e.*, multiframe-fitted charges) of the electrostatic potential fitting methods. Stoplight colors indicate the correlation values: green ≥ 0.9, 0.8 ≤ yellow < 0.9, red < 0.8.

As explained in Manz's article introducing the Seven Confluence Principles, the summed correlations (*S*_α_) and the number of strong correlations (*i.e.*, number of *Ω*_*αβ*_ > 0.9) can be used to identify the most confluent descriptor within a positively correlated descriptor group.^36^ In the present work, we defined the covariance matrix *Λ*_*α*,*β*_ and the correlation matrix *Ω*_*α*,*β*_ between two charge assignment methods *α* and *β* as:86
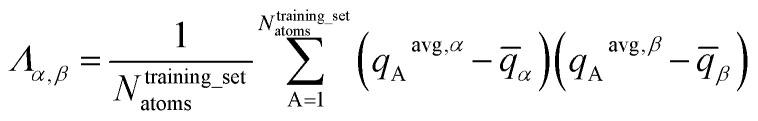
87
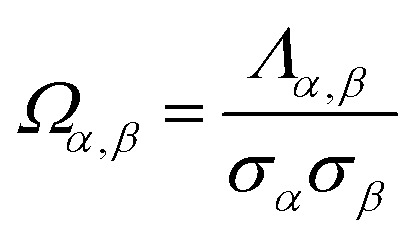
88
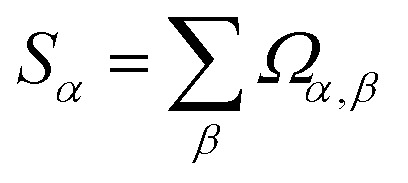
where *σ*_*α*_ and *q̄*_*α*_ are computed *via*eqn (83) and (85), respectively. Fig. 14 illustrates the correlation matrix between the 12 charge assignment methods listed in Table 11. Among these 12 methods, the QDR-DDEC6, MF-MK, and MF-RESP charges were the most confluent, which means these methods are the best representatives of the entire descriptor group.

The following key points should be kept in mind. The multiframe ESP (*i.e.*, MF-CHELPG, MF-MK, and MF-RESP) charges cannot be computed for a single geometry but instead require simultaneously optimizing the atomic charge values across a training set containing many geometries (conformations) of the same material. In contrast, the QDR-DDEC6 charges can be computed for individual geometries of a material. This makes the QDR-DDEC6 charges more convenient to compute than multiframe ESP charges.

In the present work, we defined the conformational sensitivity of each charge assignment method as89
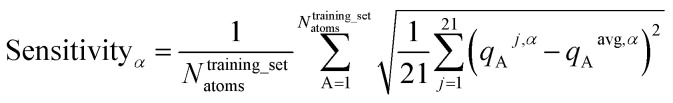
The Hirshfeld and CM5 methods that assign relatively small magnitudes to the net atomic charges have small conformational sensitivities. Thus, it is also useful to define a normalized conformational sensitivity which divides the sensitivity by the rms charge transfer magnitude:90
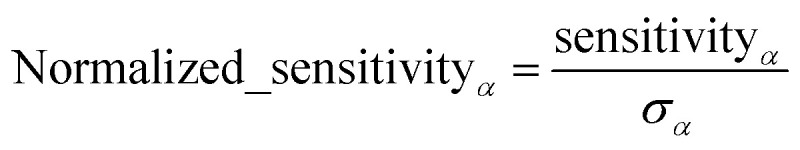
The normalized conformational sensitivity is unitless. The DDEC6, CM5, and Hirshfeld methods had the smallest normalized sensitivities. The ESP-fitting methods (*e.g.*, MK, RESP, CHELPG) had the largest sensitivities and large normalized sensitivities.

**Fig. 15:**
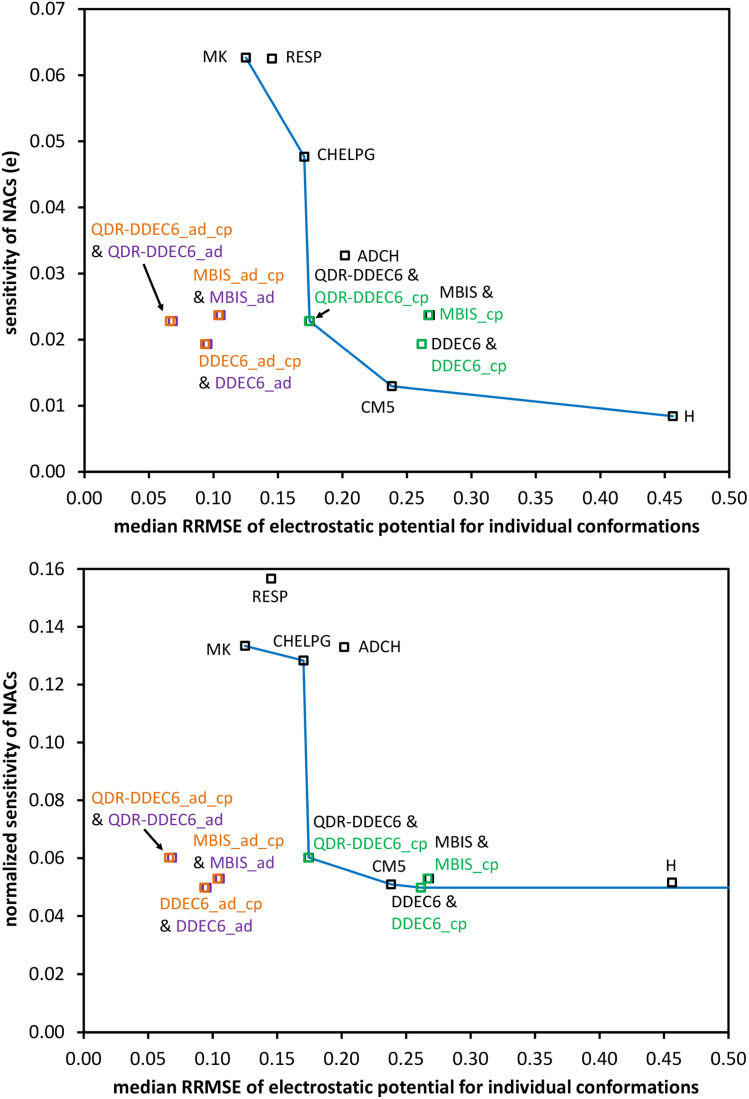
Pareto plot illustrating trade-offs between the accuracy for reproducing the electrostatic potential (median RRMSE plotted on *x*-axis) and the conformational sensitivity (rms charge deviation plotted on *y*-axis) for several methods that assign atomic charges in nonperiodic materials.

Among methods that assign atomic charges to individual geometries of a material, there is a tradeoff between the conformational sensitivity of the atomic charge values and the accuracy of the corresponding atom-centered point-charge model for approximately reproducing the electrostatic potential around the material. Analogous to ref. 14, this tradeoff is visualized by the Pareto plots shown in Fig. 15. Examining Fig. 15, the QDR-DDEC6 and CM5 methods provided the best tradeoff between the conformational sensitivity of the atomic charge values and the accuracy of the corresponding atom-centered point-charge model for approximately reproducing the electrostatic potential around the material.

For comparison, Fig. 15 also includes results for the DDEC6, MBIS, and QDR-DDEC6 methods with atomic dipoles (labeled_ad), spherical cloud penetration (labeled_cp), and both (labeled_ad_cp). Cloud penetration was negligible, because the grid points used to compute the RRMSE were almost entirely outside the material's electron density distribution. Cloud penetration would become important at shorter distances. Including atomic dipoles reduced the RRMSE substantially. MBIS_ad, DDEC6_ad, and QDR-DDEC_ad all gave lower median RRMSE than the ESP-fitted point-charge-only models (MK, RESP, CHELPG). Among all of these models, the lowest median RRMSE were 0.066 (QDR-DDEC6_ad_cp) and 0.068 (QDR-DDEC6_ad).

Table 11 lists the median RRMSE for the nonperiodics validation set using the conformation averaged or multiframe charges. The multiframe ESP methods performed well on this metric. The QDR-DDEC6, ADCH, and CM5 methods also performed well on this metric. Table 11 also lists the median RRMSE for the nonperiodics training set using the optimized conformation charges. Although the single-frame ESP methods performed well on both median RRMSE metrics, they are not recommended owing to the over-fitting problem that causes high conformational sensitivity (see Fig. 15) and some extreme outliers (see Fig. 10). The QDR-DDEC6, ADCH, and CM5 methods performed well on this metric and avoid extreme outliers in RRMSE. As shown in Fig. 15, the QDR-DDEC6 and CM5 methods give a good combination of RRMSE accuracy and low conformational sensitivity.

In addition to approximately reproducing the electrostatic potential surrounding a material, net atomic charges are also important descriptors of the chemical states of atoms in materials. A key consideration is whether the charge assignment method works for dense solids. Because dense crystalline solids lack a van der Waals surface, ESP fitting methods that optimize the net atomic charge values to approximately reproduce the QM-computed electrostatic potential outside a material's van der Waals surface do not work for dense crystalline solids.

Another key consideration is whether the method explicitly partitions the material's electron cloud into atom-in-material electron density distributions that sum up to the material's total electron density distribution (see eqn (1)). This is extremely important for two reasons. First, it naturally places bounds on the range of values the net atomic charges can have. Because electron density partitioning approaches assign a non-negative number of electrons to each atom in the material, they assign a net atomic charge ≤+7.0 to the nitrogen atom in N@C_60_. In stark contrast, ESP fitting methods sometimes assign chemically impossible values to the net atomic charges. For example, as shown in Table 10, the MK method assigned the chemically impossible values of 8.70–10.75 to the net atomic charge of the nitrogen atom in N@C_60_. Second, some electron density partitioning methods provide access to many chemical descriptors beyond net atomic charges, such as atom-in-material multipoles,^42,43,51,52^ bond orders,^37,38^ overlap populations,^59,126^ atomic spin moments,^37,65^ short-range repulsion parameters,^17–19^ polarizabilities and dispersion coefficients,^20,21,56,127,128^*etc.*^31^

**Fig. 16:**
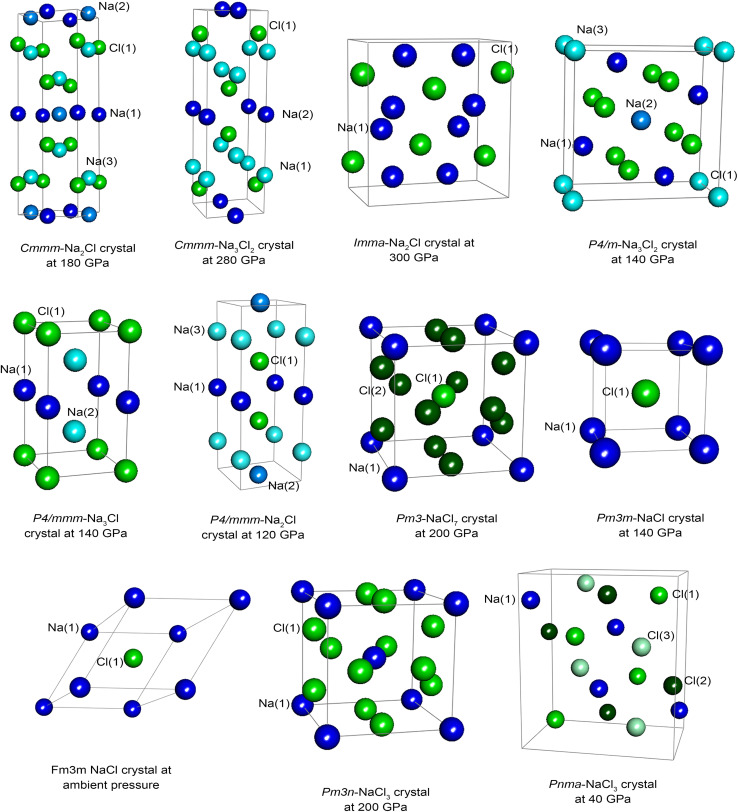
Sodium chloride crystal structures. The lines mark the unit cell boundaries (reproduced with permission from ref. 32).

Table 12 lists the computed CM5, DDEC6, QDR-DDEC6, Hirshfeld, and QTAIM net atomic charges for the sodium chloride crystals illustrated in Fig. 16. This is a challenging test set, because some of these dense crystalline solids are under extremely high pressures. Such extremely high pressures shorten the bond lengths. Because the CM5 correction to the Hirshfeld charges decays exponentially with increasing distance,^75^ for highly compressed bonds the CM5 correction sometimes exceeds chemically reasonable values. For example, as shown in Table 12, the CM5 charge on the sodium atom in some of the compressed sodium chloride crystals exceeded +1.0, which means that some of the Na core electrons were mistakenly assigned to other atoms in the material. Our computational tests as well as prior literature^75,129^ showed the CM5 method often performs well for typical bond lengths and typical coordination numbers, which are close to the conditions for which the CM5 method was parameterized. However, for compressed bond lengths (see Table 12) or unusually high coordination numbers (*e.g.*, Cs@C_60_ molecule in ref. 32), the CM5 method sometimes assigns some of the core electrons to the wrong atom in a material.

**Table 12:** CM5, DDEC6, QDR-DDEC6, Hirshfeld, and QTAIM net atomic charges of sodium chloride crystals. QTAIM results from ref. 32. We computed the CM5, DDEC6, QDR-DDEC6, and Hirshfeld charges from the electron density files generated in ref. 32

Atom type	Number of atoms	CM5^a^	DDEC6^a^	QDR-DDEC6^a^	Hirshfeld^a^	QTAIM^a^
***Cmmm*-Na**_**2**_ **Cl crystal at 180 GPa**						
Na(1)	2	0.693 (0.690)	0.317 (0.338)	0.202 (0.221)	0.107 (0.104)	b
Na(2)	2	0.855 (0.862)	0.546 (0.565)	0.482 (0.499)	0.116 (0.123)	b
Na(3)	4	2.200 (2.167)	0.848 (0.846)	0.935 (0.934)	0.205 (0.172)	b
Cl(1)	4	−2.975 (−2.943)	−1.280 (−1.298)	−1.277 (−1.294)	−0.316 (−0.285)	b

***Cmmm*-Na**_**3**_ **Cl**_**2**_ **crystal at 280 GPa**						
Na(1)	2	1.436 (1.437)	0.953 (0.888)	0.968 (0.906)	0.179 (0.180)	0.780 (0.560)
Na(2)	4	2.492 (2.460)	0.870 (0.872)	0.832 (0.809)	0.235 (0.204)	0.643 (0.291)
Cl(1)	4	−3.210 (−3.179)	−1.346 (−1.316)	−1.316 (−1.262)	−0.325 (−0.294)	−1.033 (−0.571)

***Imma*-Na**_**2**_ **Cl crystal at 300 GPa**						
Na(1)	8	2.180 (2.152)	0.813 (0.790)	0.830 (0.806)	0.205 (0.178)	0.676 (0.317)
Cl(1)	4	−4.359 (−4.305)	−1.625 (−1.580)	−1.660 (−1.612)	−0.410 (−0.356)	−1.351 (−0.633)

***P*4/*m*-Na**_**3**_ **Cl**_**2**_ **crystal at 140 GPa**						
Na(1)	4	1.016 (1.020)	0.803 (0.774)	0.796 (0.764)	0.131 (0.135)	b
Na(2)	1	1.147 (1.150)	0.954 (0.904)	0.954 (0.909)	0.187 (0.190)	b
Na(3)	1	0.400 (0.404)	−0.307 (−0.221)	−0.388 (−0.308)	−0.028 (−0.024)	b
Cl(1)	4	−1.402 (−1.409)	−0.965 (−0.945)	−0.938 (−0.915)	−0.171 (−0.177)	b

***P*4/mmm-Na**_**3**_ **Cl crystal at 140 GPa**						
Na(1)	1	0.224 (0.229)	−0.244 (−0.196)	−0.300 (−0.254)	−0.017 (−0.013)	0.060 (b)
Na(2)	2	0.641 (0.644)	0.475 (0.479)	0.455 (0.456)	0.075 (0.078)	0.531 (b)
Cl(1)	1	−1.506 (−1.516)	−0.706 (−0.761)	−0.609 (−0.658)	−0.133 (−0.143)	−1.122 (b)

***P*4/mmm-Na**_**2**_ **Cl crystal at 120 GPa**						
Na(1)	1	1.084 (1.088)	0.925 (0.889)	0.917 (0.885)	0.184 (0.189)	0.756 (b)
Na(2)	1	0.183 (0.187)	−0.239 (−0.201)	−0.277 (−0.246)	−0.023 (−0.019)	0.041 (b)
Na(3)	2	0.623 (0.625)	0.485 (0.487)	0.474 (0.468)	0.072 (0.074)	0.511 (b)
Cl(1)	2	−1.256 (−1.263)	−0.827 (−0.832)	−0.793 (−0.788)	−0.152 (−0.159)	−0.909 (b)

***Pm*3-NaCl**_**7**_ **crystal at 200 GPa**						
Na(1)	1	1.763 (1.771)	0.896 (0.871)	0.888 (0.863)	0.107 (0.114)	0.883 (0.652)
Cl(1)	1	0.056 (0.056)	0.202 (0.196)	0.255 (0.244)	0.101 (0.101)	0.090 (0.088)
Cl(2)	6	−0.303 (−0.304)	−0.183 (−0.178)	−0.190 (−0.184)	−0.035 (−0.036)	−0.162 (−0.123)

***Pm*3*m*-NaCl crystal at 140 GPa**						
Na(1)	1	1.237 (1.242)	0.964 (0.912)	0.964 (0.912)	0.189 (0.194)	0.862 (0.673)
Cl(1)	1	−1.237 (−1.242)	−0.964 (−0.912)	−0.964 (−0.912)	−0.189 (−0.194)	−0.862 (−0.673)

**NaCl crystal at ambient pressure**						
Na(1)	1	0.425 (0.424)	0.858 (0.847)	0.858 (0.847)	0.210 (0.207)	0.840 (0.829)
Cl(1)	1	−0.425 (−0.424)	−0.858 (−0.847)	−0.858 (−0.847)	−0.210 (−0.207)	−0.840 (−0.829)

***Pm*3*n*-NaCl**_**3**_ **crystal at 200 GPa**						
Na(1)	2	1.780 (1.788)	0.960 (0.907)	0.960 (0.907)	0.138 (0.145)	0.913 (0.653)
Cl(1)	6	−0.593 (−0.596)	−0.320 (−0.302)	−0.320 (−0.302)	−0.046 (−0.048)	−0.304 (−0.218)

***Pnma*-NaCl**_**3**_ **crystal at 40 GPa**						
Na(1)	4	0.735 (0.737)	0.841 (0.850)	0.838 (0.847)	0.114 (0.116)	0.815 (0.770)
Cl(1)	4	−0.446 (−0.447)	−0.589 (−0.595)	−0.579 (−0.586)	−0.151 (−0.152)	−0.530 (−0.501)
Cl(2)	4	−0.046 (−0.046)	0.054 (0.055)	0.054 (0.056)	0.062 (0.062)	−0.030 (−0.028)
Cl(3)	4	−0.244 (−0.245)	−0.306 (−0.310)	−0.312 (−0.317)	−0.026 (−0.027)	−0.255 (−0.242)

a Values listed for 2 frozen Na core electrons; values in parentheses for 10 frozen Na core electrons.

b QTAIM NACs cannot be reported because QTAIM analysis yields more compartments than atoms.

We re-iterate that the QDR-DDEC6 charges are not a chemical correction to the DDEC6 charges, nor are the QDR-DDEC6 charges intended to better represent the chemical states of atoms in materials compared to the DDEC6 charges. Rather, the QDR-DDEC6 charges are intended to improve the electrostatic potential representation by partly resorbing the atom-in-material dipole and quadrupole terms into the point-charge term when the polyatomic multipole expansion is truncated at the monopole order. Previous work showed that among a set of charge assignment methods intended to represent a broad range of chemical and electrostatic properties related to the charges of atoms in materials, the DDEC6 charges were the most representative (*i.e.*, most confluent).^35,36^ That work did not include the QDR-DDEC6 charges which were not yet developed at the time. In Table 11, the QDR-DDEC6 charges have a higher *S*_*α*_ value than the DDEC6 charges, and thus are more confluent amongst that descriptor group, which focuses primarily on charge assignment methods intended to approximately reproduce the electrostatic potential surrounding materials. Hence, we interpret the DDEC6 charges as approximating the chemical states of atoms in materials, while the QDR-DDEC6 charges slightly improve the corresponding atom-centered point-charge models for approximating the electrostatic potential surrounding materials.

## 6. Discussion

Here, we discuss the issues of cloud penetration, polarization, and dipole moments when modeling nonbonded interactions in classical forcefields. Each of these issues have some unusual properties that require clarification.

Ringrose *et al.* previously showed that forcefields using DDEC3, DDEC6, and MBIS nonpolarizable point-charge models without cloud penetration can be optimized to accurately reproduce the liquid densities and heats of vaporization of small organic molecules containing various functional groups.^13^ This does not imply that polarization and/or charge penetration effects are negligible. Rather, it means that the average impact of those effects can be effectively absorbed into the forcefield parameterization protocol. Specifically, the point charges and/or Lennard-Jones parameters can be fine-tuned *via* scaling relationships to effectively absorb the average polarization and/or charge penetration effects by optimizing the forcefield to reproduce experimental liquid densities and heats of vaporization.^13^

Without loss of generality, the electrostatic cloud penetration energy between atom A and atom image b can be conveniently decomposed into the following three contributions:

(1) The neutral electric charge distribution 
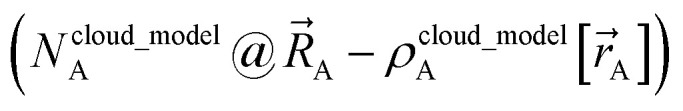
 electrostatically interacting with the atomic point charge 
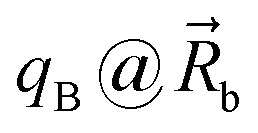
.

(2) The neutral electric charge distribution 
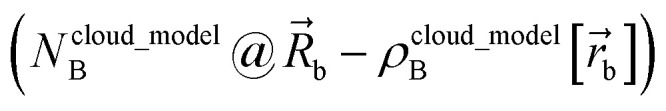
 electrostatically interacting with the atomic point charge 
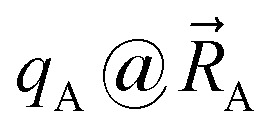
.

(3) The neutral electric charge distribution 
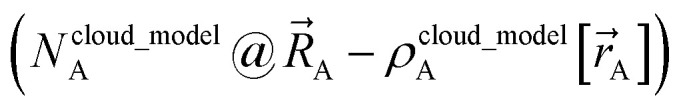
 electrostatically interacting with the neutral electric charge distribution 
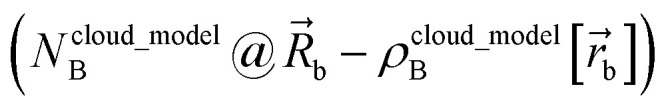
.

Here, 
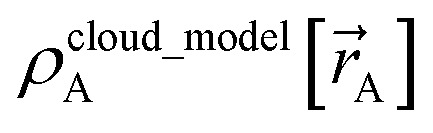
 is any model that approximately reproduces the electron density of atom A in its tail region:91
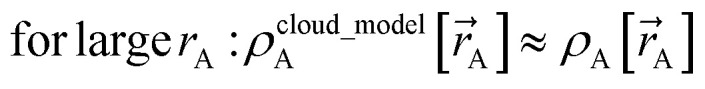
The number of electrons in the cloud model for atom A is92
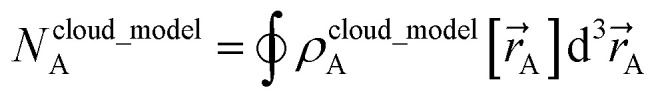
The neutral electric charge distribution 
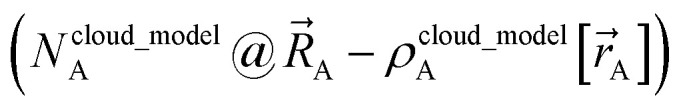
 contains the positive point charge value *N*^cloud_model^_A_ located at the nuclear position of atom A plus the diffuse negatively charged electron cloud 
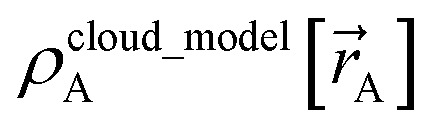
. Here, 
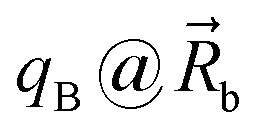
 denotes a point charge of value *q*_B_ located at the nuclear position of atom image b, where *q*_B_ is the net atomic charge assigned by any particular charge assignment method (*e.g.*, DDEC6, MBIS, *etc.*).


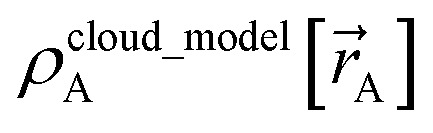
 is typically modeled by the spherically symmetric exponential distribution^14,32^93
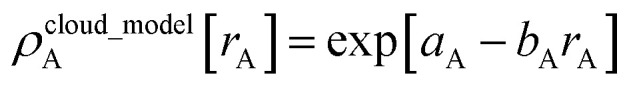


When using DDEC6 atomic population analysis, the electron cloud parameters (*a*_A_, *b*_A_) for atom A are computed by linear regression of ln[*ρ*^avg^_A_[*r*_A_]] over a suitable range of *r*_A_ values in atom A's tail region.^130^ When using MBIS population analysis, *ρ*^cloud_model^_A_[*r*_A_] equals the most diffuse MBIS Slater function (aka the MBIS valence Slater function) located on atom A.^14,19^ Because they have relatively small spatial extents, the MBIS core Slater functions contribute negligibly to the cloud penetration energy.^14,19^

As shown by our computed results for the DDEC6 and MBIS population analysis methods including cloud penetration, the contributions (1) and (2) listed above are negligible at common inter-atomic separation distances. At shorter than equilibrium inter-atomic separation distances, one could damp the electrostatic interaction between *q*_A_ and *q*_B_ to approximate contributions (1) and (2) at short distances.^131^

What our computed results for the DDEC6 and MBIS population analysis methods including cloud penetration did not show is that contribution (3) listed above is negligible at common inter-atomic separation distances. Notably, contribution (3) is the interaction between two neutral electric charge distributions. Here, the term ‘neutral electric charge distribution’ means a charge distribution whose net integrated electric charge is zero.

When parameterizing classical forcefields, the damped dispersion energy, the short-range cloud penetration energy, and the short-range repulsion energy can be combined into an effective dispension model potential (**dispension** = damped **dis**persion + cloud **pen**etration + short-range repul**sion**). The Lennard-Jones potential^132^ is an example of a dispension model potential. More sophisticated (and hopefully more accurate) dispension model potentials can also be conceived.^133^ We are not proposing that cloud penetration effects be neglected when parameterizing classical forcefields, but rather that they be included in the dispension model potential parameterization. This strategy is simpler than more complicated approaches^19,46^ that include a separate cloud penetration potential in the parameterized classical forcefield. This is an important aspect of our proposed forcefield parameterization approach, which is still a work in progress.

We now discuss ways to include polarization effects in electrostatic models used to construct classical forcefields. These polarization effects can be included either explicitly or implicitly.^134^ Please see several reviews^3,26,28,135^ and a special issue^136^ for a thorough discussion of this topic.

Truncated conjugate gradient (TCG) and extended Lagrangian approaches have been developed to provide tractable dynamics of the interacting polarization during MD simulations.^137–139^ One of us introduced the computationally robust and linear-scaling failsafe conjugate residual (FCR) algorithm that readily solves the full polarization equations even for millions of atoms; however, this approach would be computationally expensive since it needs to be performed at every MD timestep.^20^

On the other hand, nonpolarizable forcefields are moderately difficult to accurately parameterize but computationally cheap to use. To obtain reasonably accurate results, nonpolarizable fixed-charge forcefields must be parameterized to include the average polarization effect(s), and many strategies have been developed to do this.^39,140,141^ A typically effective approach is to use atom-centered point-charge values that are approximately halfway between those for a molecule in vacuum and a molecule in the condensed (*e.g.*, liquid) phase.^134,140–142^ MD calculations containing explicit solvent molecules could be performed to simulate the molecule in the condensed (*e.g.*, liquid) phase.^134,140^ Alternatively, one could simulate the molecule using an effective solvent model such as a polarizable continuum model (PCM) that places the molecule in a cavity surrounded by an effective dielectric constant (*e.g.*, *κ* = 4).^39,143^

We prefer a ‘polarlite’ approach to including polarization effects in classical forcefields. This approach incorporates polarization without ‘back polarization’.^144,145^ In other words, the polarizability of each atom interacts with the permanent atomic charges and permanent atomic multiple moments (*e.g.*, permanent atomic dipole moments) of the other atoms (except those in {excluded_A_}) but the induced dipoles do not interact with other induced dipoles.^145,146^ We believe this will make it easier to automate polarlite forcefield parameterization compared to nonpolarizable forcefield parameterization, because explicitly incorporating atomic polarizabilities into the forcefield should allow the gas-phase (vacuum) values of the permanent atom-in-material charges, dipoles, and polarizabilities to be used. Here, {excluded_A_} is the set of atoms (*e.g.*, 1–2, 1–3, and (optionally) 1–4 neighbors) whose nonbonded interactions with atom A are excluded from the parameterized forcefield.

In general, the QM-computed molecular dipole moment includes contributions from both the atom-in-material charges and the atomic dipoles. Some charge assignment methods optimize the atom-in-material charges to ‘strictly’ reproduce the molecular dipole moment, which means the summed contribution to the molecular dipole moment from the atomic dipoles (if any) is exactly zero. The ADCH method studied in this work is an example of such an approach. Constraints can be added to the electrostatic potential fitting methods (*e.g.*, CHELPG, MK, RESP) to ‘strictly’ reproduce the molecular dipole moment;^147^ however, this is not done most of the time. Recently, other methods have been published including ADCHα-I and multipole constrained MBIS. The ADCHα-I method involves an interpolation between Hirshfeld and Iterative Hirshfeld^76^ and optimizes charges to reproduce the molecular dipole moment.^16^ The multipole constrained MBIS method optimizes the assigned atomic charges and/or atomic dipoles to ‘strictly’ reproduce the molecular dipole and/or molecular quadrupole moment.^94^ All of those approaches are subject to some caveats; for example, atom-centered point-charges in a planar molecule cannot exactly reproduce any out-of-plane dipole moment component such as might occur if the molecule is placed in an external electric field. Some initial published tests of those methods gave encouraging results for approximately reproducing molecular electrostatic potentials.^16,94^

One can make the argument that the atom-in-material dipole moment is an important property that should not necessarily be exactly zero even when using a hypothetical ‘perfect’ charge partitioning approach. In Section 5.3 above, the BN nanotube example illustrated the importance of including atomic dipoles in the electrostatic model. In previous work, Richter *et al.* showed that QM-computed infrared (IR) peak intensities for out-of-plane vibrations are precisely reproduced by geometry-dependent atomic charges plus atomic dipoles computed using quantum theory of atoms in molecules (QTAIM^49,50^), Hirshfeld, and DDEC6 methods^147^ (note that this would also be true for MBIS and other stockholder partitioning approaches). They showed these IR peak intensities for out-of-plane vibrations were not approximately reproduced by the ADCH and constrained CHELPG point-charge models for which the atomic charges were optimized to exactly reproduce the molecular dipole moment.^147^

Accordingly, the goals of our QDR scheme are that the atom-in-material dipole moments should be small in magnitude (but not necessarily zero) and that they should be approximately balanced so that the point-charge-only model approximately (albeit not strictly) reproduces the molecular dipole moment. We believe this same goal could be used to design improved stockholder-partitioning schemes. The cMBIS_c-d scheme of ref. 94 does something similar to this, except that it imposes the constraint that the point-charge-only model strictly reproduces the molecular dipole moment.

## 7. Conclusions

Some key advantages of electron density partitioning approaches were mentioned in the Introduction. Electron density partitioning approaches are preferable, because they allow a host of valuable atom-in-material properties to be computed. Among the three stockholder partitioning methods studied here, overall the DDEC6 method performed substantially better than the Hirshfeld method and slightly better than the MBIS method. Hirshfeld charges are typically too small in magnitude.^75–77^

Electrostatic-potential (ESP)-fitted charges typically performed well with relatively low median values of the RMSE and RRMSE. They also exhibited 3rd quartile (*i.e.*, 75% of values) that were relatively low compared to the other approaches. However, occasionally the single-frame ESP-fitted methods yielded gigantic RRMSE values, and this indicates that such approaches are occasionally plagued by over-fitting problems for materials containing buried atoms. In agreement with prior studies, we found that multiframe ESP-fitted charges, which minimize the RMSE simultaneously across multiple geometric conformations of a material, are more robust than single-frame ESP charges optimized for an individual geometry.^29^

ESP-fitted charges require a van der Waals surface enclosing the material's electron density distribution. Consequently, those charge assignment methods do not work for dense nonporous solids under high pressures. As shown in Table 12, well-designed electron-density partitioning methods can assign atomic charges for such materials so that core electrons are properly retained on each corresponding host atom.

The ADCH method includes adjustments to the Hirshfeld atomic charges to reproduce the molecular dipole moment.^86^ The CM5 method includes empirical charge adjustments to the Hirshfeld atomic charges to approximately reproduce reference molecular dipole moments.^75^ Both of these methods performed reasonably well for molecular systems. The CM5 method also performed reasonably well for the nanoporous crystals. The ADCH method was not tested here for the periodic crystals, because it has not yet been implemented in the software programs we used to perform charge analysis for the periodic crystals.

In this work, we introduced a new quadrupole-dipole-resorption (QDR) method that has the following advantages:

(1) The main goal of the QDR method is to adjust the atom-centered point-charge values so that they more accurately reproduce the electrostatic potential surrounding the material. This is done by partly resorbing the atomic dipole and quadrupole moments into the point-charge values. For molecules, this also improves the accuracy of the point-charge model for reproducing the molecule's total dipole and traceless quadrupole moments.

(2) Our QDR method uses stockholder-partitioned net atomic charges, AIM dipole moments, AIM quadrupole moments, and overlap populations as inputs. The AIM dipole and quadrupole moments are partly resorbed using loss functions that minimize deviations between the QDR atomic charges and the stockholder-partitioned AIM charges while also partly resorbing atomic dipoles and/or quadrupoles into the atomic charge values.

(3) Since it is related to an underlying electron-density partitioning method (*e.g.*, DDEC6), this QDR scheme retains access to a host of AIM properties including bond orders, polarizabilities, dispersion coefficients, electron cloud parameters, atomic spin moments, *etc.*

(4) Compared to multiframe ESP-fitting charge assignment schemes, this QDR method is computationally cheaper and has the advantage that it can be applied to individual geometries of a material instead of requiring an ensemble of geometries to compute the atomic charge values. Compared to single-frame ESP-fitting charge assignment schemes, this QDR method exhibits much better conformational transferability of the assigned atomic charge values. Moreover, both the multiframe and single-frame ESP-fitting methods are highly sensitive to the choice of grid points; for example, CHELPG and MK atomic charges may be substantially different for the same molecule.

(5) Our QDR scheme provides a convenient approach to extending electrostatic models for classical forcefields beyond the atom-centered point-charge models. Since it yields the residual atomic dipole and quadrupole moments for each atom in the material, if and where needed the electrostatic model could be conveniently extended by including atom-centered dipole moments and/or offsite charges (*e.g.*, BN nanotube and homodiatomics). Our results showed that in nearly all cases, it would not be necessary to extend the electrostatic model beyond the atomic charges + atomic dipoles order, because the atomic quadrupoles are nearly completely resorbed into the atomic charges + atomic dipoles terms.

(6) Our approach is designed to be used in classical forcefields that include explicit polarization. Since implicitly polarized forcefields rely to some extent on error cancellation, we believe that using explicit polarization could make classical forcefields easier to accurately parameterize.

A key question is whether the current atom-centered point-charge models have reached the best achievable tradeoff between conformational sensitivity and accuracy for reproducing the electrostatic potential surrounding individual conformations of a material. This tradeoff was visualized using the Pareto plots shown in Fig. 15. Amongst current point-charge models, the QDR-DDEC6, CM5, and DDEC6 methods gave the best combination of conformational sensitivity and accuracy for reproducing the electrostatic potential surrounding individual conformations across the nonperiodics dataset.

While the DDEC6 method performed reasonably well across a broad range of material types, the following evidence suggests that it is slightly over-polarizing on average: (i) the DDEC6 point-charge model over-estimated the molecular dipole moments by 9% on average, (ii) the conformationally-averaged DDEC6 atomic charges were 9% larger in rms magnitude than the MF-CHELPG charges and 16% larger than the MF-MK charges, and (iii) the raincloud plots showed that the DDEC6 point charges have a slightly broader distribution of electrostatic potential RMSE and RRMSE values than the CM5 point charges.

On the other hand, the CM5 charges are under-polarized on average. For the nonperiodics dataset, the CM5 method had a rms charge transfer magnitude that was 29% smaller than MF-CHELPG and 25% smaller than MF-MK, and the CM5 point-charge model under-estimated the molecular dipole moments by 4.6% on average. As shown in Table 11, the MBIS charges are more over-polarized than DDEC6 charges. For the nonperiodics dataset, the MBIS method had a rms charge transfer magnitude that was 26% larger than MF-CHELPG and 33% larger than MF-MK. As shown in Table 8, the MBIS point-charge model over-estimated the molecular dipole moments by 16.7% on average.

The QDR-DDEC6 charges fixed most of these issues; however, the rms charge transfer magnitude of the QDR-DDEC6 method was 6% higher than the MF-CHELPG atomic charges and 12% higher than the MF-MK charges. Moreover, the QDR-DDEC6 point-charge models applied to the nanoporous crystals exhibited broader electrostatic RRMSE distributions than the REPEAT and RESP ESP-fitted point-charge models. This strongly indicates that the QDR-DDEC6 point charges are slightly over-polarized (by approximately ∼10%) on average.

In this work, all of the atom-centered point charge models did not perform as well as one might hope for in some materials. In the nonperiodic materials, even the better-performing approaches exhibited some outliers with RRMSE > 1 values for some geometric conformations of some materials. This detailed analysis of many performance metrics indicates that even better-performing charge assignment methods could potentially be developed in future work.

Moreover, one may recommend going beyond atom-centered point-charges by including atom-centered point dipoles (or off-site charges) in the electrostatic model. Atomic multipoles have been included in some prior works such as the AMOEBA forcefield and QTAIM-based forcefields.^3,25,42,51,52^ Off-site charges have been included for some molecules in some prior forcefields.^13,39,66,67,148^ Notably, for some materials (*e.g.*, BN nanotube, homodiatomics, some atom types^149^ in organic compounds, *etc.*) including atomic dipoles (or off-site charges or other multipoles) is required to accurately reproduce the electrostatic potential surrounding the material.

A key advantage of electron-density-partitioning approaches is that they provide a way to improve the electrostatic model by including atomic dipoles. Including permanent atomic dipoles (and/or atomic quadrupoles) in electrostatic models for flexible forcefields is challenging due to their directional orientation.^150^ Since DDEC electron density partitioning has a number of advantages,^31,34,37^ we recommend that future work explore the possibility of developing electrostatic models for flexible forcefields that include QDR-DDEC atom-centered point charges plus atom-centered point dipoles. Tests performed in this work for individual geometric conformations (see Fig. 9, 12, and 15) indicate substantial reductions in the electrostatic RMSE and RRMSE when QDR-DDEC6 atomic dipoles are included. However, future work is required to refine automated atom-in-material dipole orientation across multiple geometric conformations as required for their use in flexible forcefields.

## Author contributions

Both authors selected test systems, planned calculations, performed calculations, analyzed data, prepared tables, prepared graphics, and wrote the manuscript. Both authors developed the new QDR method by performing computational tests during its development. T. A. M. proposed the theory, derived all mathematical equations, wrote the software code to compute the QDR charges, and wrote the manuscript sections related to the QDR approach. A. C. E. prepared all raincloud plots and did most of the job submission and file management. T. A. M. wrote the software codes to compute electrostatic potential RMSE and RRMSE values, and A. C. E. ran these codes to compute the electrostatic potential RMSE and RRMSE values. T. A. M. supervised the work.

## Conflicts of interest

There are no conflicts of interest to declare.

## Supplementary Material

RA-015-D4RA07900K-s001

RA-015-D4RA07900K-s002

RA-015-D4RA07900K-s003

## Data Availability

The ESI† includes supporting data files. An embeddable program containing a subroutine for computing the QDR charges is also provided in the ESI.† Standalone programs for computing the QDR charges and electrostatic potential RMSE and RRMSE are available for download from ddec.sourceforge.net (http://ddec.sourceforge.net).^123^
