# Supplementary material for: How well do various QM-derived net atomic charges reproduce the electrostatic potential surrounding a material across multiple geometric conformations?
Source: RSC Adv. 2025 Jul 7;15(29):23223–65. doi: 10.1039/d4ra07900k (PMC12230677; doi:10.1039/d4ra07900k)
Supplement: RA-015-D4RA07900K-s001 [file RA-015-D4RA07900K-s001.pdf]

Supplementary Information (SI) for RSC Advances.
This journal is © The Royal Society of Chemistry 2025

Electronic Supporting Information for

How well do various QM-derived net atomic charges reproduce the electrostatic potential surrounding a material across multiple geometric conformations?

Alma Carolina Escobosa and Thomas A. Manz*

Department of Chemical & Materials Engineering, New Mexico State University, Las Cruces, New Mexico, 88003-8001. *Corresponding author email: tmanz@nmsu.edu

Contents

1. Proof the QDR scheme preserves the system's total dipole and traceless quadrupole moments

2. Eigenvalues of the coefficients matrix for computing dipole-resorbed charges

3. Eigenvalues of the coefficients matrix for computing quadrupole-resorbed charges

4. Parallelized computational method to compute the QDR charges

5. Supplementary tables

6. Supplementary raincloud plots

S1. Proof the QDR scheme preserves the system's total dipole and traceless quadrupole moments

The total dipole and traceless quadrupole moments are well-defined for systems without any periodic boundary conditions but not for systems with periodic boundary conditions. Hence, the analysis described in this particular section S1 applies only to materials having no periodic boundary conditions. (In contrast, the derivations presented in sections S2, S3, and S4 below are completely general and apply to all materials with or without periodic boundary conditions.)

For a nonperiodic system comprised of atom-centered charges plus atom-centered dipole moments, the total dipole moment is given by

$$\vec{\mu}_{total} = \sum_{A=1}^{Natoms} q_A \vec{R}_A + \sum_{A=1}^{Natoms} \vec{\mu}_A \quad (S1)$$

In eqn (S1), the first summation on the righthand side is the dipole moment due to point charges only.

Since the system has no periodic boundary conditions, the only image 'b' of atom B is the atom B itself. To see if our DR scheme preserves the total dipole moment, we plug in the new atom-centered charges and new atom-centered dipole moments following DR:

$$\vec{\mu}_{total}^{after\_DR} = \sum_{A=1}^{Natoms} q_A^{DR} \vec{R}_A + \sum_{A=1}^{Natoms} \vec{\mu}_A^{DR} \quad (S2)$$

$$q_A^{DR} = q_A + \sum_{B=1}^{Natoms} q_{AB} - \sum_{B=1}^{Natoms} q_{BA} \quad (S3)$$

$$\vec{\mu}_A^{DR} = \vec{\mu}_A + \frac{1}{2}\sum_{B=1}^{Natoms} q_{AB}\vec{R}_{AB} - \frac{1}{2}\sum_{B=1}^{Natoms} q_{BA}\vec{R}_{AB} \quad (S4)$$

where $q_{AA} = 0$. Substituting eqn (S3) and (S4) into (S2) gives

$$\vec{\mu}_{total}^{after\_DR} = \left(\sum_{A=1}^{Natoms} q_A \vec{R}_A + \sum_{A=1}^{Natoms} \vec{\mu}_A\right) + \left(\sum_{A=1}^{Natoms}\sum_{B=1}^{Natoms} \left(q_{AB} - q_{BA}\right)\left(\vec{R}_A + ½\vec{R}_{AB}\right)\right) \quad (S5)$$

Substituting

$$\vec{R}_{AB} = \vec{R}_B - \vec{R}_A \quad (S6)$$

and eqn (S1) gives

$$\vec{\mu}_{total}^{after\_DR} = \vec{\mu}_{total} + \frac{1}{2}\sum_{A=1}^{Natoms}\sum_{B=1}^{Natoms} \left(q_{AB} - q_{BA}\right)\left(\vec{R}_A + \vec{R}_B\right) \quad (S7)$$

The terms (A = i, B = j) and (A = j, B = i) cancel each other, because $(q_{ij} - q_{ji}) = -(q_{ji} - q_{ij})$; hence,

$$\sum_{A=1}^{Natoms}\sum_{B=1}^{Natoms} \left(q_{AB} - q_{BA}\right)\left(\vec{R}_A + \vec{R}_B\right) = 0 \quad (S8)$$

Substituting eqn (S8) into (S7) yields

$$\vec{\mu}_{total}^{after\_DR} = \vec{\mu}_{total} \quad (S9)$$

This proves our DR scheme preserves the total dipole moment.

To see if our QR scheme preserves the total dipole moment, we plug in the new atom-centered charges and new atom-centered dipole moments following QR:

$$\vec{\mu}_{total}^{after\_QR} = \sum_{A=1}^{Natoms} q_A^{QR} \vec{R}_A + \sum_{A=1}^{Natoms} \vec{\mu}_A^{QR} \quad (S10)$$

$$q_A^{QR} = q_A + \sum_{B=1}^{Natoms} q_{AB} - \sum_{B=1}^{Natoms} q_{BA} \quad (S11)$$

$$\vec{\mu}_A^{QR} = \vec{\mu}_A + \sum_{B=1}^{Natoms} q_{AB}\vec{R}_{AB} \quad (S12)$$

where $q_{AA} = 0$. Substituting eqn (S11) and (S12) into (S10) gives

$$\vec{\mu}_{total}^{after\_QR} = \left(\sum_{A=1}^{Natoms} q_A \vec{R}_A + \sum_{A=1}^{Natoms} \vec{\mu}_A \right) + \left(\sum_{A=1}^{Natoms} \sum_{B=1}^{Natoms} \left((q_{AB} - q_{BA}) \vec{R}_A + q_{AB} \vec{R}_{AB} \right) \right) \quad \text{(S13)}$$

With the help of eqn (S6), we expand

$$(q_{AB} - q_{BA}) \vec{R}_A + q_{AB} \vec{R}_{AB} = (q_{AB} - q_{BA}) \vec{R}_A + q_{AB} \left(\vec{R}_B - \vec{R}_A \right) = q_{AB} \vec{R}_B - q_{BA} \vec{R}_A \quad \text{(S14)}$$

Substituting eqn (S1) and (S14) into (S13) gives

$$\vec{\mu}_{total}^{after\_QR} = \vec{\mu}_{total} + \sum_{A=1}^{Natoms} \sum_{B=1}^{Natoms} \left(q_{AB} \vec{R}_B - q_{BA} \vec{R}_A \right) \quad \text{(S15)}$$

The terms (A = i, B = j) and (A = j, B = i) cancel each other, because

$$\left(q_{ij} \vec{R}_j - q_{ji} \vec{R}_i \right) = -\left(q_{ji} \vec{R}_i - q_{ij} \vec{R}_j \right) \quad \text{(S16)}$$

Hence,

$$\sum_{A=1}^{Natoms} \sum_{B=1}^{Natoms} \left(q_{AB} \vec{R}_B - q_{BA} \vec{R}_A \right) = 0 \quad \text{(S17)}$$

Substituting eqn (S17) into (S15) yields

$$\vec{\mu}_{total}^{after\_QR} = \vec{\mu}_{total} \quad \text{(S18)}$$

This proves our QR scheme preserves the total dipole moment.

For a system comprised of atom-centered charges, atom-centered dipole moments, and atom-centered quadrupole moments, the unit cell's overall traceless quadrupole moment tensor is given by

$$\vec{\vec{Q}}_{overall} = \sum_{A=1}^{Natoms} q_A \left(\vec{R}_A \vec{R}_A - (R_A)^2 \vec{\vec{\delta}}/3 \right) + \sum_{A=1}^{Natoms} \left(\vec{\mu}_A \vec{R}_A + \vec{R}_A \vec{\mu}_A - (2/3) \left(\vec{\mu}_A \bullet \vec{R}_A \right) \vec{\vec{\delta}} \right) + \sum_{A=1}^{Natoms} \vec{\vec{Q}}_A \quad \text{(S19)}$$

In eqn (S19), the first summation on the righthand side is the total traceless quadrupole moment due to point charges only. Including both the first and second summations on the righthand side of eqn (S19)—but excluding the third summation—yields the total traceless quadrupole moment due to the point charges and atom-centered dipole moments.

To see if our DR scheme preserves the overall traceless quadrupole moment tensor, we plug in the new atom-centered charges, new atom-centered dipole moments, and unchanged atom-centered quadrupole moments following DR:

$$\vec{\vec{Q}}_{overall}^{after\_DR} = \sum_{A=1}^{Natoms} q_A^{DR} \left(\vec{R}_A \vec{R}_A - (R_A)^2 \vec{\vec{\delta}}/3 \right) + \sum_{A=1}^{Natoms} \left(\vec{\mu}_A^{DR} \vec{R}_A + \vec{R}_A \vec{\mu}_A^{DR} - (2/3) \left(\vec{\mu}_A^{DR} \bullet \vec{R}_A \right) \vec{\vec{\delta}} \right) + \sum_{A=1}^{Natoms} \vec{\vec{Q}}_A \quad \text{(S20)}$$

With the help of eqn (S3), the first summation in eqn (S20) expands as

$$\sum_{A=1}^{Natoms} q_A^{DR} \left(\vec{R}_A \vec{R}_A - (R_A)^2 \vec{\vec{\delta}}/3 \right) = \sum_{A=1}^{Natoms} q_A \left(\vec{R}_A \vec{R}_A - (R_A)^2 \vec{\vec{\delta}}/3 \right) + \sum_{A=1}^{Natoms} \sum_{B=1}^{Natoms} (q_{AB} - q_{BA}) \left(\vec{R}_A \vec{R}_A - (R_A)^2 \vec{\vec{\delta}}/3 \right) \quad \text{(S21)}$$

With the help of eqn (S4), the second summation in eqn (S20) expands as

$$\begin{aligned} \sum_{A=1}^{Natoms} \left(\vec{\mu}_A^{DR} \vec{R}_A + \vec{R}_A \vec{\mu}_A^{DR} - (2/3) \left(\vec{\mu}_A^{DR} \bullet \vec{R}_A \right) \vec{\vec{\delta}} \right) &= \sum_{A=1}^{Natoms} \left(\vec{\mu}_A \vec{R}_A + \vec{R}_A \vec{\mu}_A - (2/3) \left(\vec{\mu}_A \bullet \vec{R}_A \right) \vec{\vec{\delta}} \right) \\ &+ \frac{1}{2} \sum_{A=1}^{Natoms} \sum_{B=1}^{Natoms} (q_{AB} - q_{BA}) \left(\vec{R}_{AB} \vec{R}_A + \vec{R}_A \vec{R}_{AB} - (2/3) \left(\vec{R}_{AB} \bullet \vec{R}_A \right) \vec{\vec{\delta}} \right) \end{aligned} \quad \text{(S22)}$$

Using eqn (S6), the last term in eqn (S22) expands as

$$\begin{aligned} \frac{1}{2} \sum_{A=1}^{Natoms} \sum_{B=1}^{Natoms} (q_{AB} - q_{BA}) \left(\vec{R}_{AB} \vec{R}_A + \vec{R}_A \vec{R}_{AB} - (2/3) \left(\vec{R}_{AB} \bullet \vec{R}_A \right) \vec{\vec{\delta}} \right) &= +\frac{1}{2} \sum_{A=1}^{Natoms} \sum_{B=1}^{Natoms} (q_{AB} - q_{BA}) \left(\vec{R}_B \vec{R}_A + \vec{R}_A \vec{R}_B - (2/3) \left(\vec{R}_B \bullet \vec{R}_A \right) \vec{\vec{\delta}} \right) \\ &- \sum_{A=1}^{Natoms} \sum_{B=1}^{Natoms} (q_{AB} - q_{BA}) \left(\vec{R}_A \vec{R}_A - (R_A)^2 \vec{\vec{\delta}}/3 \right) \end{aligned}$$

(S23)

Substituting eqn (S23) into (S22) gives

$$\sum_{A=1}^{Natoms}\left(\vec{\mu}_A^{DR}\vec{R}_A+\vec{R}_A\vec{\mu}_A^{DR}-(2/3)\left(\vec{\mu}_A^{DR}\bullet\vec{R}_A\right)\vec{\vec{\delta}}\right)=\sum_{A=1}^{Natoms}\left(\vec{\mu}_A\vec{R}_A+\vec{R}_A\vec{\mu}_A-(2/3)\left(\vec{\mu}_A\bullet\vec{R}_A\right)\vec{\vec{\delta}}\right)$$
$$+\frac{1}{2}\sum_{A=1}^{Natoms}\sum_{B=1}^{Natoms}\left(q_{AB}-q_{BA}\right)\left(\vec{R}_B\vec{R}_A+\vec{R}_A\vec{R}_B-(2/3)\left(\vec{R}_B\bullet\vec{R}_A\right)\vec{\vec{\delta}}\right)-\sum_{A=1}^{Natoms}\sum_{B=1}^{Natoms}\left(q_{AB}-q_{BA}\right)\left(\vec{R}_A\vec{R}_A-\left(R_A\right)^2\vec{\vec{\delta}}/3\right) \quad \text{(S24)}$$

Substituting eqn (S24) and (S21) into (S20) and using eqn (S19) gives

$$\vec{\vec{Q}}_{overall}^{after\_DR}=\vec{\vec{Q}}_{overall}+\frac{1}{2}\sum_{A=1}^{Natoms}\sum_{B=1}^{Natoms}\left(q_{AB}-q_{BA}\right)\left(\vec{R}_B\vec{R}_A+\vec{R}_A\vec{R}_B-(2/3)\left(\vec{R}_A\bullet\vec{R}_B\right)\vec{\vec{\delta}}\right) \quad \text{(S25)}$$

The terms (A = i, B = j) and (A = j, B = i) cancel each other, because $(q_{ij} – q_{ji}) = -(q_{ji} – q_{ij})$; hence,

$$\sum_{A=1}^{Natoms}\sum_{B=1}^{Natoms}\left(q_{AB}-q_{BA}\right)\left(\vec{R}_B\vec{R}_A+\vec{R}_A\vec{R}_B-(2/3)\left(\vec{R}_A\bullet\vec{R}_B\right)\vec{\vec{\delta}}\right)=0 \quad \text{(S26)}$$

Substituting eqn (S26) into (S25) yields

$$\vec{\vec{Q}}_{overall}^{after\_DR}=\vec{\vec{Q}}_{overall} \quad \text{(S27)}$$

This proves our DR scheme preserves the overall traceless quadrupole moment tensor.

To see if our QR scheme preserves the overall traceless quadrupole moment tensor, we plug in the new atom-centered charges, new atom-centered dipole moments, and new atom-centered quadrupole moments following QR:

$$\vec{\vec{Q}}_{overall}^{after\_QR}=\sum_{A=1}^{Natoms}q_A^{QR}\left(\vec{R}_A\vec{R}_A-\left(R_A\right)^2\vec{\vec{\delta}}/3\right)+\sum_{A=1}^{Natoms}\left(\vec{\mu}_A^{QR}\vec{R}_A+\vec{R}_A\vec{\mu}_A^{QR}-(2/3)\left(\vec{\mu}_A^{QR}\bullet\vec{R}_A\right)\vec{\vec{\delta}}\right)+\sum_{A=1}^{Natoms}\vec{\vec{Q}}_A^{QR} \quad \text{(S28)}$$

With the help of eqn (S11), the first summation in eqn (S28) expands as

$$\sum_{A=1}^{Natoms}q_A^{QR}\left(\vec{R}_A\vec{R}_A-\left(R_A\right)^2\vec{\vec{\delta}}/3\right)=\sum_{A=1}^{Natoms}q_A\left(\vec{R}_A\vec{R}_A-\left(R_A\right)^2\vec{\vec{\delta}}/3\right)+\sum_{A=1}^{Natoms}\sum_{B=1}^{Natoms}\left(q_{AB}-q_{BA}\right)\left(\vec{R}_A\vec{R}_A-\left(R_A\right)^2\vec{\vec{\delta}}/3\right) \quad \text{(S29)}$$

With the help of eqn (S12), the second summation in eqn (S28) expands as

$$\sum_{A=1}^{Natoms}\left(\vec{\mu}_A^{QR}\vec{R}_A+\vec{R}_A\vec{\mu}_A^{QR}-(2/3)\left(\vec{\mu}_A^{QR}\bullet\vec{R}_A\right)\vec{\vec{\delta}}\right)=\sum_{A=1}^{Natoms}\left(\vec{\mu}_A\vec{R}_A+\vec{R}_A\vec{\mu}_A-(2/3)\left(\vec{\mu}_A\bullet\vec{R}_A\right)\vec{\vec{\delta}}\right)$$
$$+\sum_{A=1}^{Natoms}\sum_{B=1}^{Natoms}q_{AB}\left(\vec{R}_{AB}\vec{R}_A+\vec{R}_A\vec{R}_{AB}-(2/3)\left(\vec{R}_{AB}\bullet\vec{R}_A\right)\vec{\vec{\delta}}\right) \quad \text{(S30)}$$

The atomic quadrupole moments following QR are

$$\vec{\vec{Q}}_A^{QR}=\vec{\vec{Q}}_A+\sum_{B=1}^{Natoms}q_{AB}\left(\vec{R}_{AB}\vec{R}_{AB}-\left(R_{AB}\right)^2\vec{\vec{\delta}}/3\right) \quad \text{(S31)}$$

Hence, the last summation in eqn (S28) expands as

$$\sum_{A=1}^{Natoms}\vec{\vec{Q}}_A^{QR}=\sum_{A=1}^{Natoms}\vec{\vec{Q}}_A+\sum_{A=1}^{Natoms}\sum_{B=1}^{Natoms}q_{AB}\left(\vec{R}_{AB}\vec{R}_{AB}-\left(R_{AB}\right)^2\vec{\vec{\delta}}/3\right) \quad \text{(S32)}$$

Substituting eqn (S29), (S30), and (S32) into (S28) and using eqn (S19) gives

$$\vec{\vec{Q}}_{overall}^{after\_QR}=\vec{\vec{Q}}_{overall}+\sum_{A=1}^{Natoms}\sum_{B=1}^{Natoms}\left(q_{AB}-q_{BA}\right)\left(\vec{R}_A\vec{R}_A-\left(R_A\right)^2\vec{\vec{\delta}}/3\right)$$
$$+\sum_{A=1}^{Natoms}\sum_{B=1}^{Natoms}q_{AB}\left(\vec{R}_{AB}\vec{R}_A+\vec{R}_A\vec{R}_{AB}-(2/3)\left(\vec{R}_{AB}\bullet\vec{R}_A\right)\vec{\vec{\delta}}\right)+\sum_{A=1}^{Natoms}\sum_{B=1}^{Natoms}q_{AB}\left(\vec{R}_{AB}\vec{R}_{AB}-\left(R_{AB}\right)^2\vec{\vec{\delta}}/3\right) \quad \text{(S33)}$$

The first summation in eqn (S33) can be rewritten as

$$\sum_{A=1}^{Natoms}\sum_{B=1}^{Natoms}\left(q_{AB}-q_{BA}\right)\left(\vec{R}_A\vec{R}_A-\left(R_A\right)^2\vec{\vec{\delta}}/3\right)=\sum_{A=1}^{Natoms}\sum_{B=1}^{Natoms}q_{AB}\left(\vec{R}_A\vec{R}_A-\left(R_A\right)^2\vec{\vec{\delta}}/3\right)-\sum_{A=1}^{Natoms}\sum_{B=1}^{Natoms}q_{AB}\left(\vec{R}_B\vec{R}_B-\left(R_B\right)^2\vec{\vec{\delta}}/3\right) \quad \text{(S34)}$$

Substituting eqn (S34) into (S33) and collecting terms yields

$$\vec{\vec{Q}}_{\text{overall}}^{\text{after\_QR}} = \vec{\vec{Q}}_{\text{overall}} + \sum_{A=1}^{\text{Natoms}}\sum_{B=1}^{\text{Natoms}} q_{AB}\left((R_B)^2 - (R_A)^2 - 2\left(\vec{R}_{AB}\bullet\vec{R}_A\right) - (R_{AB})^2\right)\vec{\vec{\delta}}/3 + \sum_{A=1}^{\text{Natoms}}\sum_{B=1}^{\text{Natoms}} q_{AB}\left(\vec{R}_A\vec{R}_A - \vec{R}_B\vec{R}_B + \vec{R}_{AB}\vec{R}_A + \vec{R}_A\vec{R}_{AB} + \vec{R}_{AB}\vec{R}_{AB}\right) \quad \text{(S35)}$$

Note that

$$(R_{AB})^2 = \left(\vec{R}_B - \vec{R}_A\right)\bullet\left(\vec{R}_B - \vec{R}_A\right) = (R_B)^2 - 2\left(\vec{R}_A\bullet\vec{R}_B\right) + (R_A)^2 \quad \text{(S36)}$$

$$\vec{R}_{AB}\bullet\vec{R}_A = \vec{R}_A\bullet\vec{R}_{AB} = \vec{R}_A\bullet\left(\vec{R}_B - \vec{R}_A\right) = \left(\vec{R}_A\bullet\vec{R}_B\right) - (R_A)^2 \quad \text{(S37)}$$

This allows to rewrite

$$(R_B)^2 - (R_A)^2 - 2\left(\vec{R}_A\bullet\vec{R}_{AB}\right) - (R_{AB})^2 = (R_B)^2 - (R_A)^2 - 2\left(\left(\vec{R}_A\bullet\vec{R}_B\right) - (R_A)^2\right) - \left((R_B)^2 - 2\left(\vec{R}_A\bullet\vec{R}_B\right) + (R_A)^2\right) = 0 \quad \text{(S38)}$$

Note that

$$\vec{R}_{AB}\vec{R}_A + \vec{R}_A\vec{R}_{AB} = \left(\vec{R}_B - \vec{R}_A\right)\vec{R}_A + \vec{R}_A\left(\vec{R}_B - \vec{R}_A\right) = \vec{R}_B\vec{R}_A + \vec{R}_A\vec{R}_B - 2\vec{R}_A\vec{R}_A \quad \text{(S39)}$$

$$\vec{R}_{AB}\vec{R}_{AB} = \left(\vec{R}_B - \vec{R}_A\right)\left(\vec{R}_B - \vec{R}_A\right) = \vec{R}_B\vec{R}_B - \vec{R}_A\vec{R}_B - \vec{R}_B\vec{R}_A + \vec{R}_A\vec{R}_A \quad \text{(S40)}$$

This allows to rewrite

$$\vec{R}_A\vec{R}_A - \vec{R}_B\vec{R}_B + \left(\vec{R}_{AB}\vec{R}_A + \vec{R}_A\vec{R}_{AB}\right) + \vec{R}_{AB}\vec{R}_{AB} = \vec{R}_A\vec{R}_A - \vec{R}_B\vec{R}_B + \left(\vec{R}_B\vec{R}_A + \vec{R}_A\vec{R}_B - 2\vec{R}_A\vec{R}_A\right) + \left(\vec{R}_B\vec{R}_B - \vec{R}_A\vec{R}_B - \vec{R}_B\vec{R}_A + \vec{R}_A\vec{R}_A\right) = 0 \quad \text{(S41)}$$

Substituting eqn (S38) and (S41) into (S35) yields

$$\vec{\vec{Q}}_{\text{overall}}^{\text{after\_QR}} = \vec{\vec{Q}}_{\text{overall}} \quad \text{(S42)}$$

This proves our QR scheme preserves the overall traceless quadrupole moment tensor.

S2. Eigenvalues of the coefficients matrix for computing dipole-resorbed charges

The condition number of a normal matrix is defined as its largest singular value (absolute value of eigenvalue) to its smallest singular value (absolute value of eigenvalue):

$$\kappa\left[M^{(A)}\right] = \frac{|\lambda|_{\max}}{|\lambda|_{\min}} \quad \text{(S43)}$$

Manifestly, for any real-valued column vector having unit length:

$$\sum_b\left(U_b^{(A)}\right)^2 = 1 \quad \text{(S44)}$$

its inner product with a real-valued symmetric matrix $M^{(A)}$ can have a resulting value anywhere between the smallest and largest eigenvalues of matrix $M^{(A)}$:

$$\lambda_{\min} \le \left(U^{(A)}\right)^T M^{(A)} U^{(A)} \le \lambda_{\max} \quad \text{(S45)}$$

Defining

$$\vec{\Lambda}^{(A)} = \sum_b\left(U_b^{(A)}\left(2\tanh\left[2OP_{Ab}\right]\right)\hat{R}_{Ab}\right) \quad \text{(S46)}$$

and using the definition of matrix M for dipole resorption, we have

$$\left(U^{(A)}\right)^T M^{(A)} U^{(A)} = 1 + \vec{\Lambda}^{(A)}\bullet\vec{\Lambda}^{(A)} \quad \text{(S47)}$$

Since $U^{(A)}$ is a column vector of unit length and $\hat{R}_{Ab}$ is a spatial unit vector, it directly follows from eqn (S46) that

$$0 \le \vec{\Lambda}^{(A)}\bullet\vec{\Lambda}^{(A)} \le \left(\sum_b\left(2\tanh\left[2OP_{Ab}\right]\right)\right)^2 \quad \text{(S48)}$$

Combining eqn (S45), (S47), and (S48) shows that

$$1 \le \lambda_{min} \le \lambda_i \le \lambda_{max} \le \left(1 + \left(\sum_b \left(2\tanh\left[2OP_{Ab}\right]\right)\right)^2\right) \quad (S49)$$

Inserting eqn (S49) into (S43) shows that the condition number is

$$1 \le \kappa\left[M^{(A)}\right] \le \left(1 + \left(\sum_b \left(2\tanh\left[2OP_{Ab}\right]\right)\right)^2\right) \quad (S50)$$

Since

$$\tanh\left[s \ge 0\right] \le s \quad (S51)$$

it directly follows that

$$\sum_b \left(2\tanh\left[2OP_{Ab}\right]\right) \le 4SOP_A \quad (S52)$$

SOP_A is the sum of overlap populations for atom A. Combining eqn (S50) and (S52) gives

$$1 \le \kappa\left[M^{(A)}\right] \le \left(1 + 16\left(SOP_A\right)^2\right) \quad (S53)$$

Since SOP_A is never a huge number, this proves matrix $M^{(A)}$ can never have a huge condition number (i.e., it is always well-conditioned).

The matrix $M^{(A)}$ has at most four distinct eigenvalues; that is, the number of distinct eigenvalues of matrix $M^{(A)}$ is 1, 2, 3, or 4. *Proof:* (1) Careful examination of the definition of matrix $M^{(A)}$ described in this article's main text shows the matrix $M^{(A)}$ can be expanded as

$$M^{(A)} = I + \alpha\alpha^T + \beta\beta^T + \gamma\gamma^T \quad (S54)$$

where I is the identity matrix and α, β, and γ are the following column vectors:

$$\alpha_b^{(A)} = \left(2\tanh\left[2OP_{Ab}\right]\right)\left(\hat{R}_{Ab}\right)_x \quad (S55)$$

$$\beta_b^{(A)} = \left(2\tanh\left[2OP_{Ab}\right]\right)\left(\hat{R}_{Ab}\right)_y \quad (S56)$$

$$\gamma_b^{(A)} = \left(2\tanh\left[2OP_{Ab}\right]\right)\left(\hat{R}_{Ab}\right)_z \quad (S57)$$

Here, $\left(\hat{R}_{Ab}\right)_x$, $\left(\hat{R}_{Ab}\right)_y$, and $\left(\hat{R}_{Ab}\right)_z$ are the x, y, and z components of the unit vector $\hat{R}_{Ab}$. (2) Manifestly, the dimension of the space spanned by $\alpha\alpha^T + \beta\beta^T + \gamma\gamma^T$ (i.e., its rank) equals the number of linearly independent column vectors in the set $\{\alpha,\beta,\gamma\}$. Clearly, a set of three column vectors contains at most three linearly-independent column vectors. Let $\eta \le 3$ be the number of linearly independent column vectors in the set $\{\alpha,\beta,\gamma\}$. Then, the space spanned by $\alpha\alpha^T + \beta\beta^T + \gamma\gamma^T$ is η-dimensional and has η linearly-independent eigenvectors with non-zero eigenvalues. (3) As well-known from linear algebra, adding a multiple of the identity matrix performs an eigenvalue shift. That is, each and every eigenvalue of matrix $M^{(A)}$ (see eqn (S54)) has a value equal to 1.0 plus the corresponding eigenvalue of $\alpha\alpha^T + \beta\beta^T + \gamma\gamma^T$. (4) Since $\alpha\alpha^T + \beta\beta^T + \gamma\gamma^T$ has $\eta \le 3$ nonzero eigenvalues, it directly follows from (3) that $\eta \le 3$ eigenvalues of matrix $M^{(A)}$ are not equal to one and all other eigenvalues of matrix $M^{(A)}$ equal one. Manifestly, this means matrix $M^{(A)}$ has 1, 2, 3, or 4 distinct eigenvalues.

S3. Eigenvalues of the coefficients matrix for computing quadrupole-resorbed charges

The condition number of a normal matrix is defined as its largest singular value (absolute value of eigenvalue) to its smallest singular value (absolute value of eigenvalue):

$$\kappa\left[C^{(A)}\right] = \frac{|\lambda|_{max}}{|\lambda|_{min}} \tag{S58}$$

Manifestly, for any real-valued column vector having unit length:

$$\sum_b \left(U_b^{(A)}\right)^2 = 1 \tag{S59}$$

its inner product with a real-valued symmetric matrix $C^{(A)}$ can have a resulting value anywhere between the smallest and largest eigenvalues of matrix $C^{(A)}$:

$$\lambda_{min} \leq \left(U^{(A)}\right)^T C^{(A)} U^{(A)} \leq \lambda_{max} \tag{S60}$$

Defining

$$\vec{\vec{\Omega}}^{(A)} = \sum_b \left(U_b^{(A)} \left(2\tanh[2OP_{Ab}]\right) \left(\hat{R}_{Ab}\hat{R}_{Ab} - \vec{\vec{\delta}}/3\right)\right) \tag{S61}$$

and using the definition of matrix $C^{(A)}$ for quadrupole resorption, we have

$$\left(U^{(A)}\right)^T C^{(A)} U^{(A)} = 1 + \vec{\vec{\Omega}}^{(A)} : \vec{\vec{\Omega}}^{(A)} \tag{S62}$$

where ‘:’ is the double dot product. Since

$$-(1/3) \leq \left(\hat{R}_{Ab}\hat{R}_{Ab} - \vec{\vec{\delta}}/3\right) : \left(\hat{R}_{Ad}\hat{R}_{Ad} - \vec{\vec{\delta}}/3\right) = \left(\left(\hat{R}_{Ab} \bullet \hat{R}_{Ad}\right)^2 - 1/3\right) \leq (2/3) \tag{S63}$$

and $U^{(A)}$ is a column vector of unit length, it directly follows from eqn (S61) that

$$0 \leq \vec{\vec{\Omega}}^{(A)} : \vec{\vec{\Omega}}^{(A)} \leq \frac{2}{3}\left(\sum_b \left(2\tanh[2OP_{Ab}]\right)\right)^2 \tag{S64}$$

Combining eqn (S60), (S62), and (S64) shows that

$$1 \leq \lambda_{min} \leq \lambda_i \leq \lambda_{max} \leq \left(1 + \frac{2}{3}\left(\sum_b \left(2\tanh[2OP_{Ab}]\right)\right)^2\right) \tag{S65}$$

Inserting eqn (S65) into (S58) shows that the condition number is

$$1 \leq \kappa\left[C^{(A)}\right] \leq \left(1 + \frac{2}{3}\left(\sum_b \left(2\tanh[2OP_{Ab}]\right)\right)^2\right) \tag{S66}$$

Inserting eqn (S52) into (S66) gives

$$1 \leq \kappa\left[C^{(A)}\right] \leq \left(1 + \frac{32}{3}\left(SOP_A\right)^2\right) \tag{S67}$$

Since SOP_A is never a huge number, this proves matrix $C^{(A)}$ can never have a huge condition number (i.e., it is always well-conditioned).

The matrix $C^{(A)}$ has at most eight distinct eigenvalues; that is, the number of distinct eigenvalues of matrix $C^{(A)}$ is 1, 2, 3, 4, 5, 6, 7, or 8. *Proof:* (1) Careful examination of the definition of matrix $C^{(A)}$ described in this article’s main text shows the matrix $C^{(A)}$ can be expanded as

$$C^{(A)} = I + (-1/3)\chi\chi^T + \nu\nu^T + 2\varpi\varpi^T + 2\vartheta\vartheta^T + \varsigma\varsigma^T + 2\omega\omega^T + \xi\xi^T \tag{S68}$$

where I is the identity matrix and χ, ν, ϖ, ϑ, ς, ω, and ξ are the following column vectors:

$$\chi_b^{(A)} = 2\tanh[2OP_{Ab}] \tag{S69}$$

$$\nu_b^{(A)} = \left(2\tanh[2OP_{Ab}]\right)\left(\hat{R}_{Ab}\right)_x \left(\hat{R}_{Ab}\right)_x \tag{S70}$$

$$\varpi_b^{(A)} = \left(2\tanh\left[2OP_{Ab}\right]\right)\left(\hat{R}_{Ab}\right)_x\left(\hat{R}_{Ab}\right)_y \tag{S71}$$

$$\vartheta_b^{(A)} = \left(2\tanh\left[2OP_{Ab}\right]\right)\left(\hat{R}_{Ab}\right)_x\left(\hat{R}_{Ab}\right)_z \tag{S72}$$

$$\varsigma_b^{(A)} = \left(2\tanh\left[2OP_{Ab}\right]\right)\left(\hat{R}_{Ab}\right)_y\left(\hat{R}_{Ab}\right)_y \tag{S73}$$

$$\omega_b^{(A)} = \left(2\tanh\left[2OP_{Ab}\right]\right)\left(\hat{R}_{Ab}\right)_y\left(\hat{R}_{Ab}\right)_z \tag{S74}$$

$$\xi_b^{(A)} = \left(2\tanh\left[2OP_{Ab}\right]\right)\left(\hat{R}_{Ab}\right)_z\left(\hat{R}_{Ab}\right)_z \tag{S75}$$

Here, $\left(\hat{R}_{Ab}\right)_x$, $\left(\hat{R}_{Ab}\right)_y$, and $\left(\hat{R}_{Ab}\right)_z$ are the x, y, and z components of the unit vector $\hat{R}_{Ab}$. (2) Manifestly, the dimension of the space spanned by $(-1/3)\chi\chi^T + \nu\nu^T + 2\varpi\varpi^T + 2\vartheta\vartheta^T + \varsigma\varsigma^T + 2\omega\omega^T + \xi\xi^T$ (i.e., its rank) equals the number of linearly independent column vectors in the set $\{\chi, \nu, \varpi, \vartheta, \varsigma, \omega, \xi\}$. Clearly, a set of seven column vectors contains at most seven linearly-independent column vectors. Let $\tau \le 7$ be the number of linearly independent column vectors in the set $\{\chi, \nu, \varpi, \vartheta, \varsigma, \omega, \xi\}$. Then, the space spanned by $(-1/3)\chi\chi^T + \nu\nu^T + 2\varpi\varpi^T + 2\vartheta\vartheta^T + \varsigma\varsigma^T + 2\omega\omega^T + \xi\xi^T$ is τ-dimensional and has τ linearly-independent eigenvectors with non-zero eigenvalues. (3) As well-known from linear algebra, adding a multiple of the identity matrix performs an eigenvalue shift. That is, each and every eigenvalue of matrix $C^{(A)}$ (see eqn (S68)) has a value equal to 1.0 plus the corresponding eigenvalue of $(-1/3)\chi\chi^T + \nu\nu^T + 2\varpi\varpi^T + 2\vartheta\vartheta^T + \varsigma\varsigma^T + 2\omega\omega^T + \xi\xi^T$. (4) Since $(-1/3)\chi\chi^T + \nu\nu^T + 2\varpi\varpi^T + 2\vartheta\vartheta^T + \varsigma\varsigma^T + 2\omega\omega^T + \xi\xi^T$ has $\tau \le 7$ nonzero eigenvalues, it directly follows from (3) that $\tau \le 7$ eigenvalues of matrix $C^{(A)}$ are not equal to one and all other eigenvalues of matrix $C^{(A)}$ equal one. Manifestly, this means matrix $C^{(A)}$ has 1, 2, 3, 4, 5, 6, 7, or 8 distinct eigenvalues.

S4. Parallelized computational method to compute the QDR charges

Let $\aleph$ be the number of distinct eigenvalues in the coefficients matrix of a linear equation system having a symmetric positive definite coefficients matrix. As derived and proved in prior literature, in exact arithmetic the conjugate gradient method always finds the exact solution to that linear equation system in less than or equal to $\aleph$ conjugate gradient steps (aka iterations).[S1,S2]

Strategy for solving the dipole resorption (DR) equations: As proved in Section 2.2.3 of this article's main text, the matrix $M^{(A)}$ is symmetric positive definite. As proved in Section S2 above, the matrix $M^{(A)}$ has 1, 2, 3, or 4 distinct eigenvalues. Therefore, it follows directly from the preceding paragraph that in exact arithmetic the conjugate gradient method converges to the exact solution of the linear equation system

$$M^{(A)}Y^{(A)} = T^{(A)}; \quad \sum_d M_{b,d}^{(A)} Y_d^{(A)} = T_b^{(A)} \tag{S76}$$

in less than or equal to four conjugate gradient steps (aka iterations). This provides an exact solution approach that is computationally efficient and does not require explicit construction of the matrix inverse. Furthermore, by using the vector-based expansion shown in eqn (S54), the matrix $M^{(A)}$ does not have to be explicitly allocated in memory.

Strategy for solving the quadrupole resorption (QR) equations: As defined in Section 2.2.4 of this article's main text and proved in Section S3 above, the matrix $C^{(A)}$ is symmetric positive definite. As proved in Section S3 above, the matrix $C^{(A)}$ has 1, 2, 3, 4, 5, 6, 7, or 8 distinct eigenvalues. Therefore, it directly follows from the first paragraph in this section that in exact arithmetic the conjugate gradient method converges to the exact solution of the linear equation system

$$C^{(A)}S^{(A)} = V^{(A)}; \quad \sum_{d} C^{(A)}_{b,d} S^{(A)}_{d} = V^{(A)}_{b} \tag{S77}$$

in less than or equal to eight conjugate gradient steps (aka iterations). This provides an exact solution approach that is computationally efficient and does not require explicit construction of the matrix inverse. Furthermore, by using the vector-based expansion shown in eqn (S68), the matrix $C^{(A)}$ does not have to be explicitly allocated in memory.

To maximize computational efficiency, our algorithm (see Figure S1) stores the large arrays in a way that facilitates using the array elements in cache line order (see ref S3 for a discussion of how to do this). These arrays are set up as follows:

(1) The stockholder net atomic charges are read from the input file and stored in an allocatable array named **net_atomic_charge(Natoms)** that has Natoms elements, where Natoms is the number of atoms in the material's unit cell.

(2) The stockholder atom-in-material dipole moments are read from the input file and stored in an allocatable array named **starting_atomic_dipole(3,Natoms)** whose first index has a value of 1 (for x component), 2 (for y component), or 3 (for z component) and whose second index is the atom number.

(3) The stockholder atom-in-material quadrupole moments are read from the input file and stored in an allocatable array named **atomic_quadrupole_tensor(6,Natoms)** whose first index has a value of 1 (for $x^2 - r^2/3$ component), 2 (for xy component), 3 (for xz component), 4 (for $y^2 - r^2/3$ component), 5 (for yz component), or 6 (for $z^2 - r^2/3$ component) and whose second index is the atom number.

(4) Figure S2 is Fortran-style pseudocode for two-pass read of the overlap population input information that sorts the OP_{Ab} information according to the index of atom A. In the first pass, the number of overlap populations for each atom A is incrementally counted via the allocated temporary array **temp_count_for_atoms(Natoms)**. In the second pass, the information for each overlap population is read in and stored in appropriate rows of the arrays **overlap_array_int_variables** and **overlap_array_real_variables**. This allows the input text file to contain the overlap populations in any unsorted order, but reads them in so that they are sorted so that OP_{Ab} related values for A being the first atom in the unit cell appear first in the stored arrays, followed by OP_{Ab} related values for A being the second atom in the unit cell, and so forth. Figure S2 illustrates a Fortran-style pseudocode for doing this. This creates three allocated arrays as follows:

overlap_array_int_variables(5,num_QDR_pairs) – num_QDR_pairs is the number of overlap population values in the **overlap_populations_and_contact_exchanges.txt** input file. The five integer values stored for each OP_{Ab} are: (1) index of the atom A (i.e., tenth atom in the material's unit cell is called atom '10', thirteenth atom is called atom '13', etc.), (2) index of the atom b, and (3–5) translation indices of the atom b's image.

overlap_array_real_variables(8,num_QDR_pairs) – The eight real numbers stored for each for each OP_{Ab} are: (1) the 2*tanh[2*OP_{Ab}] value, (2–4) the x, y, and z components of the bond direction $\hat{R}_{Ab}$, (5) the bond length R_{Ab} (in bohr), and (6–8) the x, y, and z components of the weighted bond direction $\left(2\tanh[2OP_{Ab}]\right)\hat{R}_{Ab}$.

start_value_for_atoms(Natoms+1) – This array stores the starting index for each atom. For example, if start_value_for_atoms(3) = 101 and start_value_for_atoms(4) = 178, then this means

that OP_{Ab} related values for A = atom '3' are stored in overlap_array_int_variables(:, 101:177) and overlap_array_real_variables(:, 101:177).

Following the set-up of input-information arrays as described above, the QDR charges are computed using parallelized shared memory code that implements a variant of the conjugate gradient (CG) method. In the 2-pass QDR procedure, the following arrays are first allocated: ALLOCATE(QDR_point_charge(natoms)), ALLOCATE(QDR_atomic_dipole(3,natoms)), ALLOCATE(Y_vector(num_QDR_pairs)), ALLOCATE(T_vector(num_QDR_pairs)), ALLOCATE(MP_vector(num_QDR_pairs)), ALLOCATE(P_vector(num_QDR_pairs)), ALLOCATE(TT_atom(natoms)). Then, selected variables and arrays are initialized as follows: QDR_point_charge = net_atomic_charge, QDR_atomic_dipole = starting_atomic_dipole, T_vector = 0.0, max_QDR_CG_iter = 0, max_CG_iter = 0, max_resid_L2_norm = 0.0. After initializing these variables, then the first pass of quadrupole resorption is performed (using the code shown in Figure S3). Figure S3 is an example implementation of OpenMP parallelized Fortran code that performs quadrupole resorption. Then, the first pass of dipole resorption is performed (using the code shown in Figure S4). Figure S4 is an example implementation of OpenMP parallelized Fortran code that performs dipole resorption. These codes are straightforward to follow and understand. A reduction accumulates the maximum number of CG steps (iterations) that were required across atoms in the material. Then, the second pass of quadrupole resorption is performed by again using the code shown in Figure S3. Finally, the second pass of dipole resorption is performed by again using the code shown in Figure S4. (To minimize memory requirements, the same large arrays are reused during dipole and quadrupole resorption. Specifically, the variables C, S, and V during quadrupole resorption are represented by the arrays M, Y, and T, respectively.)

After the QDR charges are computed, they are subsequently written to file. The following information is written to this output file: (a) the number of atoms in the material's unit cell, (b) periodic lattice vectors (if any) or a note that the system is nonperiodic (i.e., does not contain any periodic boundary conditions), (c) the element symbols, Cartesian XYZ coordinates, and QDR charges for each atom in the unit cell, (d) the sum of net atomic charges in the unit cell, (e) the software version, (f) the maximum number of CG steps (iterations) that were required across atoms in the material, (g) the maximum final L_2 (Euclidean) norm of the residual (which is approximately zero to machine precision), (h) how to cite the computational method and software program, (i) the residual atom-in-material dipole moments (i.e., $\left\{\vec{\mu}_A^{QDR}\right\}$) and their magnitudes, (j) the residual atom-in-material traceless quadrupole moments (i.e., $\left\{\vec{\vec{Q}}_A^{QDR}\right\}$), (k) if the system is nonperiodic (e.g., a molecule), then the total molecular dipole and traceless quadrupole moments are computed and printed first based on the $\left\{q_A^{QDR}\right\}$ values and molecular geometry, then based on the $\left\{q_A^{QDR},\vec{\mu}_A^{QDR}\right\}$ values and molecular geometry, and finally based on the $\left\{q_A^{QDR},\vec{\mu}_A^{QDR},\vec{\vec{Q}}_A^{QDR}\right\}$ values and molecular geometry, and (l) the date and time of computation.

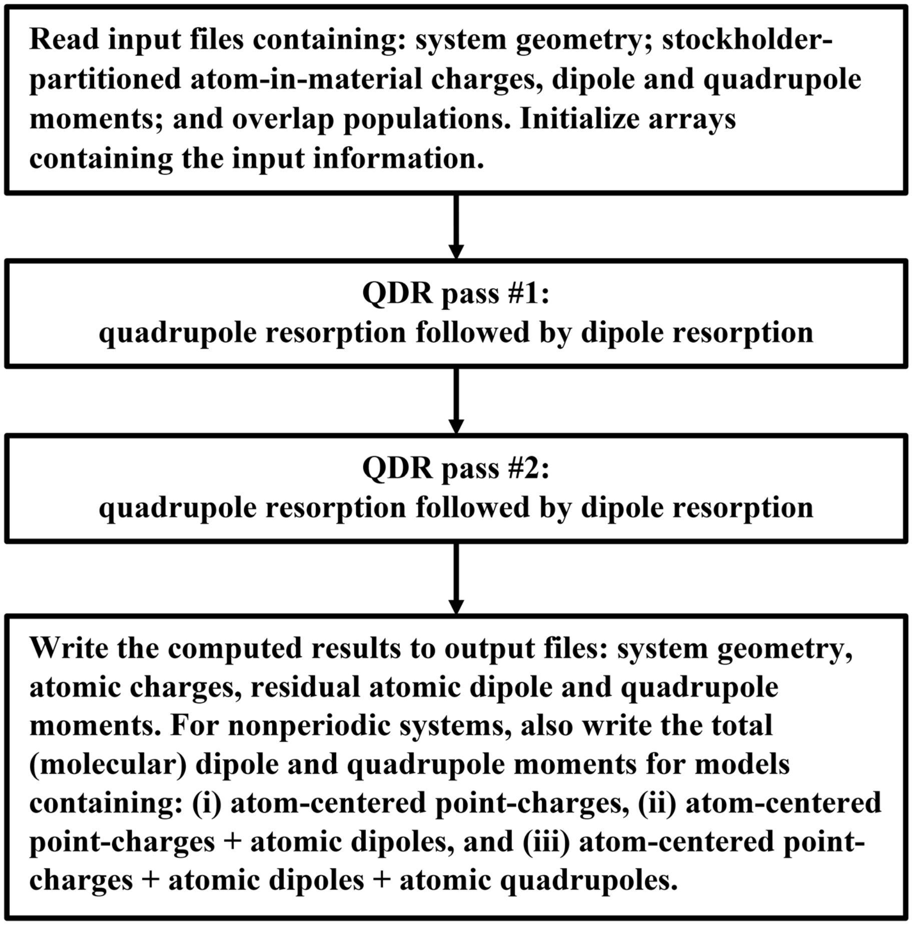

Figure S1: Overall flow diagram of steps in the 2-pass QDR computation.

```
! Pass #1
Open the input file containing the overlap populations
Read the number of overlap populations, assign this to the variable num_QDR_pairs
Read the remaining header lines
ALLOCATE(temp_count_for_atoms(natoms))
temp_count_for_atoms = 0  !Initialize
DO j=1,num_QDR_pairs
  READ(input_FID,*) atom1
  temp_count_for_atoms(atom1) = temp_count_for_atoms(atom1) + 1
END DO
Close the input file

! Set up the start_value_for_atoms array
ALLOCATE(start_value_for_atoms(natoms+1))
start_value_for_atoms(1) = 1
DO atom1=1,natoms
   start_value_for_atoms(atom1+1) = start_value_for_atoms(atom1) + temp_count_for_atoms(atom1)
END DO

! Pass #2
ALLOCATE(overlap_array_real_variables(8,num_QDR_pairs))
ALLOCATE(overlap_array_int_variables(5,num_QDR_pairs))
overlap_array_real_variables=0.0  !Initialize
overlap_array_int_variables=0  !Initialize
Open the input file containing the overlap populations
Read the header lines
temp_count_for_atoms = 0  !Reinitialize
DO j=1,num_QDR_pairs
  READ(input_FID,*) atom1,atom2,trans_a,trans_b,trans_c,overlap_population
  i = start_value_for_atoms(atom1) + temp_count_for_atoms(atom1)
  temp_count_for_atoms(atom1) = temp_count_for_atoms(atom1) + 1
  overlap_array_int_variables(1,i) = atom1
  overlap_array_int_variables(2,i) = atom2
  overlap_array_int_variables(3,i) = trans_a
  overlap_array_int_variables(4,i) = trans_b
  overlap_array_int_variables(5,i) = trans_c
  overlap_array_real_variables(1,i) = 2.0*tanh(2.0*overlap_population)
  !bond_vector_x
  bond_vector(1) = coords(1,atom2) - coords(1,atom1) + trans_a*vector1(1) + trans_b*vector2(1) + trans_c*vector3(1)
  !bond_vector_y
  bond_vector(2) = coords(2,atom2) - coords(2,atom1) + trans_a*vector1(2) + trans_b*vector2(2) + trans_c*vector3(2)
  !bond_vector_z
  bond_vector(3) = coords(3,atom2) - coords(3,atom1) + trans_a*vector1(3) + trans_b*vector2(3) + trans_c*vector3(3)
  !bond length
  overlap_array_real_variables(5,i) = SQRT(bond_vector(1)*bond_vector(1) &
  + bond_vector(2)*bond_vector(2) + bond_vector(3)*bond_vector(3))
  !bond direction
  overlap_array_real_variables(2:4,i)=(1.0/overlap_array_real_variables(5,i))*bond_vector(1:3)
  !weighted bond direction
  overlap_array_real_variables(6:8,i)=overlap_array_real_variables(1,i)*overlap_array_real_variables(2:4,i)
END DO
Close the input file
```

Figure S2: Fortran-style pseudocode for two-pass read of the overlap population information that sorts the OP_{Ab} information according to the index of atom A. This code sets up the following arrays: start_value_for_atoms, overlap_array_int_variables, and overlap_array_real_variables. *Color coding:* comments (green text), Fortran commands (black text), pseudocode summary instructions (red text).

```
!====================================Begin quadrupole resorption step
  Y_vector = 0.0  !Initialize
!$omp parallel do default(none) &
!$omp private(atom1,SV,EV,j,weighted_bond_dir,CG_iter,PMP,CG_alpha,TT_atom_old,CG_beta,temp_sum,temp_real, &
!$omp squared_distance,delta_charge,bond_direction) &
!$omp shared(natoms,start_value_for_atoms,overlap_array_int_variables,overlap_array_real_variables,T_vector, &
!$omp TT_atom,P_vector,MP_vector,Y_vector,QDR_atomic_dipole,atomic_quadrupole_tensor) &
!$omp reduction(max:max_CG_iter) &
!$omp schedule(static)
  DO atom1=1,natoms
    SV=start_value_for_atoms(atom1)
    EV=start_value_for_atoms(atom1+1)-1
    !Initialize the T_vector (the residual vector)
    DO j=SV,EV
      weighted_bond_dir = overlap_array_real_variables(6:8,j)
      bond_direction = overlap_array_real_variables(2:4,j)
      T_vector(j)= -weighted_bond_dir(1)*(atomic_quadrupole_tensor(1,atom1)*bond_direction(1) + &
      2.0*atomic_quadrupole_tensor(2,atom1)*bond_direction(2) + 2.0*atomic_quadrupole_tensor(3,atom1)*bond_direction(3))
      T_vector(j)= T_vector(j) - (atomic_quadrupole_tensor(4,atom1)*weighted_bond_dir(2)*bond_direction(2) + &
      2.0*atomic_quadrupole_tensor(5,atom1)*weighted_bond_dir(2)*bond_direction(3) + atomic_quadrupole_tensor(6,atom1)*weighted_bond_dir(3)*bond_direction(3))
    END DO
    !Initialize the TT
    TT_atom(atom1) = DOT_PRODUCT(T_vector(SV:EV),T_vector(SV:EV))
    !Initialize the P_vector (the CG search vector)
    P_vector(SV:EV) = T_vector(SV:EV)
    DO CG_iter = 1,10
      IF (SQRT(TT_atom(atom1)) .LE. 1.0E-12) EXIT  !Convergence reached for atom
      MP_vector(SV:EV) = P_vector(SV:EV)  !Part of the diagonal component product
      ! (-1/3) term
      temp_sum=0.0
      DO j=SV,EV
        temp_sum = temp_sum + overlap_array_real_variables(1,j)*P_vector(j)
      END DO
      temp_sum = -onethird*temp_sum
      MP_vector(SV:EV) = MP_vector(SV:EV) + temp_sum*overlap_array_real_variables(1,SV:EV)
      ! (X_A*X_b)(X_A*X_b) term
      temp_sum=0.0
      DO j=SV,EV
        temp_sum = temp_sum + overlap_array_real_variables(2,j)*overlap_array_real_variables(6,j)*P_vector(j)
      END DO
      MP_vector(SV:EV) = MP_vector(SV:EV) + temp_sum*overlap_array_real_variables(2,SV:EV)*overlap_array_real_variables(6,SV:EV)
      ! 2(X_A*X_b)(Y_A*Y_b) term
      temp_sum=0.0
      DO j=SV,EV
        temp_sum = temp_sum + overlap_array_real_variables(2,j)*overlap_array_real_variables(7,j)*P_vector(j)
      END DO
      temp_sum = 2.0*temp_sum
      MP_vector(SV:EV) = MP_vector(SV:EV) + temp_sum*overlap_array_real_variables(2,SV:EV)*overlap_array_real_variables(7,SV:EV)
      ! 2(X_A*X_b)(Z_A*Z_b) term
      temp_sum=0.0
      DO j=SV,EV
        temp_sum = temp_sum + overlap_array_real_variables(2,j)*overlap_array_real_variables(8,j)*P_vector(j)
      END DO
      temp_sum = 2.0*temp_sum
      MP_vector(SV:EV) = MP_vector(SV:EV) + temp_sum*overlap_array_real_variables(2,SV:EV)*overlap_array_real_variables(8,SV:EV)
      ! (Y_A*Y_b)(Y_A*Y_b) term
      temp_sum=0.0
      DO j=SV,EV
        temp_sum = temp_sum + overlap_array_real_variables(3,j)*overlap_array_real_variables(7,j)*P_vector(j)
      END DO
      MP_vector(SV:EV) = MP_vector(SV:EV) + temp_sum*overlap_array_real_variables(3,SV:EV)*overlap_array_real_variables(7,SV:EV)
      ! 2(Y_A*Y_b)(Z_A*Z_b) term
      temp_sum=0.0
      DO j=SV,EV
        temp_sum = temp_sum + overlap_array_real_variables(3,j)*overlap_array_real_variables(8,j)*P_vector(j)
      END DO
      temp_sum = 2.0*temp_sum
      MP_vector(SV:EV) = MP_vector(SV:EV) + temp_sum*overlap_array_real_variables(3,SV:EV)*overlap_array_real_variables(8,SV:EV)
      ! (Z_A*Z_b)(Z_A*Z_b) term
      temp_sum=0.0
      DO j=SV,EV
        temp_sum = temp_sum + overlap_array_real_variables(4,j)*overlap_array_real_variables(8,j)*P_vector(j)
      END DO
      MP_vector(SV:EV) = MP_vector(SV:EV) + temp_sum*overlap_array_real_variables(4,SV:EV)*overlap_array_real_variables(8,SV:EV)
      PMP = DOT_PRODUCT(P_vector(SV:EV),MP_vector(SV:EV))
      CG_alpha = TT_atom(atom1)/PMP
      Y_vector(SV:EV) = Y_vector(SV:EV) + CG_alpha*P_vector(SV:EV)
      TT_atom_old = TT_atom(atom1)
      T_vector(SV:EV) = T_vector(SV:EV) - CG_alpha*MP_vector(SV:EV)
      TT_atom(atom1) = DOT_PRODUCT(T_vector(SV:EV),T_vector(SV:EV))
      CG_beta = TT_atom(atom1)/TT_atom_old
      P_vector(SV:EV) = T_vector(SV:EV) + CG_beta*P_vector(SV:EV)
    END DO
    max_CG_iter=max((CG_iter-1),max_CG_iter)
    ! Update the atomic dipoles and atomic quadrupoles
    DO j=SV,EV
      temp_real=Y_vector(j)*overlap_array_real_variables(1,j)
      QDR_atomic_dipole(1:3,atom1) = QDR_atomic_dipole(1:3,atom1) &
      + (temp_real/overlap_array_real_variables(5,j))*overlap_array_real_variables(2:4,j)
      atomic_quadrupole_tensor(1,atom1) = atomic_quadrupole_tensor(1,atom1) + temp_real*(overlap_array_real_variables(2,j)*overlap_array_real_variables(2,j) - onethird)
      atomic_quadrupole_tensor(2,atom1) = atomic_quadrupole_tensor(2,atom1) + temp_real*overlap_array_real_variables(2,j)*overlap_array_real_variables(3,j)
      atomic_quadrupole_tensor(3,atom1) = atomic_quadrupole_tensor(3,atom1) + temp_real*overlap_array_real_variables(2,j)*overlap_array_real_variables(4,j)
      atomic_quadrupole_tensor(4,atom1) = atomic_quadrupole_tensor(4,atom1) + temp_real*(overlap_array_real_variables(3,j)*overlap_array_real_variables(3,j) - onethird)
      atomic_quadrupole_tensor(5,atom1) = atomic_quadrupole_tensor(5,atom1) + temp_real*overlap_array_real_variables(3,j)*overlap_array_real_variables(4,j)
      atomic_quadrupole_tensor(6,atom1) = atomic_quadrupole_tensor(6,atom1) + temp_real*(overlap_array_real_variables(4,j)*overlap_array_real_variables(4,j) - onethird)
    END DO
  END DO
!$omp end parallel do
  resid_L2_norm = SQRT(SUM(TT_atom))
  max_resid_L2_norm = max(max_resid_L2_norm,resid_L2_norm)
  max_QDR_CG_iter = max(max_QDR_CG_iter,max_CG_iter)
  ! Update the atomic charges
  DO j=1,num_QDR_pairs
    delta_charge=Y_vector(j)*overlap_array_real_variables(1,j)/(overlap_array_real_variables(5,j)*overlap_array_real_variables(5,j))
    atom1 = overlap_array_int_variables(1,j)
    atom2 = overlap_array_int_variables(2,j)
    QDR_point_charge(atom1) = QDR_point_charge(atom1) + delta_charge
    QDR_point_charge(atom2) = QDR_point_charge(atom2) - delta_charge
  END DO
!====================================End quadrupole resorption step
```

Figure S3: OpenMP parallelized Fortran code that performs quadrupole resorption. *Color coding:* comments (green text), Fortran commands (black text), OpenMP directives (blue text).

```
!====================================Begin dipole resorption step
Y_vector = 0.0  !Initialize
!$omp parallel do default(none) &
!$omp private(atom1,SV,EV,j,weighted_bond_dir,CG_iter,PMP,CG_alpha,TT_atom_old,CG_beta,temp_sum) &
!$omp shared(natoms,start_value_for_atoms,overlap_array_int_variables,overlap_array_real_variables,T_vector, &
!$omp TT_atom,P_vector,MP_vector,Y_vector,QDR_atomic_dipole) &
!$omp reduction(max:max_CG_iter) &
!$omp schedule(static)
  DO atom1=1,natoms
    SV=start_value_for_atoms(atom1)
    EV=start_value_for_atoms(atom1+1) - 1
    !Initialize the T_vector (the residual vector)
    DO j=SV,EV
      weighted_bond_dir = overlap_array_real_variables(6:8,j)
      T_vector(j)= -(QDR_atomic_dipole(1,atom1)*weighted_bond_dir(1) + QDR_atomic_dipole(2,atom1)*weighted_bond_dir(2) &
      + QDR_atomic_dipole(3,atom1)*weighted_bond_dir(3))
    END DO
    !Initialize the TT
    TT_atom(atom1) = DOT_PRODUCT(T_vector(SV:EV),T_vector(SV:EV))
    !Initialize the P_vector (the CG search vector)
    P_vector(SV:EV) = T_vector(SV:EV)
    DO CG_iter = 1,10
      IF (SQRT(TT_atom(atom1)) .LE. 1.0E-12) EXIT  !Convergence reached for atom
      MP_vector(SV:EV) =  P_vector(SV:EV)  !Part of the diagonal component product
      temp_sum=0.0
      DO j=SV,EV
        temp_sum = temp_sum + overlap_array_real_variables(6,j)*P_vector(j)
      END DO
      MP_vector(SV:EV) =  MP_vector(SV:EV) + temp_sum*overlap_array_real_variables(6,SV:EV)
      temp_sum=0.0
      DO j=SV,EV
        temp_sum = temp_sum + overlap_array_real_variables(7,j)*P_vector(j)
      END DO
      MP_vector(SV:EV) =  MP_vector(SV:EV) + temp_sum*overlap_array_real_variables(7,SV:EV)
      temp_sum=0.0
      DO j=SV,EV
        temp_sum = temp_sum + overlap_array_real_variables(8,j)*P_vector(j)
      END DO
      MP_vector(SV:EV) =  MP_vector(SV:EV) + temp_sum*overlap_array_real_variables(8,SV:EV)
      PMP = DOT_PRODUCT(P_vector(SV:EV),MP_vector(SV:EV))
      CG_alpha = TT_atom(atom1)/PMP
      Y_vector(SV:EV) = Y_vector(SV:EV) + CG_alpha*P_vector(SV:EV)
      TT_atom_old = TT_atom(atom1)
      T_vector(SV:EV) = T_vector(SV:EV) - CG_alpha*MP_vector(SV:EV)
      TT_atom(atom1) = DOT_PRODUCT(T_vector(SV:EV),T_vector(SV:EV))
      CG_beta = TT_atom(atom1)/TT_atom_old
      P_vector(SV:EV) = T_vector(SV:EV) + CG_beta*P_vector(SV:EV)
    END DO
    max_CG_iter=max((CG_iter-1),max_CG_iter)
  END DO
!$omp end parallel do
  resid_L2_norm = SQRT(SUM(TT_atom))
  max_resid_L2_norm = max(max_resid_L2_norm,resid_L2_norm)
  max_QDR_CG_iter = max(max_QDR_CG_iter,max_CG_iter)
  ! Update the atomic charges and atomic dipoles
  DO j=1,num_QDR_pairs
    delta_charge = Y_vector(j)*overlap_array_real_variables(1,j)/overlap_array_real_variables(5,j)
    atom1 = overlap_array_int_variables(1,j)
    atom2 = overlap_array_int_variables(2,j)
    QDR_point_charge(atom1) = QDR_point_charge(atom1) + delta_charge
    QDR_point_charge(atom2) = QDR_point_charge(atom2) - delta_charge
    temp_real=0.5*delta_charge*overlap_array_real_variables(5,j)
    QDR_atomic_dipole(1:3,atom1) = QDR_atomic_dipole(1:3,atom1) + temp_real*overlap_array_real_variables(2:4,j)
    QDR_atomic_dipole(1:3,atom2) = QDR_atomic_dipole(1:3,atom2) + temp_real*overlap_array_real_variables(2:4,j)
  END DO
!====================================End dipole resorption step
```

Figure S4: OpenMP parallelized Fortran code that performs dipole resorption. *Color coding:* comments (green text), Fortran commands (black text), OpenMP directives (blue text).

S5. Supplementary tables

Table S1: Chemical formula and compound class for each molecule included in the organics test set. The last column indicates whether the molecule contains any rotatable dihedrals.

name	chemical formula	compound class	rotatable dihedrals
1-bromo-4-(trifluoromethyl) benzene	$C_7H_4BrF_3$	haloarene	yes
1-bromopropane	C_3H_7Br	haloalkane	yes
1,2-dichloropropane	$C_3H_6Cl_2$	haloalkane	yes
1H-indole	C_8H_7N	aromatic heterocycle	no
2-butyne	C_4H_6	alkyne	yes
2-methylbutane	C_5H_{12}	alkane	yes
acetamide	C_2H_5NO	amide	yes
acetate ion	$[C_2H_3O_2]^-$	organic ion	yes
acetic acid	$C_2H_4O_2$	carboxylic acid	yes
acetone	C_3H_6O	ketone	yes
acetonitrile	C_2H_3N	nitrile	yes
adenine	$C_5H_5N_5$	nucleobase	yes
alanine	$C_3H_7NO_2$	amino acid	yes
aniline	C_6H_7N	primary amine	yes
arginine	$C_6H_{14}N_4O_2$	amino acid	yes
ATP	$C_{10}H_{16}N_5O_{13}P_3$	phosphate groups	yes
chlorobenzene	C_6H_5Cl	haloarene	no
chlorofluoromethane	CH_2ClF	haloalkane	no
chloroform	$CHCl_3$	haloalkane	no
cyclohexane	C_6H_{12}	alkane	no
cystine	$C_6H_{12}N_2O_4S_2$	biomolecule	yes
diethyl amine	$C_4H_{11}N$	secondary amine	yes
diethyl ether	$C_4H_{10}O$	ether	yes
dimethyl carbonate	$C_3H_6O_3$	organic carbonate	yes
dimethyl ether	C_2H_6O	ether	yes
dimethyl sulfoxide (DMSO)	C_2H_6OS	sulfoxide	yes
DNA base guanine	$[C_{12}H_{17}N_5O_{10}P_2]^{2-}$	biomolecule	yes
ethanethiol	C_2H_6S	thiol	yes
ethanol	C_2H_6O	alcohol	yes
ethylacetate	$C_4H_8O_2$	ester	yes
ethylamine	C_2H_7N	primary amine	yes
ethylammonium ion	$[C_2H_8N]^+$	organic ion	yes
ethylformamide	C_3H_7NO	amide	yes
ethylmagnesium bromide	C_2H_5BrMg	Grignard reagent	yes
fluorobenzene	C_6H_5F	haloarene	no
formaldehyde	CH_2O	aldehyde	no
fructose	$C_6H_{12}O_6$	sugar	yes
furan	C_4H_4O	aromatic heterocycle	no
hydroxybicyclo octane carboxylic acid	$C_9H_{14}O_3$	carboxylic acid	yes
iodobenzene	C_6H_5I	haloarene	no
$N@C_{60}$	$N@C_{60}$	endohedral complex	no
n-methyl-acetamide	C_3H_7NO	amide	yes
naphthalene	$C_{10}H_8$	aromatic	no
nitrobenzene	$C_6H_5NO_2$	nitro	yes
oxetane	C_3H_6O	heterocycle	no
phenol	C_6H_6O	aromatic alcohol	yes
phospholipid	$C_{14}H_{28}NO_8P$	biomolecule	yes
propane	C_3H_8	alkane	yes
propene	C_3H_6	alkene	yes

pyridine	C_5H_5N	azaarene	no
thioanisole	C_7H_8S	thioether	yes
thiophene	C_4H_4S	aromatic heterocycle	no
toluene	C_7H_8	arene	yes
trimethyl amine	C_3H_9N	tertiary amine	yes

Table S2: Molecules included in the inorganic molecules test set. The last column indicates whether the molecule contains any rotatable dihedrals.

name	chemical formula	rotatable dihedrals
dialuminium hexachloride	Al_2Cl_6	no
chlorine trifluoride	ClF_3	no
carbon dioxide	CO_2	no
fluoro trihydroxy silane	FH_3O_3Si	yes
water	H_2O	no
hydrogen sulfide	H_2S	no
sulfurous acid	H_2SO_3	yes
arsenic acid	H_3AsO_4	yes
phosphorous acid	H_3PO_3	yes
chlorous acid	$HClO_2$	yes
hydrogen cyanide	HCN	no
iodic acid	HIO_3	yes
nitroxyl	HNO	no
nitrous acid	HNO_2	yes
lithium oxide	Li_2O	no
ammonia	NH_3	no
phosphine	PH_3	no
sulfur tetraflouride	SF_4	no
sulfur dioxide	SO_2	no
xenon oxytetrafluoride	$XeOF_4$	no

Table S3: Molecules included in the transition metal complexes test set. The last column indicates whether the molecule contains any rotatable dihedrals.

name	chemical formula	rotatable dihedrals
molybdenum acetate	$C_8H_{12}O_8Mo_2$	yes
cisplatin	$PtCl_2(NH_3)_2$	yes
CpTi(CAr)Me_2	$C_{15}H_{20}OTi$	yes
CpTi(SAr)Me_2	$C_{16}H_{22}STi$	yes
chromium hexacyanide anion	$[Cr(CN)_6]^{3-}$	no
osmium hexafluoride	OsF_6	no
zirconium trichloride hydroxide	$ZrCl_3(OH)$	yes

Table S4: Structures in the nanoporous solids test set. ALPO = aluminum phosphate; COF = covalent organic framework; MOF = metal-organic framework; MIF = metal inorganic framework.

**name**	**chemical formula**	**framework type**	**a (Å)**	**b (Å)**	**c (Å)**	**α (º)**	**β (º)**	**γ (º)**
AlPO-5	$Al_{12}O_{48}P_{12}$	ALPO	13.74	13.74	8.47	90.0	90.0	120.0
BF-COF-2	$C_{66}H_{60}N_{12}O_{12}$	COF	16.23	16.23	16.25	90.0	90.0	90.0
BN nanotube	$B_{20}N_{20}$	nanotube	4.36	16.00	16.00	90.0	90.0	90.0
chabazite	$Si_{12}O_{24}$	zeolite	9.47	9.47	9.47	94.4	94.4	94.4
HENYUV	$C_{28}H_8N_4O_{16}Zn_4$	MOF	7.03	7.03	19.94	90.0	90.0	90.0
IRMOF-1	$C_{48}H_{24}O_{26}Zn_8$	MOF	18.31	18.31	18.31	60.0	60.0	60.0
MECWEX	$C_{48}H_{60}N_{24}Zn_6$	MOF	14.73	14.73	14.73	109.5	109.5	109.5
Mg-MOF-74	$C_{72}H_{18}Mg_{18}O_{54}$	MOF	26.14	26.14	6.94	90.0	90.0	120.0
MIL-74	$Al_9O_{48}P_{12}$	ALPO	14.52	14.52	14.52	109.5	109.5	109.5
SIZ-3	$Al_{10}F_2O_{40}P_{10}$	MIF	8.42	12.13	12.13	66.4	69.7	69.7
ZIF-8	$C_{96}H_{120}N_{48}Zn_{12}$	MOF	17.16	17.16	17.16	90.0	90.0	90.0

S6. Supplementary raincloud plots

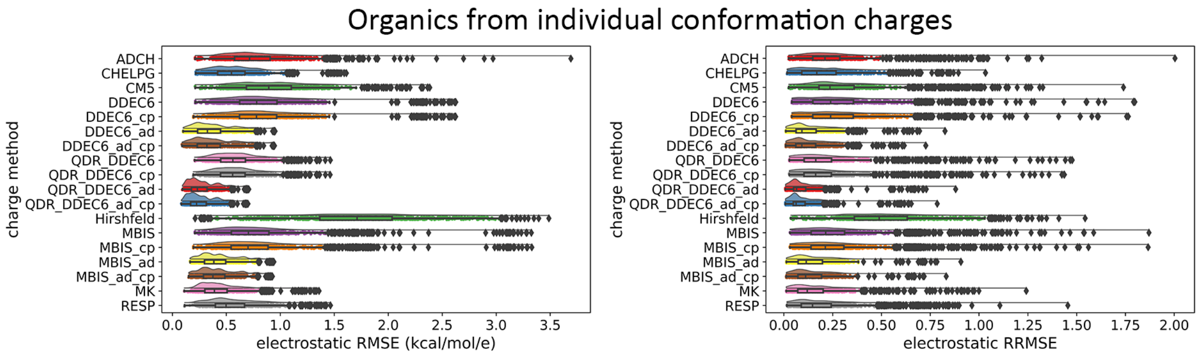

Figure S5: Raincloud plots for the organic molecules dataset for different charge assignment methods using individual conformation charges. This contains all materials and geometries in the organics training dataset.

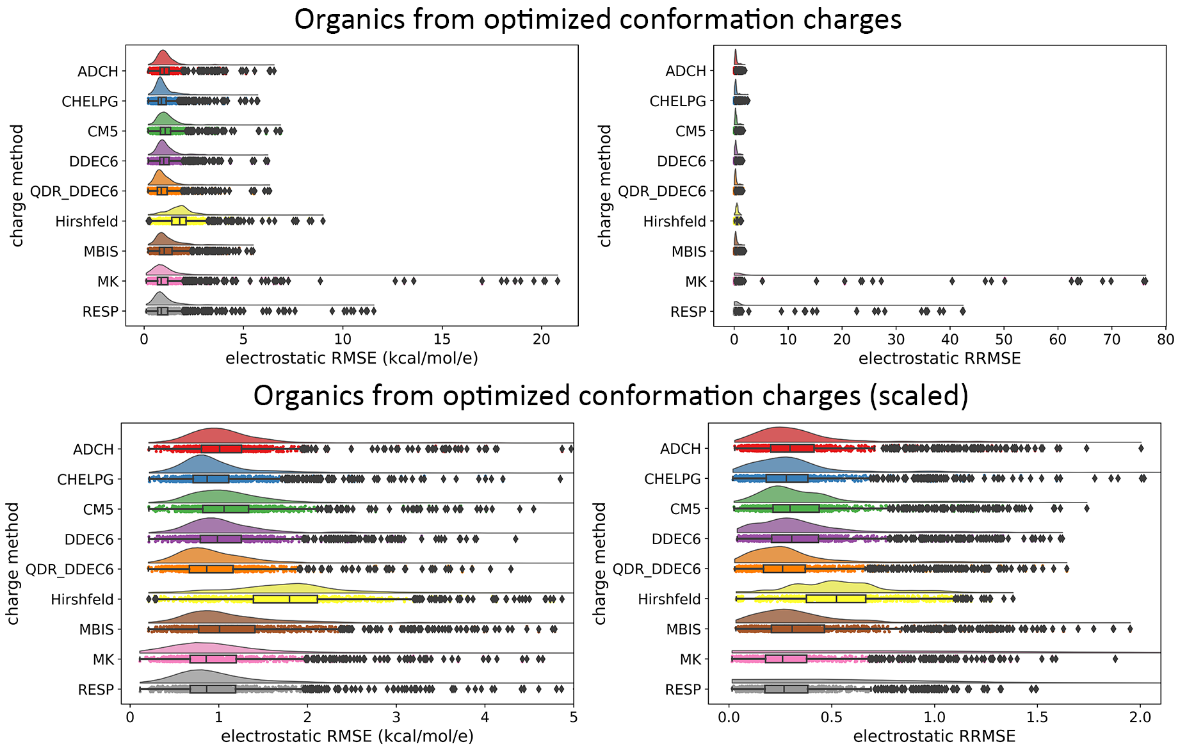

Figure S6: Raincloud plots for the organic molecules dataset for different charge assignment methods using the optimized ground-state conformation charges. The bottom row uses an enlarged scale to zoom in on the results. This contains all materials and geometries in the organics training dataset. The outliers having RMSE > 5.0 kcal/mol/e were for the ATP and N@C_{60} molecules. The outliers having RRMSE > 2.0 were for the N@C_{60} molecule.

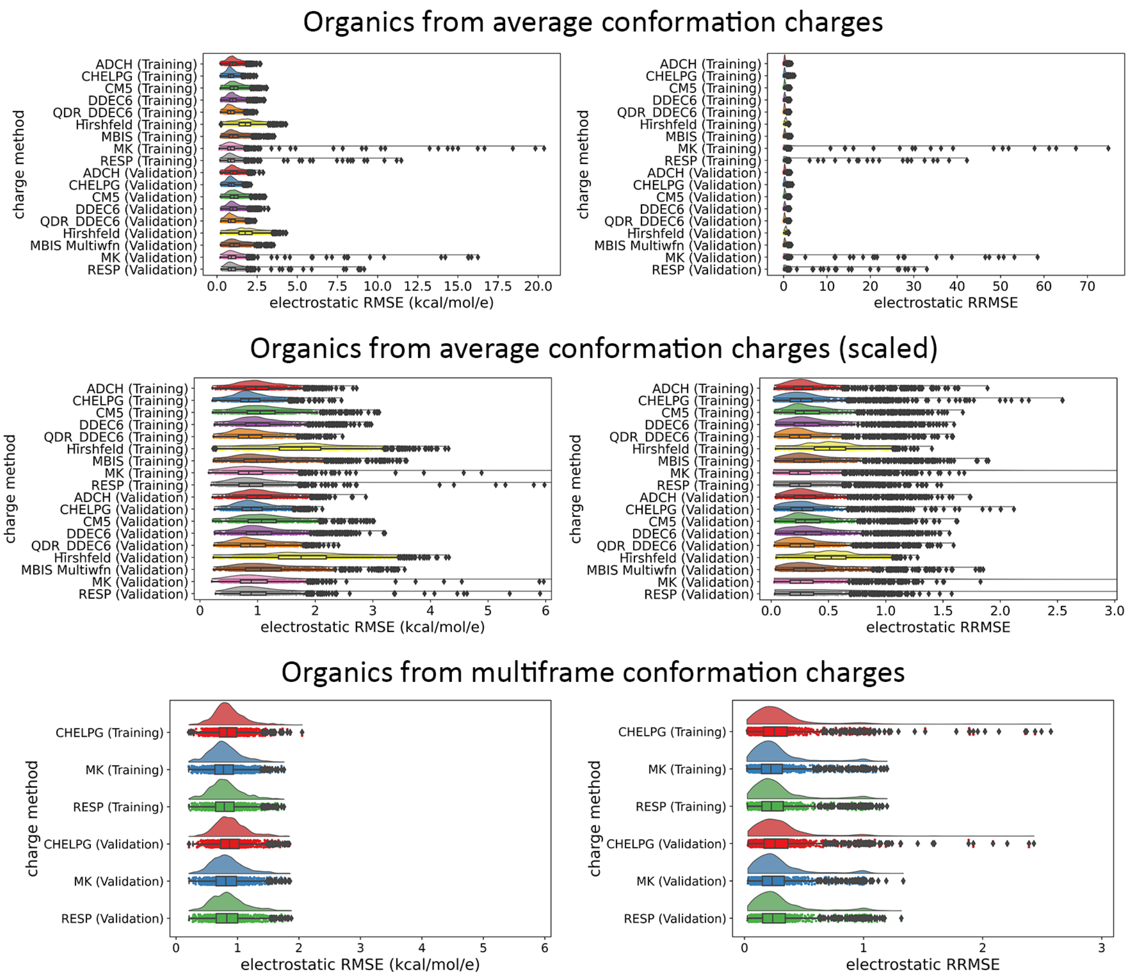

Figure S7: Raincloud plots for the organic molecules dataset using average conformation charges (top and middle rows) and using multiframe charges (bottom row) for different charge assignment methods. The middle row uses an enlarged scale to zoom in on the results. These raincloud plots include training and validation data. For the average conformation or multiframe charges, the outliers having RMSE > 5.0 kcal/mol/e and/or RRMSE > 2.0 were for N@C_{60} molecule.

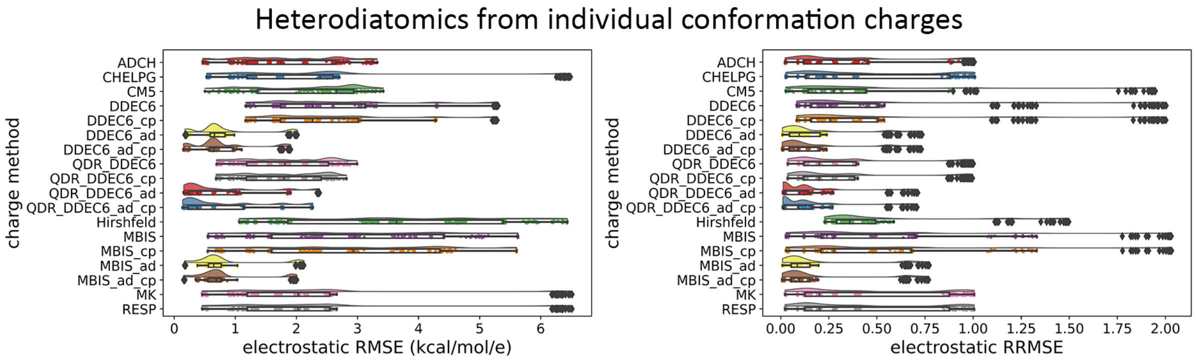

Figure S8: Raincloud plots for the heterodiatomic molecules dataset for different charge assignment methods using individual conformation charges. The data shown here is that of the training set. Outliers having RMSE > 5.0 kcal/mol/e were for the BaS, LiCs, NaCl, and SrO molecules. Outliers having RRMSE > 2.0 were for the CO molecule.

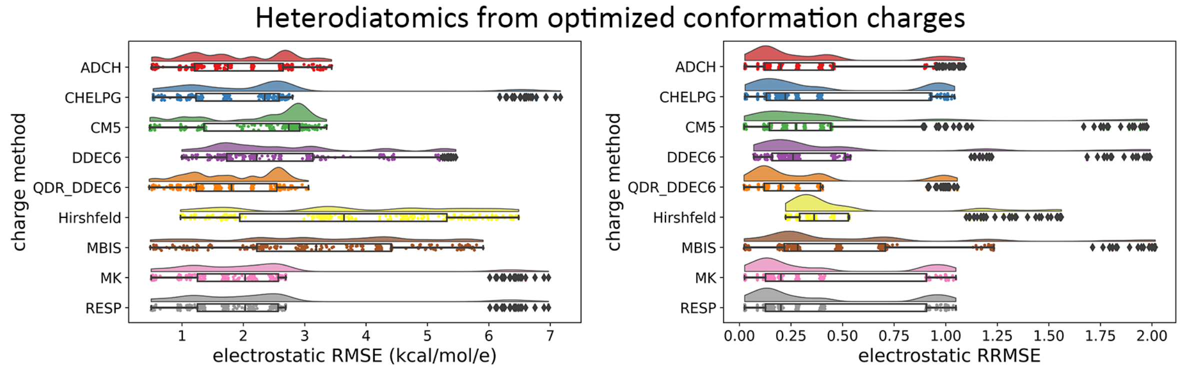

Figure S9: Raincloud plots for the heterodiatomic molecules dataset for different charge assignment methods using the optimized ground-state conformation charges. This contained all materials and geometries in the heterodiatomics training dataset. Outliers having RMSE > 5.0 kcal/mol/e were for the BaS, KF, LiCs, NaCl, and SrO molecules. Outliers having RRMSE > 2.0 were for the CO molecule.

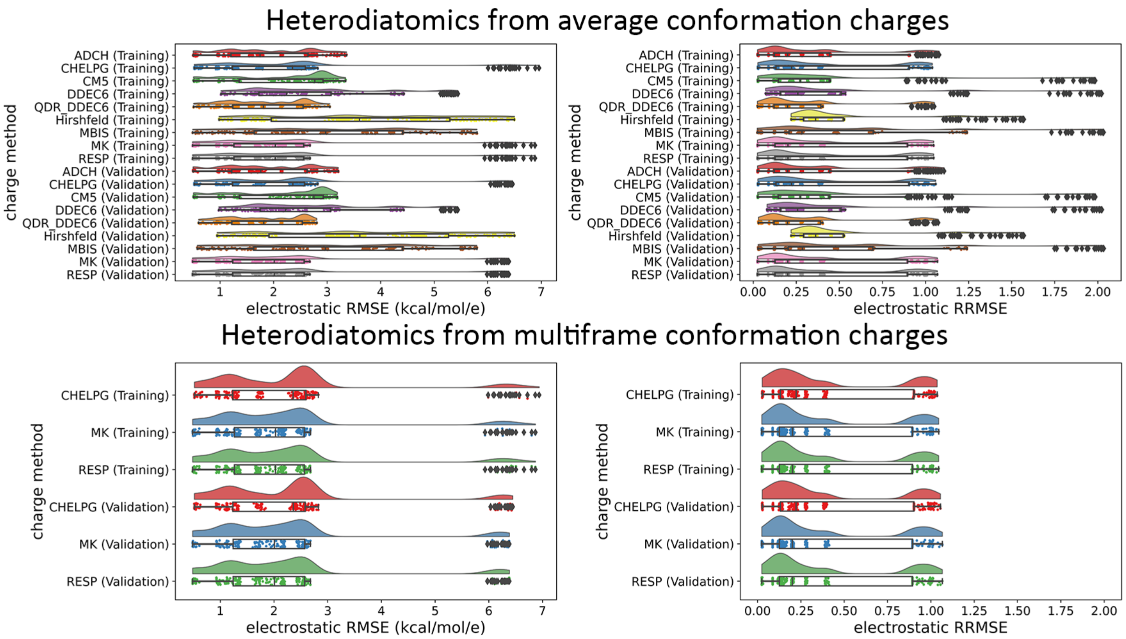

Figure S10: Raincloud plots for the heterodiatomic molecules dataset using average conformation charges (top panels) and using multiframe charges (bottom panels) for different charge assignment methods. These raincloud plots include training and validation data. For the average conformation charges, the outliers having RMSE > 5.0 kcal/mol/e were the BaS, KF, LiCs, NaCl, and SrO molecules, and the outliers having RRMSE > 2.0 were for the CO molecule. For the multiframe charges, the outliers having RMSE > 5.0 kcal/mol/e were for the LiCs molecule.

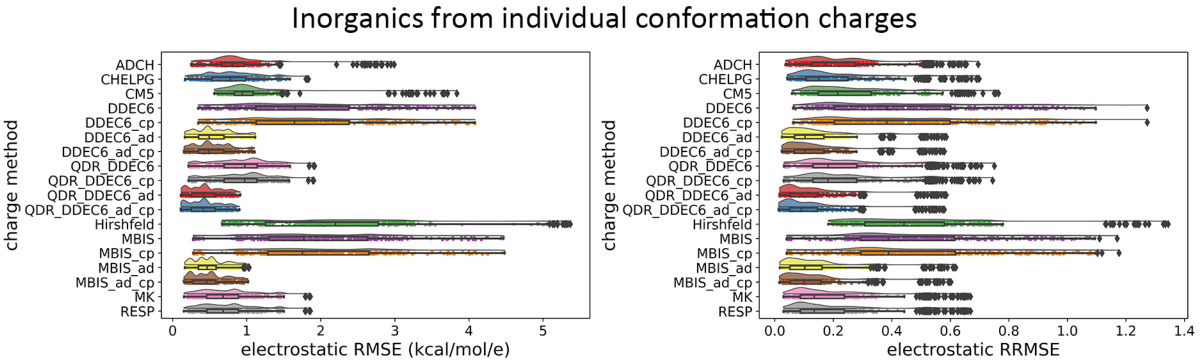

Figure S11: Raincloud plots for the inorganic molecules dataset for different charge assignment methods using individual conformation charges. This contains all materials and geometries in the inorganics training dataset. The outliers having RMSE > 5.0 kcal/mol/e were for the Hirshfeld method applied to the Li_2O molecule.

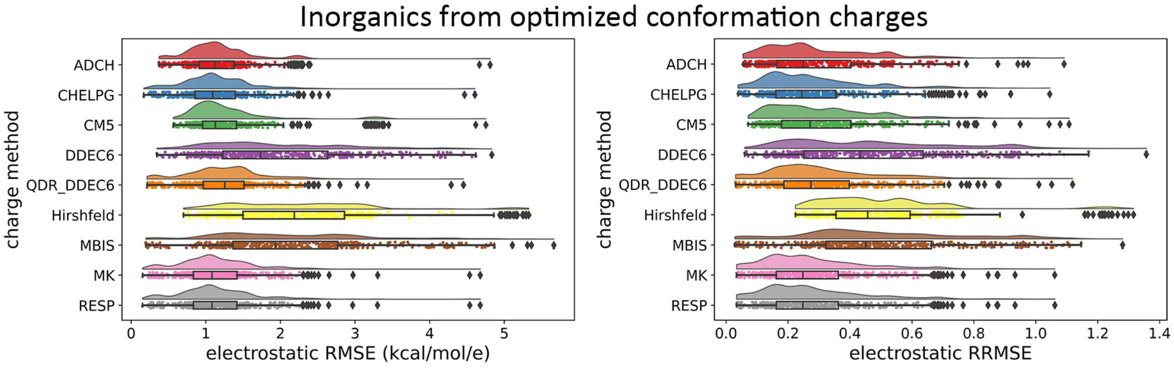

Figure S12: Raincloud plots for the inorganic molecules dataset for different charge assignment methods using the optimized ground-state conformation charges. This contained all materials and geometries in the inorganics training dataset. Outliers having RMSE > 5.0 kcal/mol/e were for the Li_2O and HIO_3 molecules.

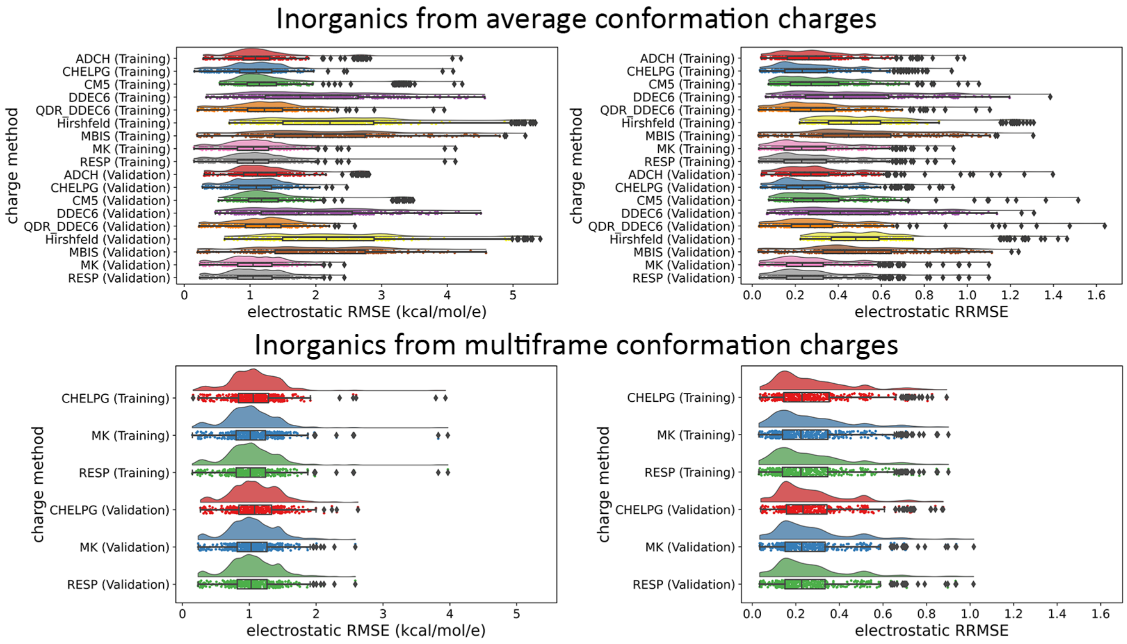

Figure S13: Raincloud plots for the inorganic molecules dataset using average conformation charges (top panels) and using multiframe charges (bottom panels) for different charge assignment methods. These raincloud plots include training and validation data. For the average conformation charges, the outliers having RMSE > 5.0 kcal/mol/e were for the Li_2O and HIO_3 molecules.

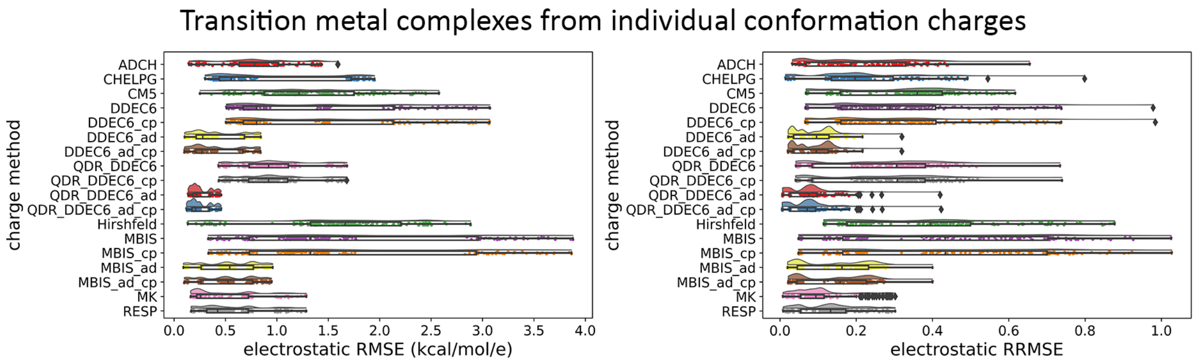

Figure S14: Raincloud plots for the transition metal complexes for different charge assignment methods using individual conformation charges. This contained all materials and geometries in the transition metal complexes training dataset.

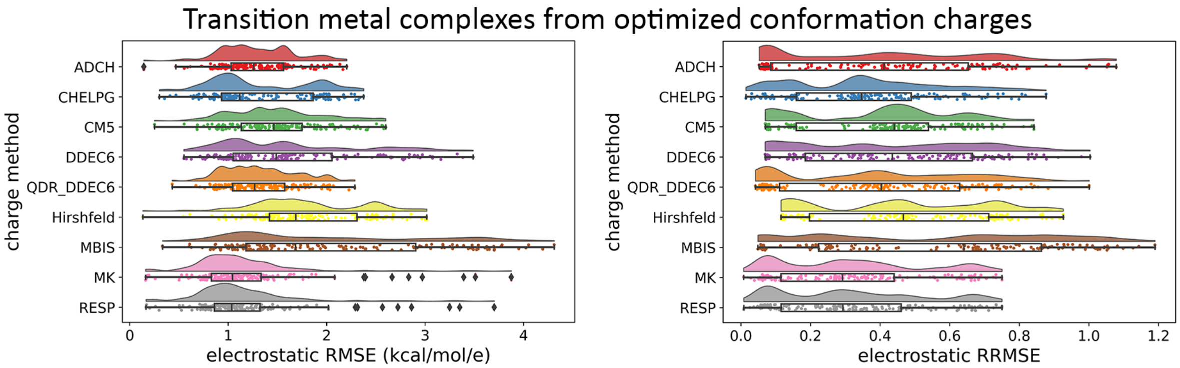

Figure S15: Raincloud plots for the transition metal complexes for different charge assignment methods using the optimized ground-state conformation charges. This contained all materials and geometries in the transition metal complexes training dataset.

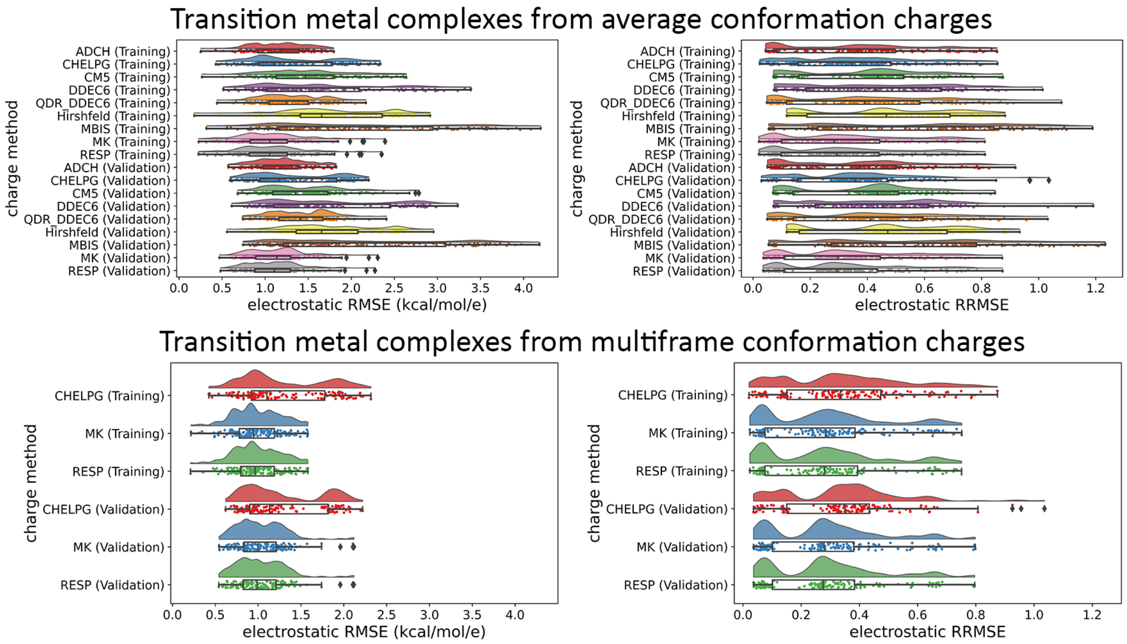

Figure S16: Raincloud plots for the transition metal complexes using average conformation charges (top panels) and using multiframe charges (bottom panels) for different charge assignment methods. These raincloud plots include training and validation data.

References:

S1. J. R. Shewchuk, An Introduction to the Conjugate Gradient Method Without the Agonizing Pain, technical report, Carnegie Mellon University, Pittsburgh, Pennsylvania, 1994, pp. 1-64.

S2. M. R. Hestenes and E. Stiefel, Methods of conjugate gradients for solving linear systems, *J. Res. Natl. Bur. Stand. (U.S.)*, 1952, **49**, 409-436, DOI: 10.6028/jres.049.044.

S3. N. Gabaldon Limas and T. A. Manz, Introducing DDEC6 atomic population analysis: part 4. Efficient parallel computation of net atomic charges, atomic spin moments, bond orders, and more, *RSC Adv.*, 2018, **8**, 2678-2707, DOI: 10.1039/c7ra11829e.
